# Author Correction: Safety outcomes following COVID-19 vaccination and infection in 5.1 million children in England

**DOI:** 10.1038/s41467-024-50151-0

**Published:** 2024-07-08

**Authors:** Emma Copland, Martina Patone, Defne Saatci, Lahiru Handunnetthi, Jennifer Hirst, David P. J. Hunt, Nicholas L. Mills, Paul Moss, Aziz Sheikh, Carol A. C. Coupland, Anthony Harnden, Chris Robertson, Julia Hippisley-Cox

**Affiliations:** 1 Nuffield Department of Primary Health Care Sciences, University of Oxford, Oxford, UK; 2 Centre for Human Genetics, University of Oxford, Oxford, UK; 3 Department of Psychiatry, University of Oxford, Oxford, UK; 4 UK Dementia Research Institute, Centre for Clinical Brain Sciences, University of Edinburgh, Edinburgh, UK; 5 BHF/University Centre for Cardiovascular Science, University of Edinburgh, Edinburgh, UK; 6 Usher Institute, University of Edinburgh, Edinburgh, UK; 7 Institute of Immunology and Immunotherapy, University of Birmingham, Birmingham, UK; 8 Centre for Academic Primary Care, School of Medicine, University of Nottingham, Nottingham, UK; 9 Department of Mathematics and Statistics, University of Strathclyde, Glasgow, UK

**Keywords:** Epidemiology, Public health, Vaccines, SARS-CoV-2

Correction to: *Nature Communications* 10.1038/s41467-024-47745-z, published online 27 May 2024

The original version of this article contained information on vaccine dose amount (full or half) in Table 2 and Supplementary Table 1. This has subsequently been found to be inaccurate due to inconsistent coding in the raw data. The vaccine dose amount has therefore been removed from Table 2 and Supplementary Table 1. This data was not used elsewhere in the analysis. An additional line has been added to the introduction to summarise vaccine doses that were given to the population:

“Those aged 12 years and above were given a full dose of BNT162b2 vaccine (30 micrograms), and children aged 5–11 years were given a dose of 10 micrograms of BNT162b2 vaccine.”

These changes have been made in the PDF and HTML versions of the article.

### Supplementary information

Supplementary Table 1

