# Supplementary material for: Safety outcomes following COVID-19 vaccination and infection in 5.1 million children in England
Source: Nat Commun. 2024 May 27;15:3822. doi: 10.1038/s41467-024-47745-z (PMC11130197; doi:10.1038/s41467-024-47745-z)
Supplement: Supplementary file 1 — Supplementary Information [file 41467_2024_47745_MOESM1_ESM.pdf]

Supplementary Material

Safety outcomes following COVID-19 vaccination and infection in 5.1 million children in England

Emma Copland [1], Martina Patone [1], Defne Saatci [1], Lahiru Handunnetthi [2,3], Jennifer Hirst [1], David P J Hunt [4], Nicholas L Mills [5,6], Paul Moss [7], Aziz Sheikh [1,6], Carol AC Coupland [1,8], Anthony Harnden [1], Chris Robertson [9], Julia Hippisley-Cox* [1]

1: Nuffield Department of Primary Health Care Sciences, University of Oxford, UK

2. Centre for Human Genetics, University of Oxford, UK

3. Department of Psychiatry, University of Oxford, UK

4: UK Dementia Research Institute, Centre for Clinical Brain Sciences, University of Edinburgh, UK

5: BHF/University Centre for Cardiovascular Science, University of Edinburgh, UK

6: Usher Institute, University of Edinburgh, UK

7. Institute of Immunology and Immunotherapy, University of Birmingham, UK

8: Centre for Academic Primary Care, School of Medicine, University of Nottingham, UK

9. Department of Mathematics and Statistics, University of Strathclyde, Glasgow, UK

Correspondence to: Professor Julia Hippisley-Cox, Nuffield Department of Primary Care Health Sciences, Radcliffe Observatory Quarter, Woodstock Road, Oxford

Email: julia.hippisley-cox@phc.ox.ac.uk

Contents

Supplementary Figures 4

Supplementary Figure 1. Risk of serious outcomes following a first or second dose of COVID-19 vaccination with the BNT162b2 vaccine or SARS-CoV-2 infection (prior to vaccination) in male adolescents aged 12-17 years. 4

Supplementary Figure 2. Risk of serious outcomes following a first or second dose of COVID-19 vaccination with the BNT162b2 vaccine or SARS-CoV-2 infection (prior to vaccination) in female adolescents aged 12-17 years. 5

Supplementary Figure 3. Risk of serious outcomes following SARS-CoV-2 infection prior to vaccination in male children aged 5-11 years. 6

Supplementary Figure 4. Risk of serious outcomes following SARS-CoV-2 infection prior to vaccination in female children aged 5-11 years. 7

Supplementary Tables 8

Supplementary Table 1. Characteristics of young people aged 5-11 years, 12-17 years and 18-24 years receiving either ChAdOx1, BNT162b2 or mRNA-1273 vaccines or testing positive for SARS-CoV-2 in England between 8th December 2020 and 7th August 2022. 8

Supplementary Table 2. Baseline demographic characteristics of cohort in young people who did not receive any dose of COVID-19 vaccine and did not have a positive SARS-CoV-2 test recorded in England between 8th December 2020 and 7th August 2022, stratified by age group: 5-11, 12-17 and 18-24 years. 11

Supplementary Table 3. Number of patients who were admitted to hospital or died from each outcome in the baseline period or the 1-42 days following vaccination or SARS-CoV-2 positive test. 12

Supplementary Table 4. Incidence rate ratios and estimated number of excess events per million for individual outcomes in the 1-42 days following vaccination or a SARS-CoV-2 positive test (before or after vaccination) compared to baseline period in females aged 5-11 and 12-17 years. 17

Supplementary Table 5. Incidence rate ratios and estimated number of excess events per million for individual outcomes in the 1-42 days following vaccination or a SARS-CoV-2 positive test (before or after vaccination) compared to baseline period in males aged 5-11 and 12-17 years. 21

Supplementary Table 6. Risks of individual outcomes in the 1-42 days following vaccination or a SARS-CoV-2 positive test (before or after vaccination) compared to baseline period in young people aged 5-11, 12-17 and 18-24 years of white and non-white ethnicity. 25

Supplementary Table 7. Incidence rate ratios and estimated number of excess events per million in pre-specified weekly periods in the 1-42 days following vaccination or a SARS-CoV-2 positive test (before or after vaccination) compared to baseline period in children aged 5-11 years. 37

Supplementary Table 8. Incidence rate ratios and estimated number of excess events per million in pre-specified weekly periods in the 1-42 days following vaccination or a SARS-CoV-2 positive test (before or after vaccination) compared to baseline period in adolescents aged 12-17 years. 42

Supplementary Table 9. Incidence rate ratios and estimated number of excess events per million for individual outcomes in the 1-42 days following vaccination or a SARS-CoV-2 positive test (before or after vaccination) compared to baseline period in young adults aged 18-24 years, and stratified by sex. 49

Supplementary Table 10. Characteristics of unvaccinated and vaccinated young people and those testing positive for SARS-CoV-2 aged 5-11, 12-17 and 18-24 years included in the QResearch primary care database in England between 8th December 2020 until 7th August 2022. 55

Supplementary Table 11. Incidence rates of individual outcomes (events per 100,000 person-years) in the 1-42 days following vaccination or a SARS-CoV-2 positive test (before or after vaccination) in young people aged 5-11, 12-17 and 18-24 years included in QResearch primary care database in England between 8th December 2020 until 7th August 2022. 58

Supplementary Table 12. Characteristics of matched vaccinated and unvaccinated young people aged 5-11, 12-17 and 18-24 years included in matched cohort study included in QResearch primary care database in England between 8th December 2020 until 7th August 2022. 62

Supplementary Table 13. Incidence rate ratios (unadjusted and adjusted for ethnicity, quintile of deprivation and comorbidities) for individual outcomes in the 1-42 days following vaccination compared to matched unvaccinated group in young people aged 5-11, 12-17 and 18-24 years. 64

Supplementary Figures

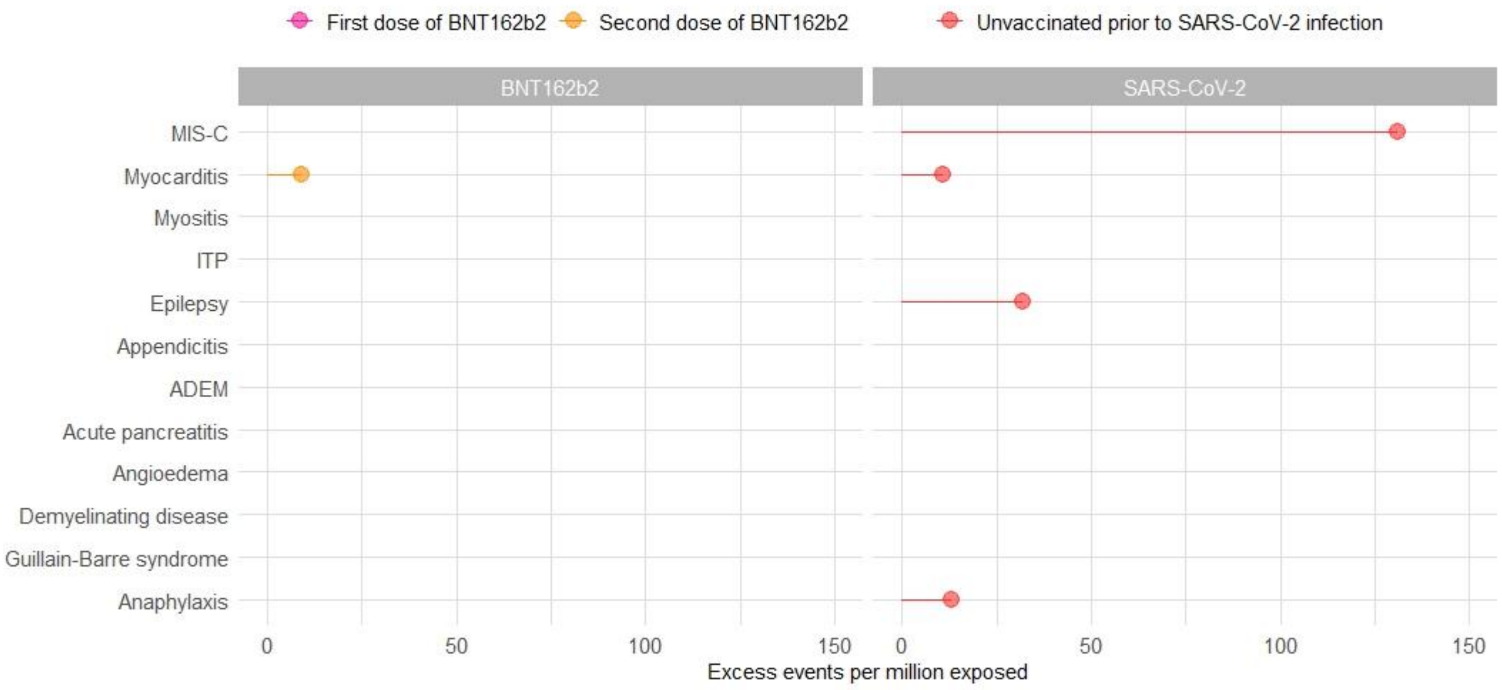

Supplementary Figure 1. Risk of serious outcomes following a first or second dose of COVID-19 vaccination with the BNT162b2 vaccine or SARS-CoV-2 infection (prior to vaccination) in male adolescents aged 12-17 years.

Estimated number of excess events per million (95% CI) based on incidence rate ratios of each outcome in the 1-42 days following vaccination or SARS-CoV-2 positive test compared to baseline period are presented where there were at least five events during the exposure period and when number of excess events is greater than zero. Data available from 8th December 2020 and 7th August 2022. MIS-C = multisystem inflammatory syndrome; ITP = idiopathic or immune thrombocytopenic purpura; ADEM = acute disseminated encephalomyelitis.

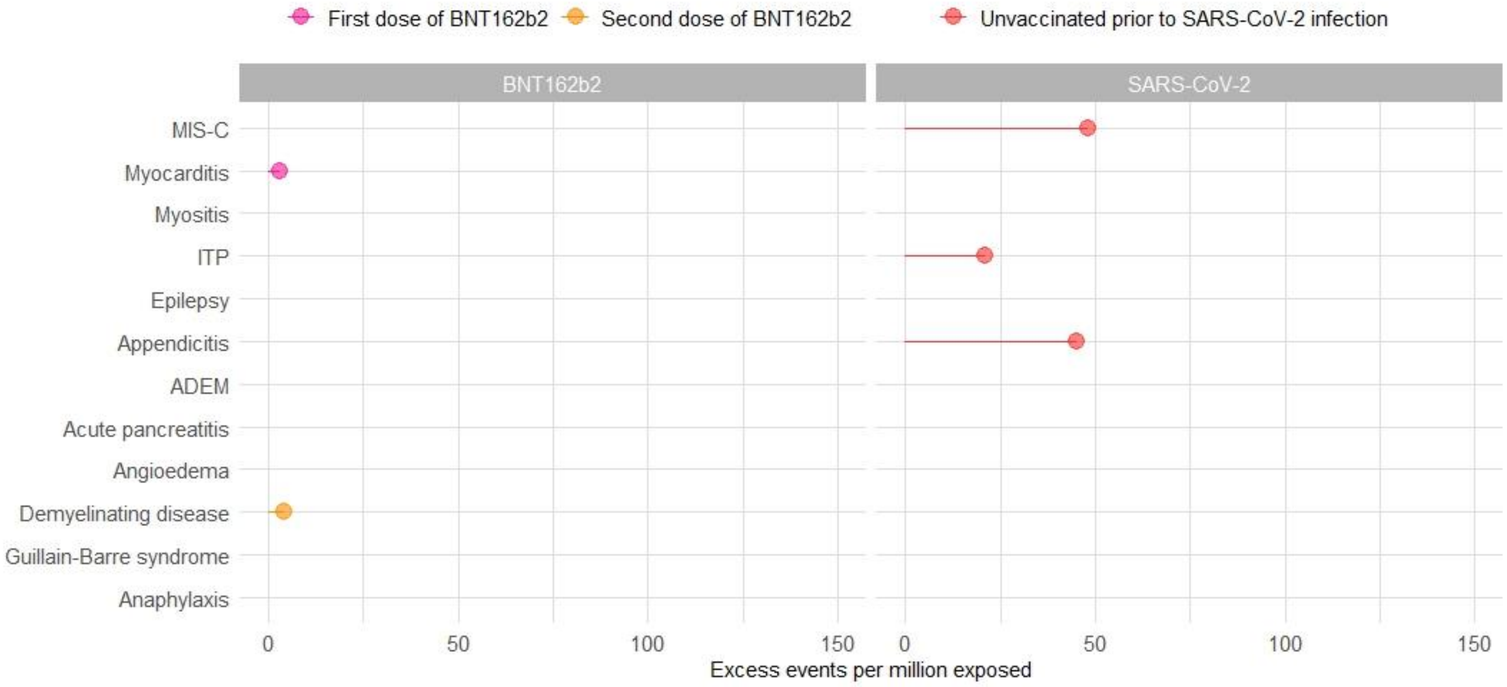

Supplementary Figure 2. Risk of serious outcomes following a first or second dose of COVID-19 vaccination with the BNT162b2 vaccine or SARS-CoV-2 infection (prior to vaccination) in female adolescents aged 12-17 years.

Estimated number of excess events per million (95% CI) based on incidence rate ratios of each outcome in the 1-42 days following vaccination or SARS-CoV-2 positive test compared to baseline period are presented where there were at least five events during the exposure period and when number of excess events is greater than zero. Data available from 8th December 2020 and 7th August 2022. MIS-C = multisystem inflammatory syndrome; ITP = idiopathic or immune thrombocytopenic purpura; ADEM = acute disseminated encephalomyelitis.

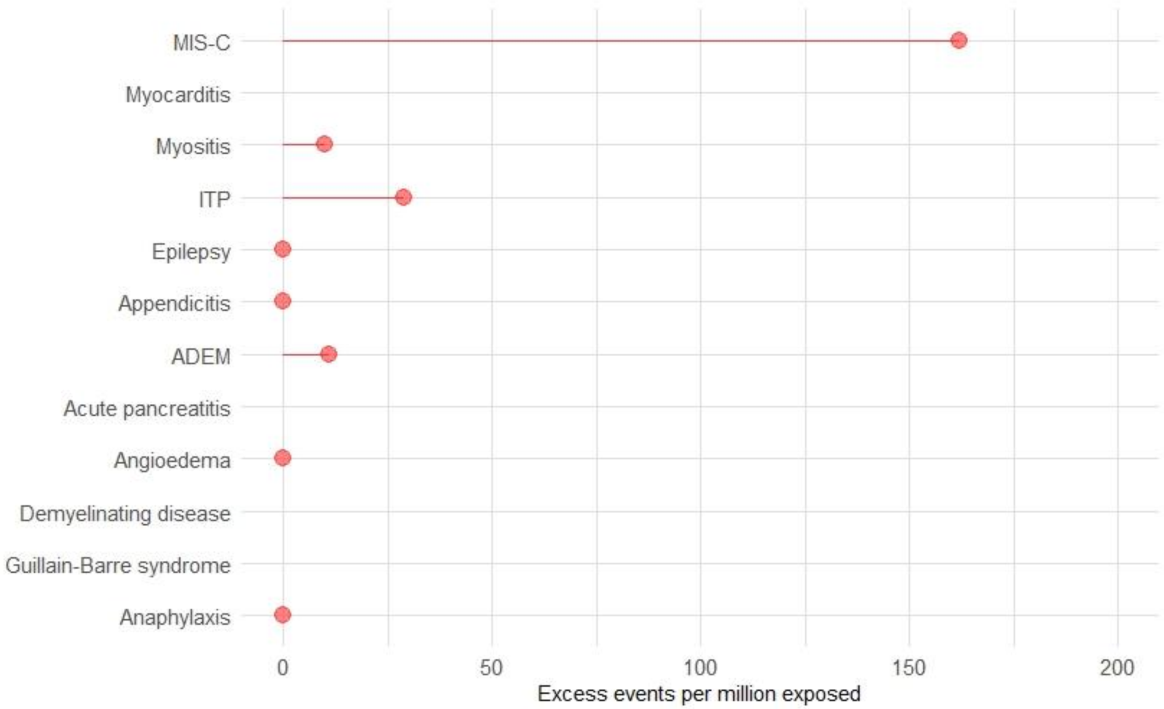

Supplementary Figure 3. Risk of serious outcomes following SARS-CoV-2 infection prior to vaccination in male children aged 5-11 years.

Estimated number of excess events per million (95% CI) based on incidence rate ratios of each outcome in the 1-42 days following SARS-CoV-2 positive test compared to baseline period are presented where there were at least five events during the exposure period and when number of excess events is greater than zero. Data available from 8th December 2020 and 7th August 2022. MIS-C = multisystem inflammatory syndrome; ITP = idiopathic or immune thrombocytopenic purpura; ADEM = acute disseminated encephalomyelitis.

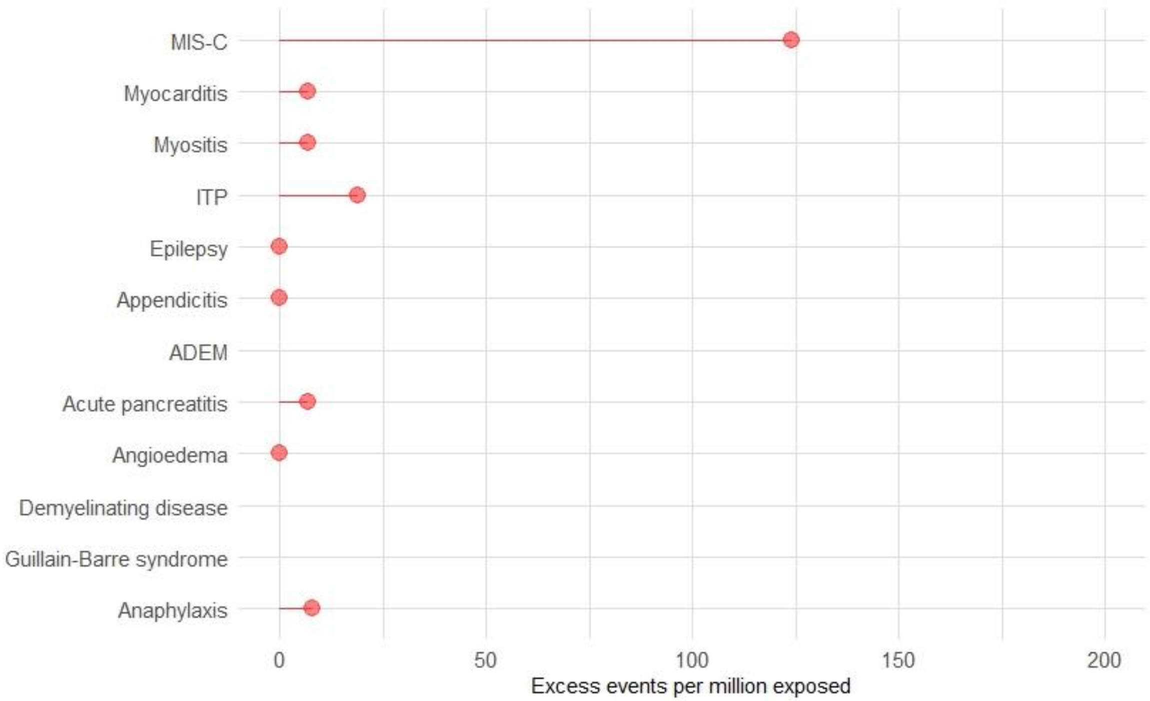

Supplementary Figure 4. Risk of serious outcomes following SARS-CoV-2 infection prior to vaccination in female children aged 5-11 years.

Estimated number of excess events per million (95% CI) based on incidence rate ratios of each outcome in the 1-42 days following SARS-CoV-2 positive test compared to baseline period are presented where there were at least five events during the exposure period and when number of excess events is greater than zero. Data available from 8th December 2020 and 7th August 2022. MIS-C = multisystem inflammatory syndrome; ITP = idiopathic or immune thrombocytopenic purpura; ADEM = acute disseminated encephalomyelitis.

Supplementary Tables

Supplementary Table 1. Characteristics of young people aged 5-11 years, 12-17 years and 18-24 years receiving either ChAdOx1, BNT162b2 or mRNA-1273 vaccines or testing positive for SARS-CoV-2 in England between 8th December 2020 and 7th August 2022. Cells with <5 are suppressed. Data are presented as column % (counts).

	Dose 1			Dose 2			Dose 3			Positive SARS-CoV-2 test
	**ChAdOX1**	**BNT162b2**	**mRNA-1273**	**ChAdOX1**	**BNT162b2**	**mRNA-1273**	**ChAdOX1**	**BNT162b2**	**mRNA-1273**	
**5-11-year-olds**	**N = 581,545**			**N = 303,118**			**N = 870**			
**Total N (row %)**	97 (<0.1)	581,356 (>99.9)	92 (<0.1)	13 (<0.1)	303,045 (>99.9)	60 (<0.1)	0 (0)	870 (100)	0 (0)	1,508,661
**Sex, % (N)**										
Female	45.4 (44)	45.3 (263,470)	41.3 (38)	53.8 (7)	45.5 (138,018)	36.7 (22)	-	42.5 (370)	-	47.5 (716,227)
Male	45.4 (44)	47.9 (278,587)	47.8 (44)	<5	48.3 (146,372)	56.7 (34)	-	56.8 (494)	-	48.6 (733,779)
Not recorded	9.3 (9)	6.8 (39,299)	10.9 (10)	<5	6.2 (18,655)	<5	-	0.7 (6)	-	3.9 (58,655)
**Mean age (SD)**	9.0 (1.7)	8.7 (2.0)	8.4 (2.1)	9.3 (1.6)	8.8 (2.0)	9.5 (1.6)	-	9.9 (1.8)	-	8.4 (1.9)
**Positive SARS-CoV-2 test during study period, % (N)**										
None	55.7 (54)	57.3 (333,392)	56.5 (52)	69.2 (9)	55.8 (169,083)	46.7 (28)	-	50.5 (439)	-	-
Prior to first vaccine dose	33.0 (32)	41.7 (242,311)	-	<5	43.2 (131,001)	-	-	40.8 (355)	-	99.6 (1,502,996)
After first vaccine dose	11.3 (11)	1.0 (5653)	<5	<5	1.0 (2961)	<5	-	8.7 (76)	-	0.4 (5665)
**Vaccine status, % (N)**										
No vaccine	-	-	-	-	-	-	-	-		83.6 (1,260,614)
Dose 1	100 (97)	100 (581,356)	100 (92)	100 (13)	100 (303,045)	100 (60)	-	100 (870)		16.4 (248047)
Dose 2	13.4 (13)	52.1 (303,085)	21.7 (20)	100 (13)	100 (303,045)	100 (60)	-	100 (870)		8.9 (133998)
Dose 3	<5	0.1 (866)		<5	0.3 (865)	<5	-	100 (870)		<0.1 (431)
**Medical history***										
Guillain-Barre syndrome	0	(<5)	0	0	(<5)	0	0	0	0	<0.1 (10)
Appendicitis	(<5)	0.1 (697)	0	(<5)	0.1 (391)	0	0	(<5)	0	0.1 (1929)
Demyelinating disease	0	<0.1 (28)	0	0	<0.1 (20)	0	0	(<5)	0	<0.1 (66)
Myositis	0	<0.1 (40)	0	0	<0.1 (22)	0	0	(<5)	0	<0.1 (86)
Myocarditis	0	<0.1 (5)	0	0	(<5)	0	0	(<5)	0	(<5)
Acute pancreatitis	(<5)	<0.1 (19)	0	0	<0.1 (13)	0	0	0	0	<0.1 (46)
Immune thrombocytopenia	0	<0.1 (237)	0	0	<0.1 (138)	0	0	1.3 (11)	0	<0.1 (571)
Multisystem inflammatory syndrome	0	<0.1 (53)	0	0	<0.1 (27)	0	0	(<5)	0	<0.1 (120)
Anaphylaxis	0	<0.1 (88)	0	0	<0.1 (47)	0	0	0	0	<0.1 (269)
Epilepsy	5.2 (5)	0.4 (2057)	0	(<5)	0.4 (1343)	0	0	3.2 (28)	0	0.2 (3038)
Angioedema	0	<0.1 (96)	0	0	<0.1 (46)	0	0	0	0	<0.1 (241)

Acute disseminated encephalomyelitis	0	<0.1 (53)	0	0	<0.1 (36)	0	0	(<5)	0	<0.1 (118)
**12-17-year-olds**	**N = 2,882,229**			**N = 2,254,214**			**N = 454,868**			
**Total N (row %)**	10,245 (0.4)	2,870,403 (99.6)	1581 (<0.1)	9190 (0.4)	2,240,546 (99.4)	4478 (0.2)	46 (<0.1)	399,845 (87.9)	54,977 (12.1)	1,602,710
**Sex, % (N)**										
Female	53.5 (5483)	47.0 (1,350,157)	44.4 (702)	54.1 (4971)	47.7 (1,067,675)	48.4 (2169)	65.2 (30)	50.1 (200,221)	52.3 (28,733)	49.5 (793,332)
Male	44.7 (4584)	47.1 (1,350,675)	43.8 (693)	44.5 (4093)	47.0 (1,051,938)	43.2 (1936)	-	46.0 (184,026)	43.1 (23,716)	45.8 (733,890)
Not recorded	1.7 (178)	5.9 (169,571)	11.8 (186)	1.4 (126)	5.4 (120,933)	8.3 (373)	<5	3.9 (15,598)	4.6 (2528)	4.7 (75,488)
**Mean age (SD)**	16.6 (0.7)	14.5 (1.8)	15.6 (1.7)	16.6 (0.6)	14.6 (1.8)	16.5 (1.1)	16.4 (0.8)	16.0 (1.1)	17.0 (0.3)	14.3 (1.7)
**Positive SARS-CoV-2 test during study period, % (N)**										
None	63.0 (6456)	60.8 (1,745,591)	63.8 (1009)	62.6 (5756)	61.3 (1,373,622)	60.5 (2708)	69.6 (32)	65.4 (261,447)	61.9 (34,039)	-
Prior to first vaccine dose	4.5 (456)	20.8 (597,636)	24.4 (385)	4.3 (395)	20.2 (452,711)	20.7 (928)	<5	12.1 (48,486)	11.4 (6278)	66.9 (1,072,014)
After first vaccine dose	32.5 (3333)	18.4 (527,176)	11.8 (187)	33.1 (3039)	18.5 (414,213)	18.8 (842)	-	22.5 (89,912)	26.7 (14,660)	33.1 (530,696)
**Vaccine status, % (N)**										
No vaccine	-	-	-	-	-	-	-	-	-	29.5 (473,537)
Dose 1	100 (10,245)	100 (2,870,403)	100 (1581)	100 (9190)	100 (2,240,546)	100 (4478)	100 (46)	100 (399,845)	100 (54,977)	70.5 (1,129,173)
Dose 2	95.7 (9801)	78.2 (2,243,498)	57.9 (915)	100 (9190)	100 (2,240,546)	100 (4478)	100 (46)	100 (399,845)	100 (54,977)	54.4 (872,128)
Dose 3	70.3 (7198)	15.6 (447,431)	15.1 (239)	75.5 (6936)	20.0 (447,329)	13.5 (603)	100 (46)	100 (399,845)	100 (54,977)	9.9 (159,350)
**Medical history***										
Guillain-Barre syndrome	(<5)	<0.1 (34)	0	(<5)	<0.1 (31)	0	0	<0.1 (5)	0	<0.1 (25)
Appendicitis	0.3 (27)	0.2 (7000)	(<5)	0.3 (23)	0.2 (5550)	0.2 (7)	(<5)	0.3 (1037)	0.2 (125)	0.3 (4141)
Demyelinating disease	(<5)	<0.1 (158)	0	(<5)	<0.1 (125)	0	0	<0.1 (55)	<0.1 (5)	<0.1 (82)
Myositis	(<5)	<0.1 (141)	0	(<5)	<0.1 (114)	0	0	<0.1 (37)	(<5)	<0.1 (68)
Myocarditis	(<5)	<0.1 (34)	0	(<5)	<0.1 (31)	0	0	<0.1 (16)	(<5)	<0.1 (15)
Acute pancreatitis	<0.1 (5)	<0.1 (215)	0	0.1 (5)	<0.1 (173)	0	0	<0.1 (68)	(<5)	<0.1 (121)
Immune thrombocytopenia	0.4 (36)	<0.1 (689)	0	0.3 (30)	<0.1 (578)	(<5)	0	0.1 (290)	<0.1 (21)	<0.1 (443)
Multisystem inflammatory syndrome	0	<0.1 (76)	0	0	<0.1 (60)	0	0	<0.1 (15)	(<5)	<0.1 (36)
Anaphylaxis	0.1 (8)	<0.1 (469)	(<5)	0.1 (8)	<0.1 (374)	(<5)	0	<0.1 (108)	<0.1 (15)	<0.1 (314)
Epilepsy	2.7 (275)	0.2 (5462)	0.6 (9)	2.7 (252)	0.2 (4576)	0.2 (8)	(<5)	0.6 (2408)	0.2 (135)	0.2 (2644)
Angioedema	0.1 (7)	<0.1 (361)	0	0.1 (7)	<0.1 (301)	(<5)	(<5)	<0.1 (85)	<0.1 (10)	<0.1 (239)
Acute disseminated encephalomyelitis	(<5)	<0.1 (156)	0	(<5)	<0.1 (133)	0	0	<0.1 (47)	<0.1 (6)	<0.1 (80)
**18-24-year-olds**	**N = 4,032,245**			**N = 3,653,947**			**N = 2,242,065**			
**Total N (row %)**	545,345 (13.5)	3,131,742 (77.7)	355,158 (8.8)	520,252 (14.2)	2,823,984 (77.2)	309,711 (8.5)	2006 (<0.1)	1,502,039 (67.0)	738,020 (32.9)	1,551,955
**Sex, % (N)**										
Female	54.8 (298,600)	41.7 (1,305,033)	36.4 (129,164)	54.8 (285,157)	43.2 (1,219,114)	38.8 (120,213)	50.2 (1007)	48.7 (731,211)	45.8 (338,072)	48.8 (757,840)
Male	37.7 (205,505)	40.1 (1,256,385)	41.6 (147,710)	37.6 (195,831)	40.7 (1,149,488)	43.4 (134,271)	30.4 (609)	38.8 (582,712)	41.4 (305,371)	37.5 (582,258)

Not recorded	7.6 (41,240)	18.2 (570,324)	22.0 (78,284)	7.5 (39,264)	16.1 (455,382)	17.8 (55,227)	19.4 (390)	12.5 (188,116)	12.8 (94,577)	13.7 (211,857)
**Mean age (SD)**	21.3 (2.0)	21.0 (2.0)	21.0 (2.0)	21.3 (2.0)	21.0 (2.0)	21.0 (2.0)	21.5 (1.9)	21.1 (2.0)	21.0 (2.0)	21.0 (2.0)
**Positive SARS-CoV-2 test during study period, % (N)**										
None	65.2 (355,451)	69.8 (2,187,453)	71.2 (252,922)	65.0 (338,095)	69.3 (1,955,645)	70.2 (217,315)	66.4 (1332)	68.7 (1,032,501)	69.0 (508,868)	
Prior to first vaccine dose	6.2 (33,649)	8.4 (262,326)	10.3 (36,441)	6.2 (32,117)	7.9 (223,345)	9.8 (30,346)	5.2 (105)	5.9 (88,739)	6.6 (48,350)	41.8 (647,952)
After first vaccine dose	28.7 (156,245)	21.8 (681,963)	18.5 (65,795)	28.8 (150,040)	22.8 (644,994)	20.0 (62,050)	28.4 (569)	25.4 (380,799)	24.5 (180,802)	58.2 (904,003)
**Vaccine status, % (N)**										
No vaccine	-	-	-	-	-	-	-	-	-	20.3 (315,536)
Dose 1	100 (545,345)	100 (3,131,742)	100 (355,158)	100 (520,252)	100 (2,823,984)	100 (309,711)	100 (2006)	100 (1,502,039)	100 (738,020)	79.7 (1,236,419)
Dose 2	97.0 (529,053)	90.2 (2,825,234)	84.4 (299,660)	100 (520,252)	100 (2,823,984)	100 (309,711)	>99.9 (2005)	>99.9 (1,501,896)	>99.9 (737,902)	73.6 (1,142,892)
Dose 3	70.8 (385,849)	54.0 (1,691,598)	46.4 (164,618)	73.8 (384,039)	59.9 (1,692,725)	53.3 (165,039)	100 (2006)	100 (1,502,039)	100 (738,020)	45.1 (699,364)
**Medical history***										
Guillain-Barre syndrome	<0.1 (43)	<0.1 (51)	<0.1 (6)	<0.1 (36)	<0.1 (49)	<0.1 (7)	0	<0.1 (44)	<0.1 (15)	<0.1 (53)
Appendicitis	0.3 (1436)	0.2 (6749)	0.2 (727)	0.3 (1365)	0.2 (6250)	0.2 (680)	0.4 (8)	0.2 (3543)	0.2 (1752)	0.2 (3780)
Demyelinating disease	<0.1 (151)	<0.1 (154)	<0.1 (11)	<0.1 (139)	<0.1 (148)	<0.1 (10)	0	<0.1 (149)	<0.1 (55)	<0.1 (116)
Myositis	<0.1 (52)	<0.1 (70)	<0.1 (5)	<0.1 (48)	<0.1 (65)	<0.1 (8)	0	<0.1 (65)	<0.1 (13)	<0.1 (59)
Myocarditis	<0.1 (102)	<0.1 (215)	<0.1 (22)	<0.1 (96)	<0.1 (202)	<0.1 (19)	(<5)	<0.1 (139)	<0.1 (53)	<0.1 (129)
Acute pancreatitis	0.1 (371)	<0.1 (753)	<0.1 (63)	0.1 (339)	<0.1 (697)	<0.1 (56)	(<5)	<0.1 (471)	<0.1 (195)	<0.1 (498)
Immune thrombocytopenia	0.1 (498)	<0.1 (712)	<0.1 (56)	0.1 (446)	<0.1 (670)	<0.1 (49)	0.3 (6)	<0.1 (579)	<0.1 (156)	<0.1 (530)
Multisystem inflammatory syndrome	<0.1 (14)	<0.1 (34)	0	<0.1 (12)	<0.1 (34)	0	0	<0.1 (27)	<0.1 (6)	<0.1 (23)
Anaphylaxis	<0.1 (253)	<0.1 (486)	<0.1 (60)	<0.1 (233)	<0.1 (446)	<0.1 (53)	0.3 (7)	<0.1 (334)	<0.1 (128)	<0.1 (365)
Epilepsy	0.9 (5129)	0.1 (3266)	<0.1 (164)	0.9 (4838)	0.1 (3020)	<0.1 (137)	1.5 (31)	0.3 (4402)	0.2 (1377)	0.2 (3072)
Angioedema	<0.1 (221)	<0.1 (470)	<0.1 (42)	<0.1 (211)	<0.1 (428)	<0.1 (37)	0.4 (8)	<0.1 (284)	<0.1 (132)	<0.1 (371)
Acute disseminated encephalomyelitis	<0.1 (84)	<0.1 (83)	<0.1 (6)	<0.1 (74)	<0.1 (76)	<0.1 (5)	0	<0.1 (93)	<0.1 (20)	<0.1 (61)

*Defined as hospital admission with ICD-10 code for condition in two years prior to study start date.

Supplementary Table 2. Baseline demographic characteristics of cohort in young people who did not receive any dose of COVID-19 vaccine and did not have a positive SARS-CoV-2 test recorded in England between 8th December 2020 and 7th August 2022, stratified by age group: 5-11, 12-17 and 18-24 years. Cells with <5 are suppressed. Data are presented as column % (counts).

	5-11 years	12-17 years	18-24 years
**Total N**	248,047	1,129,173	1,236,419
**Age, mean (SD)**	9.1 (1.8)	14.4 (1.7)	20.9 (1.9)
**Sex, % (N)**			
Female	47.1 (116,710)	50.4 (568,925)	51.0 (631,011)
Male	48.7 (120,895)	45.6 (514,362)	36.9 (455,687)
Not recorded	4.2 (10,442)	4.1 (45,886)	12.1 (149,721)
**Ethnicity, % (N)**			
White	76.5 (189,670)	75.0 (846,444)	59.8 (739,281)
Indian	4.1 (10,120)	2.5 (27,704)	1.6 (19,573)
Pakistani	0.9 (2230)	1.5 (17,080)	1.6 (19,257)
Bangladeshi	0.4 (1101)	0.6 (6459)	0.6 (7670)
Other Asian	1.6 (3920)	1.3 (14,700)	0.9 (11,716)
Black Caribbean	0.1 (248)	0.2 (2689)	0.4 (5059)
Black African	0.6 (1565)	0.9 (10,410)	0.9 (11,157)
Chinese	0.6 (1577)	0.4 (4107)	0.2 (2701)
Other	4.2 (10,483)	3.4 (38,921)	2.8 (34,942)
Not recorded	10.9 (27,133)	14.2 (160,659)	31.1 (385,063)
**Medical history in two years prior to study start date, % (N)**			
Guillain-Barre syndrome	(<5)	<0.1 (16)	<0.1 (43)
Appendicitis	0.1 (348)	0.3 (3008)	0.3 (3127)
Demyelinating disease	<0.1 (14)	<0.1 (55)	<0.1 (98)
Myositis	<0.1 (13)	<0.1 (45)	<0.1 (52)
Myocarditis	(<5)	<0.1 (11)	<0.1 (104)
Acute pancreatitis	<0.1 (6)	<0.1 (85)	<0.1 (410)
Idiopathic or immune thrombocytopenia	<0.1 (109)	<0.1 (297)	<0.1 (422)
Multisystem inflammatory syndrome	<0.1 (15)	<0.1 (27)	<0.1 (17)
Anaphylaxis	<0.1 (37)	<0.1 (213)	<0.1 (283)
Epilepsy	0.3 (779)	0.2 (1922)	0.2 (2533)
Angioedema	<0.1 (34)	<0.1 9160)	<0.1 (284)
Acute disseminated encephalomyelitis	<0.1 (31)	<0.1 (56)	<0.1 (46)

Supplementary Table 3. Number of patients who were admitted to hospital or died from each outcome in the baseline period or the 1-42 days following vaccination or SARS-CoV-2 positive test. Cells with <5 are suppressed. Data are presented as counts (column %).

	Positive SARS-CoV-2 test			COVID-19 vaccines								
	Baseline	Before vaccination	After vaccination	Baseline	BNT162b2 vaccine			mRNA-1273 vaccine			ChAdOX1 vaccine	
					Dose 1	Dose 2	Dose 3	Dose 1	Dose 2	Dose 3	Dose 1	Dose 2
**Multisystem inflammatory syndrome**												
**5-11 years**												
Total number of patients hospitalised	163	226	0	138	<5	0	0	0	0	0	0	0
Recorded in death certificate	0	0	0	0	0	0	0	0	0	0	0	0
Death within 28 days of hospital admission	0	0	0	0	0	0	0	0	0	0	0	0
**12-17 years**												
Number of patients hospitalised	78	98	<5	208	21	<5	<5	0	0	0	0	0
Recorded in death certificate	0	0	0	0	0	0	0	0	0	0	0	0
Death within 28 days of hospital admission	0	0	0	0	0	0	0	0	0	0	0	0
**18-24 years**												
Number of patients hospitalised	23	5	<5	39	<5	<5	0	0	0	0	<5	<5
Recorded in death certificate	0	0	0	0	0	0	0	0	0	0	0	0
Death within 28 days of hospital admission	0	0	0	0	0	0	0	0	0	0	0	0
**Myocarditis**												
**5-11 years**												
Number of patients hospitalised	5	6	0	<5	0	0	0	0	0	0	0	0
Recorded in death certificate	0	<5	0	0	0	0	0	0	0	0	0	0
Death within 28 days of hospital admission	0	0	0	0	0	0	0	0	0	0	0	0
**12-17 years**												
Number of patients hospitalised	62	7	<5	80	19	17	<5	0	0	<5	<5	0
Recorded in death certificate	0	0	0	0	0	0	0	0	0	0	0	0
Death within 28 days of hospital admission	0	0	0	0	0	0	0	0	0	0	0	0
**18-24 years**												
Number of patients hospitalised	189	19	<5	318	26	49	27	9	29	13	5	9
Recorded in death certificate	<5	0	0	<5	0	0	<5	0	0	0	0	0
Death within 28 days of hospital admission	0	0	0	0	0	0	0	0	0	0	0	0
**Idiopathic or immune thrombocytopenia**												
**5-11 years**												
Number of patients hospitalised	360	61	0	151	10	<5	0	0	0	0	0	0
Recorded in death certificate	0	0	0	0	0	0	0	0	0	0	0	0
Death within 28 days of hospital admission	0	0	0	0	0	0	0	0	0	0	0	0
**12-17 years**												
Number of patients hospitalised	399	37	6	571	41	31	10	0	0	<5	<5	<5

Recorded in death certificate	0	0	0	0	0	0	0	0	0	0	0	0
Death within 28 days of hospital admission	<5	0	0	<5	0	0	0	0	0	0	0	0
**18-24 years**												
Number of patients hospitalised	502	58	15	963	58	55	32	<5	6	12	40	29
Recorded in death certificate	0	0	0	0	0	0	0	0	0	0	0	0
Death within 28 days of hospital admission	0	0	0	0	0	0	0	0	0	0	0	0
**Epilepsy**												
**5-11 years**												
Number of patients hospitalised	1275	100	<5	780	61	27	0	0	0	0	0	0
Recorded in death certificate	0	0	0	0	0	0	0	0	0	0	0	0
Death within 28 days of hospital admission	0	0	0	0	0	0	0	0	0	0	0	0
**12-17 years**												
Number of patients hospitalised	1375	77	22	2337	215	190	48	0	<5	6	15	9
Recorded in death certificate	0	0	0	0	0	0	0	0	0	0	0	0
Death within 28 days of hospital admission	0	0	0	0	0	0	0	0	0	0	0	0
**18-24 years**												
Number of patients hospitalised	1863	83	42	3946	168	156	172	7	6	40	215	210
Recorded in death certificate	0	0	0	0	0	0	0	0	0	0	0	0
Death within 28 days of hospital admission	0	0	0	0	0	0	0	0	0	0	0	0
**Acute pancreatitis**												
**5-11 years**												
Number of patients hospitalised	38	9	0	17	<5	0	0	0	0	0	0	0
Recorded in death certificate	0	0	0	0	0	0	0	0	0	0	0	0
Death within 28 days of hospital admission	0	0	0	0	0	0	0	0	0	0	0	0
**12-17 years**												
Number of patients hospitalised	142	7	<5	207	28	10	5	0	0	0	<5	0
Recorded in death certificate	0	0	0	0	0	0	0	0	0	0	0	0
Death within 28 days of hospital admission	0	0	0	0	0	0	0	0	0	0	0	0
**18-24 years**												
Number of patients hospitalised	495	30	6	934	70	63	39	8	5	13	10	26
Recorded in death certificate	0	0	0	0	0	0	0	0	0	0	0	0
Death within 28 days of hospital admission	0	0	0	0	0	0	0	0	0	0	0	0
**Myositis**												
**5-11 years**												
Number of patients hospitalised	51	15	0	21	<5	0	0	0	0	0	0	0
Recorded in death certificate	0	0	0	0	0	0	0	0	0	0	0	0
Death within 28 days of hospital admission	0	0	0	0	0	0	0	0	0	0	0	0
**12-17 years**												
Number of patients hospitalised	48	<5	0	70	7	<5	<5	0	0	0	0	<5

Recorded in death certificate	0	0	0	0	0	0	0	0	0	0	0	0
Death within 28 days of hospital admission	0	0	0	0	0	0	0	0	0	0	0	0
**18-24 years**												
Number of patients hospitalised	27	<5	<5	53	<5	<5	<5	0	<5	0	<5	<5
Recorded in death certificate	0	0	0	0	0	0	0	0	0	0	0	0
Death within 28 days of hospital admission	0	0	0	0	0	0	0	0	0	0	0	0
**Acute disseminated encephalomyelitis**												
**5-11 years**												
Number of patients hospitalised	61	12	0	27	<5	0	0	0	0	0	0	0
Recorded in death certificate	0	0	0	0	0	0	0	0	0	0	0	0
Death within 28 days of hospital admission	0	0	0	0	0	0	0	0	0	0	0	0
**12-17 years**												
Number of patients hospitalised	75	6	<5	94	5	8	0	0	0	0	0	0
Recorded in death certificate	0	0	0	0	0	0	0	0	0	0	0	0
Death within 28 days of hospital admission	0	0	0	0	0	0	0	0	0	0	0	0
**18-24 years**												
Number of patients hospitalised	69	<5	<5	124	12	8	5	<5	<5	<5	<5	6
Recorded in death certificate	0	0	0	0	0	0	0	0	0	0	0	0
Death within 28 days of hospital admission	0	0	0	0	0	0	0	0	0	0	0	0
**Demyelinating disease**												
**5-11 years**												
Number of patients hospitalised	46	6	0	16	<5	<5	0	0	0	0	0	0
Recorded in death certificate	0	0	0	0	0	0	0	0	0	0	0	0
Death within 28 days of hospital admission	0	0	0	0	0	0	0	0	0	0	0	0
**12-17 years**												
Number of patients hospitalised	177	<5	<5	110	7	9	<5	0	0	0	0	0
Recorded in death certificate	0	0	0	0	0	0	0	0	0	0	0	0
Death within 28 days of hospital admission	0	0	0	0	0	0	0	0	0	0	0	0
**18-24 years**												
Number of patients hospitalised	130	<5	<5	266	11	11	11	0	<5	<5	7	5
Recorded in death certificate	0	0	0	0	0	0	0	0	0	0	0	0
Death within 28 days of hospital admission	0	0	0	0	0	0	0	0	0	0	0	0
**Anaphylaxis**												
**5-11 years**												
Number of patients hospitalised	208	24	0	70	<5	0	0	0	0	0	0	0
Recorded in death certificate	0	0	0	0	0	0	0	0	0	0	0	0
Death within 28 days of hospital admission	0	0	0	0	0	0	0	0	0	0	0	0
**12-17 years**												
Number of patients hospitalised	265	19	<5	397	44	21	7	0	0	<5	0	0

Recorded in death certificate	0	0	0	0	0	0	0	0	0	0	0	0
Death within 28 days of hospital admission	0	0	0	0	0	0	0	0	0	0	0	0
**18-24 years**												
Number of patients hospitalised	306	10	<5	476	40	30	16	<5	<5	6	19	20
Recorded in death certificate	0	0	0	0	0	0	0	0	0	0	0	0
Death within 28 days of hospital admission	0	0	0	0	0	0	0	0	0	0	0	0
**Appendicitis**												
**5-11 years**												
Number of patients hospitalised	2346	213	<5	823	63	8	0	0	0	0	0	0
Recorded in death certificate	0	0	0	0	0	0	0	0	0	0	0	0
Death within 28 days of hospital admission	0	0	0	0	0	0	0	0	0	0	0	0
**12-17 years**												
Number of patients hospitalised	3665	209	26	5512	461	330	64	0	0	9	<5	6
Recorded in death certificate	0	0	0	0	0	0	0	0	0	0	0	0
Death within 28 days of hospital admission	0	0	0	0	0	0	0	0	0	0	0	0
**18-24 years**												
Number of patients hospitalised	3123	117	57	6001	470	382	207	60	48	93	111	94
Recorded in death certificate	0	0	0	0	0	0	0	0	0	0	0	0
Death within 28 days of hospital admission	0	0	0	0	0	0	0	0	0	0	0	0
**Guillain-Barre syndrome**												
**5-11 years**												
Number of patients hospitalised	15	0	0	<2	0	0	0	0	0	0	0	0
Recorded in death certificate	0	0	0	0	0	0	0	0	0	0	0	0
Death within 28 days of hospital admission	0	0	0	0	0	0	0	0	0	0	0	0
**12-17 years**												
Number of patients hospitalised	13	5	<5	22	<5	<5	0	0	0	0	0	0
Recorded in death certificate	0	0	0	0	0	0	0	0	0	0	0	0
Death within 28 days of hospital admission	0	0	0	0	0	0	0	0	0	0	0	0
**18-24 years**												
Number of patients hospitalised	32	<5	0	58	<5	5	<5	<5	0	<5	<5	0
Recorded in death certificate	0	0	0	0	0	0	0	0	0	0	0	0
Death within 28 days of hospital admission	0	0	0	0	0	0	0	0	0	0	0	0
**Angioedema**												
**5-11 years**												
Number of patients hospitalised	173	12	0	54	<5	0	0	0	0	0	0	0
Recorded in death certificate	0	0	0	0	0	0	0	0	0	0	0	0
Death within 28 days of hospital admission	0	0	0	0	0	0	0	0	0	0	0	0
**12-17 years**												
Number of patients hospitalised	164	11	<5	224	25	12	<5	0	0	<5	<5	<5

Recorded in death certificate	0	0	0	0	0	0	0	0	0	0	0	0
Death within 28 days of hospital admission	0	0	0	0	0	0	0	0	0	0	0	0
**18-24 years**												
Number of patients hospitalised	238	7	6	390	28	25	6	<5	<5	<5	17	13
Recorded in death certificate	0	0	0	0	0	0	0	0	0	<5	0	0
Death within 28 days of hospital admission	0	0	0	0	0	0	0	0	0	0	0	0

Supplementary Table 4. Incidence rate ratios and estimated number of excess events per million for individual outcomes in the 1-42 days following vaccination or a SARS-CoV-2 positive test (before or after vaccination) compared to baseline period in females aged 5-11 and 12-17 years. Cells with a hyphen are suppressed; N – number of events; IRR – incidence rate ratio; CI – confidence interval.

	**BNT162b2 vaccine**			**mRNA-1273 vaccine**			**ChAdOX1 vaccine**			**Positive SARS-CoV-2 test**		
	N	IRR (95% CI)	Excess events (95% CI)	N	IRR (95% CI)	Excess events (95% CI)	N	IRR (95% CI)	Excess events (95% CI)	N	IRR (95% CI)	Excess events (95% CI)
**5-11 years**												
**Multisystem inflammatory syndrome**												
First dose/positive test (before vaccine)	<5	-	-	0	-	-	0	-	-	97	11.13 (7.96, 15.57)	124 (119, 127)
Second dose/positive test (after vaccine)	0	-	-	0	-	-	0	-	-	0	-	-
Third dose	0	-	-	0	-	-	0	-	-			
**Myocarditis**												
First dose/positive test (before vaccine)	0	-	-	0	-	-	0	-	-	5	18.93 (3.22, 111.28)	7 (5, 7)
Second dose/positive test (after vaccine)	0	-	-	0	-	-	0	-	-	0	-	-
Third dose	0	-	-	0	-	-	0	-	-			
**Immune or idiopathic thrombocytopenia**												
First dose/positive test (before vaccine)	6	1.26 (0.52, 3.07)	0	0	-	-	0	-	-	25	2.20 (1.42, 3.42)	19 (10, 25)
Second dose/positive test (after vaccine)	<5	-	-	0	-	-	0	-	-	0	-	-
Third dose	0	-	-	0	-	-	0	-	-			
**Epilepsy**												
First dose/positive test (before vaccine)	34	1.28 (0.87, 1.90)	0	0	-	-	0	-	-	39	0.96 (0.69, 1.35)	0
Second dose/positive test (after vaccine)	17	1.36 (0.78, 2.34)	0	0	-	-	0	-	-	0	-	-
Third dose	0	-	-	0	-	-	0	-	-			
**Acute pancreatitis**												
First dose/positive test (before vaccine)	<5	-	-	0	-	-	0	-	-	7	3.86 (1.44, 10.37)	7 (3, 9)
Second dose/positive test (after vaccine)	0	-	-	0	-	-	0	-	-	0	-	-
Third dose	0	-	-	0	-	-	0	-	-			

**Myositis**												
First dose/positive test (before vaccine)	0	-	-	0	-	-	0	-	-	7	3.37 (1.32, 8.60)	7 (2, 9)
Second dose/positive test (after vaccine)	0	-	-	0	-	-	0	-	-	0	-	-
Third dose	0	-	-	0	-	-	0	-	-			
**Acute disseminated encephalomyelitis**												
First dose/positive test (before vaccine)	<5	-	-	0	-	-	0	-	-	<5	-	-
Second dose/positive test (after vaccine)	0	-	-	0	-	-	0	-	-	0	-	-
Third dose	0	-	-	0	-	-	0	-	-			
**Demyelinating disease**												
First dose/positive test (before vaccine)	<5	-	-	0	-	-	0	-	-	<5	-	-
Second dose/positive test (after vaccine)	0	-	-	0	-	-	0	-	-	0	-	-
Third dose	0	-	-	0	-	-	0	-	-			
**Anaphylaxis**												
First dose/positive test (before vaccine)	<5	-	-	0	-	-	0	-	-	10	2.20 (1.03, 4.67)	8 (0, 11)
Second dose/positive test (after vaccine)	0	-	-	0	-	-	0	-	-	0	-	-
Third dose	0	-	-	0	-	-	0	-	-			
**Appendicitis**												
First dose/positive test (before vaccine)	19	0.94 (0.57, 1.53)	0	0	-	-	0	-	-	83	1.17 (0.92, 1.48)	0
Second dose/positive test (after vaccine)	<5	-	-	0	-	-	0	-	-	<5	-	-
Third dose	0	-	-	0	-	-	0	-	-			
**Guillain-Barre Syndrome**												
First dose/positive test (before vaccine)	0	-	-	0	-	-	0	-	-	0	-	-
Second dose/positive test (after vaccine)	0	-	-	0	-	-	0	-	-	0	-	-
Third dose	0	-	-	0	-	-	0	-	-			
**Angioedema**												
First dose/positive test (before vaccine)	0	-	-	0	-	-	0	-	-	6	1.06 (0.44, 2.57)	0

Second dose/positive test (after vaccine)	0	-	-	0	-	-	0	-	-	0	-	-
Third dose	0	-	-	0	-	-	0	-	-			
**12-17 years**												
**Multisystem inflammatory syndrome**												
First dose/positive test (before vaccine)	<5	-	-	0	-	-	0	-	-	27	13.11 (6.90, 24.91)	48 (44, 50)
Second dose/positive test (after vaccine)	<5	-	-	0	-	-	0	-	-	0	-	-
Third dose	<5	-	-	0	-	-	0	-	-			
**Myocarditis**												
First dose/positive test (before vaccine)	6	4.01 (1.33, 12.09)	3 (1, 4)	0	-	-	0	-	-	0	-	-
Second dose/positive test (after vaccine)	<5	-	-	0	-	-	0	-	-	0	-	-
Third dose	0	-	-	0	-	-	0	-	-			
**Immune or idiopathic thrombocytopenia**												
First dose/positive test (before vaccine)	20	0.60 (0.37, 0.96)	0	0	-	-	<5	-	-	22	2.00 (1.26, 3.17)	21 (9, 29)
Second dose/positive test (after vaccine)	17	0.70 (0.42, 1.18)	0	0	-	-	0	-	-	<5	-	-
Third dose	<5	-	-	0	-	-	0	-	-			
**Epilepsy**												
First dose/positive test (before vaccine)	116	1.10 (0.90, 1.35)	0	0	-	-	8	2.26 (1.03, 4.94)	813 (44, 1164)	30	1.06 (0.73, 1.54)	0
Second dose/positive test (after vaccine)	101	1.17 (0.95, 1.45)	0	<5	-	-	5	1.33 (0.51, 3.44)	0	12	1.59 (0.89, 2.84)	0
Third dose	30	0.97 (0.66, 1.43)	0	<5	-	-	0	-	-			
**Acute pancreatitis**												
First dose/positive test (before vaccine)	18	1.70 (0.99, 2.90)	0	0	-	-	<5	-	-	<5	-	-
Second dose/positive test (after vaccine)	9	1.10 (0.54, 2.25)	0	0	-	-	0	-	-	<5	-	-
Third dose	<5	-	-	0	-	-	0	-	-			
**Myositis**												
First dose/positive test (before vaccine)	6	1.75 (0.67, 4.55)	0	0	-	-	0	-	-	<5	-	-
Second dose/positive test (after vaccine)	0	-	-	0	-	-	0	-	-	0	-	-
Third dose	<5	-	-	0	-	-	0	-	-			

**Acute disseminated encephalomyelitis**												
First dose/positive test (before vaccine)	<5	-	-	0	-	-	0	-	-	<5	-	-
Second dose/positive test (after vaccine)	5	2.56 (0.92, 7.10)	0	0	-	-	0	-	-	<5	-	-
Third dose	0	-	-	0	-	-	0	-	-			
**Demyelinating disease**												
First dose/positive test (before vaccine)	5	0.89 (0.34, 2.34)	0	0	-	-	0	-	-	<5	-	-
Second dose/positive test (after vaccine)	8	2.41 (1.06, 5.48)	4 (0, 6)	0	-	-	0	-	-	<5	-	-
Third dose	0	-	-	0	-	-	0	-	-			
**Anaphylaxis**												
First dose/positive test (before vaccine)	24	1.21 (0.76, 1.92)	0	0	-	-	0	-	-	7	0.98 (0.45, 2.14)	0
Second dose/positive test (after vaccine)	9	0.68 (0.34, 1.36)	0	0	-	-	0	-	-	<5	-	-
Third dose	<5	-	-	<5	-	-	0	-	-			
**Appendicitis**												
First dose/positive test (before vaccine)	191	0.83 (0.70, 0.97)	0	0	-	-	<5	-	-	108	1.28 (1.05, 1.57)	45 (9, 75)
Second dose/positive test (after vaccine)	155	0.90 (0.75, 1.07)	0	0	-	-	<5	-	-	11	0.65 (0.36, 1.19)	0
Third dose	29	0.87 (0.59, 1.27)	0	5	1.35 (0.54, 3.42)	0	0	-	-			
**Guillain-Barre Syndrome**												
First dose/positive test (before vaccine)	0	-	-	0	-	-	0	-	-	<5	-	-
Second dose/positive test (after vaccine)	<5	-	-	0	-	-	0	-	-	0	-	-
Third dose	0	-	-	0	-	-	0	-	-			
**Angioedema**												
First dose/positive test (before vaccine)	16	1.37 (0.78, 2.41)	0	0	-	-	0	-	-	6	1.15 (0.49, 2.70)	0
Second dose/positive test (after vaccine)	8	0.82 (0.39, 1.73)	0	0	-	-	0	-	-	<5	-	-
Third dose	<5	-	-	<5	-	-	0	-	-			

Supplementary Table 5. Incidence rate ratios and estimated number of excess events per million for individual outcomes in the 1-42 days following vaccination or a SARS-CoV-2 positive test (before or after vaccination) compared to baseline period in males aged 5-11 and 12-17 years. Cells with a hyphen are suppressed; N – number of events; IRR – incidence rate ratio; CI – confidence interval.

	BNT162b2 vaccine			mRNA-1273 vaccine			ChAdOX1 vaccine			Positive SARS-CoV-2 test		
	N	IRR (95% CI)	Excess events (95% CI)	N	IRR (95% CI)	Excess events (95% CI)	N	IRR (95% CI)	Excess events (95% CI)	N	IRR (95% CI)	Excess events (95% CI)
**5-11 years**												
**Multisystem inflammatory syndrome**												
First dose/positive test (before vaccine)	0	-	-	0	-	-	0		-	129	12.00 (8.92, 16.12)	162 (157, 166)
Second dose/positive test (after vaccine)	0	-	-	0	-	-	0		-	0	-	-
Third dose	0	-	-	0	-	-	0		-			
**Myocarditis**												
First dose/positive test (before vaccine)	0	-	-	0	-	-	0	-	-	<5	-	-
Second dose/positive test (after vaccine)	0	-	-	0	-	-	0	-	-	0	-	-
Third dose	0	-	-	0	-	-	0	-	-			
**Immune or idiopathic thrombocytopenia**												
First dose/positive test (before vaccine)	<5	-	-	0	-	-	0	-	-	36	2.46 (1.68, 3.58)	29 (20, 36)
Second dose/positive test (after vaccine)	0	-	-	0	-	-	0	-	-	0	-	-
Third dose	0	-	-	0	-	-	0	-	-			
**Epilepsy**												
First dose/positive test (before vaccine)	27	0.80 (0.52, 1.21)	0	0	-	-	0	-	-	61	1.26 (0.96, 1.66)	0
Second dose/positive test (after vaccine)	10	0.59 (0.31, 1.13)	0	0	-	-	0	-	-	<5	-	-
Third dose	0	-	-	0	-	-	0	-	-			
**Acute pancreatitis**												
First dose/positive test (before vaccine)	0	-	-	0	-	-	0	-	-	<5	-	-
Second dose/positive test (after vaccine)	0	-	-	0	-	-	0	-	-	0	-	-
Third dose	0	-	-	0	-	-	0	-	-			

**Myositis**												
First dose/positive test (before vaccine)	<5	-	-	0	-	-	0	-	-	8	8.70 (3.20, 23.67)	10 (8, 10)
Second dose/positive test (after vaccine)	0	-	-	0	-	-	0	-	-	0	-	-
Third dose	0	-	-	0	-	-	0	-	-			
**Acute disseminated encephalomyelitis**												
First dose/positive test (before vaccine)	0	-	-	0	-	-	0	-	-	10	5.15 (2.27, 11.68)	11 (8, 13)
Second dose/positive test (after vaccine)	0	-	-	0	-	-	0	-	-	0	-	-
Third dose	0	-	-	0	-	-	0	-	-			
**Demyelinating disease**												
First dose/positive test (before vaccine)	0	-	-	0	-	-	0	-	-	<5	-	-
Second dose/positive test (after vaccine)	<5	-	-	0	-	-	0	-	-	0	-	-
Third dose	0	-	-	0	-	-	0	-	-			
**Anaphylaxis**												
First dose/positive test (before vaccine)	<5	-	-	0	-	-	0	-	-	14	1.17 (0.64, 2.13)	0
Second dose/positive test (after vaccine)	0	-	-	0	-	-	0	-	-	0	-	-
Third dose	0	-	-	0	-	-	0	-	-			
**Appendicitis**												
First dose/positive test (before vaccine)	44	1.06 (0.76, 1.48)	0	0	-	-	0	-	-	130	1.14 (0.94, 1.37)	0
Second dose/positive test (after vaccine)	<5	-	-	0	-	-	0	-	-	0	-	-
Third dose	0	-	-	0	-	-	0	-	-			
**Guillain-Barre Syndrome**												
First dose/positive test (before vaccine)	0	-	-	0	-	-	0	-	-	0	-	-
Second dose/positive test (after vaccine)	0	-	-	0	-	-	0	-	-	0	-	-
Third dose	0	-	-	0	-	-	0	-	-			
**Angioedema**												
First dose/positive test (before vaccine)	<5	-	-	0	-	-	0	-	-	6	0.99 (0.42, 2.36)	0

Second dose/positive test (after vaccine)	0	-	-	0	-	-	0	-	-	0	-	-
Third dose	0	-	-	0	-	-	0	-	-			
**12-17 years**												
**Multisystem inflammatory syndrome**												
First dose/positive test (before vaccine)	17	0.76 (0.38, 1.49)	0	0	-	-	0	-	-	71	12.33 (8.31, 18.31)	131 (126, 135)
Second dose/positive test (after vaccine)	<5	-	-	0	-	-	0	-	-	<5	-	-
Third dose	0	-	-	0	-	-	0	-	-			
**Myocarditis**												
First dose/positive test (before vaccine)	13	1.44 (0.71, 2.92)	0	0	-	-	<5	-	-	7	4.39 (1.74, 11.10)	11 (6, 13)
Second dose/positive test (after vaccine)	14	2.87 (1.50, 5.51)	9 (4, 11)	0	-	-	0	-	-	<5	-	-
Third dose	<5	-	-	<5	-	-	0	-	-			
**Immune or idiopathic thrombocytopenia**												
First dose/positive test (before vaccine)	21	1.00 (0.63, 1.59)	0	0	-	-	0	-	-	15	1.58 (0.91, 2.72)	0
Second dose/positive test (after vaccine)	14	0.99 (0.56, 1.73)	0	0	-	-	<5	-	-	<5	-	-
Third dose	8	1.46 (0.69, 3.09)	0	<5	-	-	0	-	-			
**Epilepsy**												
First dose/positive test (before vaccine)	99	0.94 (0.75, 1.16)	0	0	-	-	7	1.63 (0.72, 3.70)	0	47	1.50 (1.11, 2.04)	32 (9, 48)
Second dose/positive test (after vaccine)	89	1.16 (0.93, 1.46)	0	0	-	-	<5	-	-	10	1.57 (0.83, 2.96)	0
Third dose	18	0.68 (0.42, 1.10)	0	<5	-	-	0	-	-			
**Acute pancreatitis**												
First dose/positive test (before vaccine)	10	0.80 (0.39, 1.65)	0	0	-	-	<5	-	-	<5	-	-
Second dose/positive test (after vaccine)	<5	-	-	0	-	-	0	-	-	0	-	-
Third dose	<5	-	-	0	-	-	0	-	-			
**Myositis**												
First dose/positive test (before vaccine)	<5	-	-	0	-	-	0	-	-	0	-	-
Second dose/positive test (after vaccine)	<5	-	-	0	-	-	<5	-	-	0	-	-
Third dose	0	-	-	0	-	-	0	-	-			

**Acute disseminated encephalomyelitis**												
First dose/positive test (before vaccine)	<5	-	-	0	-	-	0	-	-	<5	-	-
Second dose/positive test (after vaccine)	<5	-	-	0	-	-	0	-	-	<5	-	-
Third dose	0	-	-	0	-	-	0	-	-			
**Demyelinating disease**												
First dose/positive test (before vaccine)	<5	-	-	0	-	-	0	-	-	<5	-	-
Second dose/positive test (after vaccine)	<5	-	-	0	-	-	0	-	-	0	-	-
Third dose	<5	-	-	0	-	-	0	-	-			
**Anaphylaxis**												
First dose/positive test (before vaccine)	20	1.52 (0.91, 2.52)	0	0		-	0	-	-	12	2.20 (1.18, 4.10)	13 (4, 18)
Second dose/positive test (after vaccine)	12	1.07 (0.57, 1.99)	0	0		-	0	-	-	<5	-	-
Third dose	<5	-	-	0		-	0	-	-			
**Appendicitis**												
First dose/positive test (before vaccine)	270	0.97 (0.85, 1.11)	0	0	-	-	<5	-	-	101	0.92 (0.75, 1.13)	0
Second dose/positive test (after vaccine)	175	0.89 (0.76, 1.05)	0	0	-	-	<5	-	-	15	0.95 (0.57, 1.59)	0
Third dose	35	1.22 (0.86, 1.73)	0	<5	-	-	0	-	-			
**Guillain-Barre Syndrome**												
First dose/positive test (before vaccine)	<5	-	-	0	-	-	0	-	-	<5	-	-
Second dose/positive test (after vaccine)	0	-	-	0	-	-	0	-	-	<5	-	-
Third dose	0	-	-	0	-	-	0	-	-			
**Angioedema**												
First dose/positive test (before vaccine)	9	1.46 (0.68, 3.17)	0	0	-	-	<5	-	-	5	2.08 (0.77, 5.60)	0
Second dose/positive test (after vaccine)	<5	-	-	0	-	-	<5	-	-	0	-	-
Third dose	<5	-	-	0	-	-	0	-	-			

Supplementary Table 6. Risks of individual outcomes in the 1-42 days following vaccination or a SARS-CoV-2 positive test (before or after vaccination) compared to baseline period in young people aged 5-11, 12-17 and 18-24 years of white and non-white ethnicity. Cells with a hyphen are suppressed and cells with * are models that did not converge; N – number of events; IRR – incidence rate ratio; CI – confidence interval. Relative IRR ~ (IRR in young people of non-white ethnicity)/(IRR in young people of white ethnicity). Non-white ethnicity comprises Indian, Pakistani, Bangladeshi, Other Asian, Black Caribbean, Black African, Chinese and Other ethnicity as recorded on hospital or GP record.

	BNT162b2 vaccine			mRNA-1273 vaccine			ChAdOX1 vaccine			Positive SARS-CoV-2 test		
	N	IRR	Relative IRR (95% CI)	N	IRR	Relative IRR (95% CI)	N	IRR	Relative IRR (95% CI)	N	IRR	Relative IRR (95% CI)
**5-11 years**												
**Multisystem inflammatory syndrome**												
White ethnicity												
First dose/positive test (before vaccine)	<5	-	-	0	-	-	0	-	-	147	11.24 (8.46, 14.94)	1 (Ref)
Second dose/positive test (after vaccine)	0	-	-	0	-	-	0	-	-	0	-	-
Third dose	0	-	-	0	-	-	0	-	-			
Non-white ethnicity												
First dose/positive test (before vaccine)	0	-	-	0	-	-	0	-	-	55	11.91 (7.78, 18.24)	0.98 (0.60, 1.60)
Second dose/positive test (after vaccine)	0	-	-	0	-	-	0	-	-	0	-	-
Third dose	0	-	-	0	-	-	0	-	-			
**Myocarditis**												
White ethnicity												
First dose/positive test (before vaccine)	0	-	-	0	-	-	0	-	-	6	35.89 (2.10, 613.53)	1 (Ref)
Second dose/positive test (after vaccine)	0	-	-	0	-	-	0	-	-	0	-	-
Third dose	0	-	-	0	-	-	0	-	-			
Non-white ethnicity												
First dose/positive test (before vaccine)	0	-	-	0	-	-	0	-	-	0	-	-
Second dose/positive test (after vaccine)	0	-	-	0	-	-	0	-	-	0	-	-
Third dose	0	-	-	0	-	-	0	-	-			
**Immune or idiopathic thrombocytopenia**												
White ethnicity												
First dose/positive test (before vaccine)	9	1.04 (0.48, 2.22)	1 (Ref)	0	-	-	0	-	-	36	1.94 (1.35, 2.80)	1 (Ref)

Second dose/positive test (after vaccine)	<5	-	-	0	-	-	0	-	-	0	-	-
Third dose	0	-	-	0	-	-	0	-	-			
Non-white ethnicity												
First dose/positive test (before vaccine)	<5	-	-	0	-	-	0	-	-	21	3.33 (2.01, 5.54)	1.79 (0.98, 3.25)
Second dose/positive test (after vaccine)	0	-	-	0	-	-	0	-	-	0	-	-
Third dose	0	-	-	0	-	-	0	-	-			
**Epilepsy**												
White ethnicity												
First dose/positive test (before vaccine)	49	*	*	0	-	-	0	-	-	72	1.00 (0.78, 1.28)	*
Second dose/positive test (after vaccine)	26	1.15 (0.75, 1.77)	1 (Ref)	0	-	-	0	-	-	0	-	-
Third dose	0	-	-	0	-	-	0	-	-			
Non-white ethnicity												
First dose/positive test (before vaccine)	11	1.04 (0.52, 2.07)	*	0	-	-	0	-	-	19	1.47 (0.90, 2.41)	*
Second dose/positive test (after vaccine)	0	-	-	0	-	-	0	-	-	<5	-	-
Third dose	0	-	-	0	-	-	0	-	-			
**Acute pancreatitis**												
White ethnicity												
First dose/positive test (before vaccine)	0	-	-	0	-	-	0	-	-	5	2.21 (0.75, 6.51)	1 (Ref)
Second dose/positive test (after vaccine)	0	-	-	0	-	-	0	-	-	0	-	-
Third dose	0	-	-	0	-	-	0	-	-			
Non-white ethnicity												
First dose/positive test (before vaccine)	<5	-	-	0	-	-	0	-	-	<5	-	-
Second dose/positive test (after vaccine)	0	-	-	0	-	-	0	-	-	0	-	-
Third dose	0	-	-	0	-	-	0	-	-			
**Myositis**												
White ethnicity												
First dose/positive test (before vaccine)	<5	-	-	0	-	-	0	-	-	13	6.87 (3.19, 14.77)	1 (Ref)
Second dose/positive test (after vaccine)	0	-	-	0	-	-	0	-	-	0	-	-
Third dose	0	-	-	0	-	-	0	-	-			

Non-white ethnicity												
First dose/positive test (before vaccine)	0	-	-	0	-	-	0	-	-	<5	-	-
Second dose/positive test (after vaccine)	0	-	-	0	-	-	0	-	-	0	-	-
Third dose	0	-	-	0	-	-	0	-	-			
**Acute disseminated encephalomyelitis**												
White ethnicity												
First dose/positive test (before vaccine)	<5	-	-	0	-	-	0	-	-	8	2.27 (0.99, 5.18)	1 (Ref)
Second dose/positive test (after vaccine)	0	-	-	0	-	-	0	-	-	0	-	-
Third dose	0	-	-	0	-	-	0	-	-			
Non-white ethnicity												
First dose/positive test (before vaccine)	0	-	-	0	-	-	0	-	-	<5	-	-
Second dose/positive test (after vaccine)	0	-	-	0	-	-	0	-	-	0	-	-
Third dose	0	-	-	0	-	-	0	-	-			
**Demyelinating disease**												
White ethnicity												
First dose/positive test (before vaccine)	<5	-	-	0	-	-	0	-	-	6	2.71 (1.02, 7.19)	1 (Ref)
Second dose/positive test (after vaccine)	<5	-	-	0	-	-	0	-	-	0	-	-
Third dose	0	-	-	0	-	-	0	-	-			
Non-white ethnicity												
First dose/positive test (before vaccine)	<5	-	-	0	-	-	0	-	-	0	-	-
Second dose/positive test (after vaccine)	0	-	-	0	-	-	0	-	-	0	-	-
Third dose	0	-	-	0	-	-	0	-	-			
**Appendicitis**												
White ethnicity												
First dose/positive test (before vaccine)	48	1.01 (0.74, 1.38)	1 (Ref)	0	-	-	0	-	-	166	1.08 (0.92, 1.28)	1 (Ref)
Second dose/positive test (after vaccine)	8	0.93 (0.45, 1.93)	1 (Ref)	0	-	-	0	-	-	0	-	-
Third dose	0	-	-	0	-	-	0	-	-			
Non-white ethnicity												
First dose/positive test (before vaccine)	13	1.20 (0.64, 2.24)	1.38 (0.73, 2.64)	0	-	-	0	-	-	29	1.49 (1.00, 2.23)	1.14 (0.75, 1.73)

Second dose/positive test (after vaccine)	0	-	-	0	-	-	0	-	-	0	-	-
Third dose	0	-	-	0	-	-	0	-	-			
**Guillain-Barre syndrome**												
White ethnicity												
First dose/positive test (before vaccine)	0	-	-	0	-	-	0	-	-	0	-	-
Second dose/positive test (after vaccine)	0	-	-	0	-	-	0	-	-	0	-	-
Third dose	0	-	-	0	-	-	0	-	-			
Non-white ethnicity												
First dose/positive test (before vaccine)	0	-	-	0	-	-	0	-	-	0	-	-
Second dose/positive test (after vaccine)	0	-	-	0	-	-	0	-	-	0	-	-
Third dose	0	-	-	0	-	-	0	-	-			
**Angioedema**												
White ethnicity												
First dose/positive test (before vaccine)	<5	-	-	0	-	-	0	-	-	10	1.13 (0.56, 2.25)	1 (Ref)
Second dose/positive test (after vaccine)	0	-	-	0	-	-	0	-	-	0	-	-
Third dose	0	-	-	0	-	-	0	-	-			
Non-white ethnicity												
First dose/positive test (before vaccine)	<5	-	-	0	-	-	0	-	-	0	-	-
Second dose/positive test (after vaccine)	0	-	-	0	-	-	0	-	-	0	-	-
Third dose	0	-	-	0	-	-	0	-	-			
**Anaphylaxis**												
White ethnicity												
First dose/positive test (before vaccine)	0	-	-	0	-	-	0	-	-	11	1.04 (0.54, 2.01)	1 (Ref)
Second dose/positive test (after vaccine)	0	-	-	0	-	-	0	-	-	0	-	-
Third dose	0	-	-	0	-	-	0	-	-			
Non-white ethnicity												
First dose/positive test (before vaccine)	<5	-	-	0	-	-	0	-	-	12	2.02 (0.99, 4.14)	2.22 (0.93, 5.32)
Second dose/positive test (after vaccine)	0	-	-	0	-	-	0	-	-	0	-	-
Third dose	0	-	-	0	-	-	0	-	-			

**12-17 years**												
**Multisystem inflammatory syndrome**												
White ethnicity												
First dose/positive test (before vaccine)	15	0.91 (0.45, 1.85)	1 (Ref)	0	-	-	0	-	-	63	10.29 (6.83, 15.51)	1 (Ref)
Second dose/positive test (after vaccine)	<5	-	-	0	-	-	0	-	-	<5	-	-
Third dose	<5	-	-	0	-	-	0	-	-			
Non-white ethnicity												
First dose/positive test (before vaccine)	6	0.54 (0.17, 1.68)	0.47 (0.14, 1.59)	0	-	-	0	-	-	31	19.50 (10.04, 37.87)	1.55 (0.74, 3.25)
Second dose/positive test (after vaccine)	<5	-	-	0	-	-	0	-	-	<5	-	-
Third dose	0	-	-	0	-	-	0	-	-			
**Myocarditis**												
White ethnicity												
First dose/positive test (before vaccine)	14	1.68 (0.87, 3.25)	1 (Ref)	<5	-	-	0	-	-	5	3.56 (1.23, 10.27)	1 (Ref)
Second dose/positive test (after vaccine)	13	2.61 (1.34, 5.08)	1 (Ref)	0	-	-	0	-	-	<5	-	-
Third dose	<5	-	-	0	-	-	<5	-	-			
Non-white ethnicity												
First dose/positive test (before vaccine)	<5	-	-	0	-	-	0	-	-	<5	-	-
Second dose/positive test (after vaccine)	<5	-	-	0	-	-	0	-	-	0	-	-
Third dose	0	-	-	0	-	-	0	-	-			
**Immune or idiopathic thrombocytopenia**												
White ethnicity												
First dose/positive test (before vaccine)	34	0.89 (0.61, 1.29)	1 (Ref)	<5	-	-	0	-	-	26	1.86 (1.22, 2.82)	1 (Ref)
Second dose/positive test (after vaccine)	27	1.02 (0.67, 1.54)	1 (Ref)	<5	-	-	0	-	-	5	1.64 (0.67, 4.06)	1 (Ref)
Third dose	6	0.56 (0.25, 1.29)	1 (Ref)	0	-	-	0	-	-			
Non-white ethnicity												
First dose/positive test (before vaccine)	<5	-	-	0	-	-	0	-	-	7	1.71 (0.76, 3.85)	0.87 (0.36, 2.12)
Second dose/positive test (after vaccine)	<5	-	-	0	-	-	0	-	-	0	-	-
Third dose	<5	-	-	0	-	-	0	-	-			
**Epilepsy**												

White ethnicity												
First dose/positive test (before vaccine)	167	1.03 (0.87, 1.22)	1 (Ref)	12	1.94 (1.03, 3.65)	1 (Ref)	0	-	-	56	1.25 (0.95, 1.65)	1 (Ref)
Second dose/positive test (after vaccine)	156	1.19 (1.00, 1.41)	1 (Ref)	8	1.33 (0.62, 2.81)	1 (Ref)	<5	-	-	17	1.43 (0.88, 2.33)	1 (Ref)
Third dose	40	0.82 (0.59, 1.14)	1 (Ref)	0	-	-	6	1.13 (0.45, 2.83)	1 (Ref)			
Non-white ethnicity												
First dose/positive test (before vaccine)	33	1.06 (0.72, 1.54)	1.05 (0.71, 1.56)	<5	-	-	0	-	-	13	1.35 (0.76, 2.40)	1.09 (0.58, 2.05)
Second dose/positive test (after vaccine)	22	1.04 (0.66, 1.63)	0.87 (0.55, 1.40)	<5	-	-	0	-	-	<5	-	-
Third dose	<5	-	-	0	-	-	0	-	-			
**Acute pancreatitis**												
White ethnicity												
First dose/positive test (before vaccine)	20	1.25 (0.76, 2.07)	1 (Ref)	<5	-	-	0	-	-	<5	-	-
Second dose/positive test (after vaccine)	10	0.91 (0.46, 1.78)	1 (Ref)	0	-	-	0	-	-	<5	-	-
Third dose	<5	-	-	0	-	-	0	-	-			
Non-white ethnicity												
First dose/positive test (before vaccine)	5	1.07 (0.37, 3.13)	0.63 (0.20, 1.95)	0	-	-	0	-	-	<5	-	-
Second dose/positive test (after vaccine)	0	-	-	0	-	-	0	-	-	0	-	-
Third dose	0	-	-	0	-	-	0	-	-			
**Myositis**												
White ethnicity												
First dose/positive test (before vaccine)	<5	-	-	0	-	-	0	-	-	<5	-	-
Second dose/positive test (after vaccine)	<5	-	-	<5	-	-	0	-	-	0	-	-
Third dose	<5	-	-	0	-	-	0	-	-			
Non-white ethnicity												
First dose/positive test (before vaccine)	<5	-	-	0	-	-	0	-	-	0	-	-
Second dose/positive test (after vaccine)	0	-	-	0	-	-	0	-	-	0	-	-
Third dose	0	-	-	0	-	-	0	-	-			
**Acute disseminated encephalomyelitis**												
White ethnicity												
First dose/positive test (before vaccine)	<5	-	-	0	-	-	0	-	-	<5	-	-

Second dose/positive test (after vaccine)	5	1.22 (0.46, 3.24)	1 (Ref)	0	-	-	0	-	-	<5	-	-
Third dose	0	-	-	0	-	-	0	-	-			
Non-white ethnicity												
First dose/positive test (before vaccine)	<5	-	-	0	-	-	0	-	-	0	-	-
Second dose/positive test (after vaccine)	<5	-	-	0	-	-	0	-	-	<5	-	-
Third dose	0	-	-	0	-	-	0	-	-			
**Demyelinating disease**												
White ethnicity												
First dose/positive test (before vaccine)	7	1.27 (0.56, 2.90)	1 (Ref)	0	-	-	0	-	-	<5	-	-
Second dose/positive test (after vaccine)	8	1.69 (0.77, 3.72)	1 (Ref)	0	-	-	0	-	-	<5	-	-
Third dose	<5	-	-	0	-	-	0	-	-			
Non-white ethnicity												
First dose/positive test (before vaccine)	0	-	-	0	-	-	0	-	-	0	-	-
Second dose/positive test (after vaccine)	<5	-	-	0	-	-	0	-	-	0	-	-
Third dose	0	-	-	0	-	-	0	-	-			
**Appendicitis**												
White ethnicity												
First dose/positive test (before vaccine)	373	0.89 (0.79, 1.00)	1 (Ref)	<5	-	-	0	-	-	171	1.07 (0.91, 1.25)	1 (Ref)
Second dose/positive test (after vaccine)	271	0.88 (0.77, 1.01)	1 (Ref)	6	6.02 (2.20, 16.49)	1 (Ref)	0	-	-	19	0.69 (0.44, 1.09)	1 (Ref)
Third dose	60	1.14 (0.87, 1.49)	1 (Ref)	0	-	-	8	1.27 (0.61, 2.62)	1 (Ref)			
Non-white ethnicity												
First dose/positive test (before vaccine)	53	0.95 (0.70, 1.29)	1.02 (0.75, 1.37)	0	-	-	0	-	-	30	1.30 (0.89, 1.91)	1.15 (0.77, 1.73)
Second dose/positive test (after vaccine)	36	0.95 (0.66, 1.36)	1.07 (0.74, 1.54)	0	-	-	0	-	-	<5	-	-
Third dose	<5	-	-	0	-	-	<5	-	-			
**Guillain-Barre syndrome**												
White ethnicity												
First dose/positive test (before vaccine)	<5	-	-	0	-	-	0	-	-	<5	-	-
Second dose/positive test (after vaccine)	<5	-	-	0	-	-	0	-	-	<5	-	-
Third dose	0	-	-	0	-	-	0	-	-			

Non-white ethnicity												
First dose/positive test (before vaccine)	0	-	-	0	-	-	0	-	-	<5	-	-
Second dose/positive test (after vaccine)	0	-	-	0	-	-	0	-	-	0	-	-
Third dose	0	-	-	0	-	-	0	-	-			
**Angioedema**												
White ethnicity												
First dose/positive test (before vaccine)	18	1.25 (0.74, 2.14)	1 (Ref)	0	-	-	0	-	-	6	1.00 (0.43, 2.33)	1 (Ref)
Second dose/positive test (after vaccine)	7	0.67 (0.30, 1.47)	1 (Ref)	<5	-	-	0	-	-	<5	-	-
Third dose	<5	-	-	0	-	-	<5	-	-			
Non-white ethnicity												
First dose/positive test (before vaccine)	7	2.12 (0.88, 5.15)	1.44 (0.56, 3.67)	<5	-	-	0	-	-	<5	-	-
Second dose/positive test (after vaccine)	5	1.79 (0.65, 4.92)	2.78 (0.82, 9.38)	0	-	-	0	-	-	0	-	-
Third dose	<5	-	-	0	-	-	0	-	-			
**Anaphylaxis**												
White ethnicity												
First dose/positive test (before vaccine)	27	1.13 (0.74, 1.74)	1 (Ref)	0	-	-	0	-	-	14	1.55 (0.88, 2.72)	1 (Ref)
Second dose/positive test (after vaccine)	11	0.60 (0.32, 1.13)	1 (Ref)	0	-	-	0	-	-	<5	-	-
Third dose	5	1.21 (0.47, 3.10)	1 (Ref)	0	-	-	<5	-	-			
Non-white ethnicity												
First dose/positive test (before vaccine)	15	1.83 (0.99, 3.38)	1.49 (0.76, 2.93)	0	-	-	0	-	-	<5	-	-
Second dose/positive test (after vaccine)	9	1.69 (0.80, 3.54)	2.55 (1.00, 6.46)	0	-	-	0	-	-	<5	-	-
Third dose	<5	-	-	0	-	-	0	-	-			
**18-24 years**												
**Multisystem inflammatory syndrome**												
White ethnicity												
First dose/positive test (before vaccine)	<5	-	-	<5	-	-	0	-	-	<5	-	-
Second dose/positive test (after vaccine)	<5	-	-	<5	-	-	0	-	-	<5	-	-
Third dose	0	-	-	0	-	-	0	-	-			
Non-white ethnicity												

First dose/positive test (before vaccine)	<5	-	-	0	-	-	0	-	-	<5	-	-
Second dose/positive test (after vaccine)	0	-	-	0	-	-	0	-	-	0	-	-
Third dose	0	-	-	0	-	-	0	-	-			
**Myocarditis**												
White ethnicity												
First dose/positive test (before vaccine)	16	0.80 (0.46, 1.41)	1 (Ref)	5	2.17 (0.80, 5.92)	1 (Ref)	7	2.01 (0.79, 5.13)	1 (Ref)	8	1.85 (0.87, 3.92)	1 (Ref)
Second dose/positive test (after vaccine)	39	2.31 (1.56, 3.43)	1 (Ref)	9	2.03 (0.94, 4.40)	1 (Ref)	23	10.54 (5.49, 20.22)	1 (Ref)	<5	-	-
Third dose	24	3.02 (1.83, 5.00)	1 (Ref)	0	-	-	10	4.05 (1.87, 8.81)	1 (Ref)			
Non-white ethnicity												
First dose/positive test (before vaccine)	<5	-	-	0	-	-	<5	-	-	7	7.63 (2.77, 20.96)	3.46 (1.05, 11.44)
Second dose/positive test (after vaccine)	5	3.99 (1.36, 11.66)	1.37 (0.47, 4.01)	0	-	-	<5	-	-	0	-	-
Third dose	<5	-	-	0	-	-	<5	-	-			
**Immune or idiopathic thrombocytopenia**												
White ethnicity												
First dose/positive test (before vaccine)	31	0.64 (0.44, 0.94)	1 (Ref)	27	1.24 (0.80, 1.93)	1 (Ref)	<5	-	-	37	3.79 (2.62, 5.49)	1 (Ref)
Second dose/positive test (after vaccine)	42	1.08 (0.78, 1.50)	1 (Ref)	25	1.12 (0.72, 1.75)	1 (Ref)	5	1.31 (0.51, 3.33)	1 (Ref)	11	2.07 (1.12, 3.83)	1 (Ref)
Third dose	25	0.78 (0.50, 1.22)	1 (Ref)	0	-	-	8	0.85 (0.41, 1.78)	1 (Ref)			
Non-white ethnicity												
First dose/positive test (before vaccine)	13	1.04 (0.57, 1.88)	1.53 (0.77, 3.02)	6	1.84 (0.73, 4.62)	1.26 (0.49, 3.25)	<5	-	-	14	4.62 (2.49, 8.57)	1.13 (0.56, 2.29)
Second dose/positive test (after vaccine)	7	0.63 (0.29, 1.37)	0.59 (0.26, 1.36)	<5	-	-	0	-	-	<5	-	-
Third dose	6	0.87 (0.34, 2.21)	1.14 (0.41, 3.12)	0	-	-	<5	-	-			
**Epilepsy**												
White ethnicity												
First dose/positive test (before vaccine)	120	0.81 (0.67, 0.98)	1 (Ref)	166	1.15 (0.59, 2.25)	1 (Ref)	5	0.55 (0.22, 1.36)	1 (Ref)	54	1.39 (1.05, 1.85)	1 (Ref)
Second dose/positive test (after vaccine)	119	0.87 (0.72, 1.05)	1 (Ref)	165	1.38 (0.77, 2.47)	1 (Ref)	<5	-	-	35	1.49 (1.06, 2.09)	1 (Ref)
Third dose	142	1.05 (0.87, 1.27)	1 (Ref)	<5	-	-	35	0.83 (0.58, 1.20)	1 (Ref)			
Non-white ethnicity												
First dose/positive test (before vaccine)	30	1.32 (0.90, 1.95)	1.60 (1.05, 2.45)	18	1.20 (0.38, 3.83)	0.81 (0.49, 1.34)	<5	-	-	16	2.42 (1.41, 4.15)	1.75 (0.96, 3.20)

Second dose/positive test (after vaccine)	16	0.87 (0.52, 1.46)	0.95 (0.55, 1.64)	22	0.72 (0.16, 3.32)	1.04 (0.65, 1.66)	<5	-	-	<5	-	-
Third dose	11	0.66 (0.34, 1.26)	0.64 (0.33, 1.25)	0	-	-	<5	-	-			
**Acute pancreatitis**												
White ethnicity												
First dose/positive test (before vaccine)	51	0.99 (0.72, 1.35)	1 (Ref)	9	0.49 (0.24, 0.97)	1 (Ref)	8	1.91 (0.89, 4.14)	1 (Ref)	22	1.83 (1.16, 2.87)	1 (Ref)
Second dose/positive test (after vaccine)	47	1.05 (0.76, 1.45)	1 (Ref)	23	1.19 (0.75, 1.88)	1 (Ref)	<5	-	-	6	0.92 (0.41, 2.08)	1 (Ref)
Third dose	33	1.26 (0.85, 1.85)	1 (Ref)	<5	-	-	10	0.94 (0.48, 1.83)	1 (Ref)			
Non-white ethnicity												
First dose/positive test (before vaccine)	9	1.13 (0.55, 2.36)	0.90 (0.43, 1.89)	0	-	-	0	-	-	5	1.95 (0.75, 5.12)	0.86 (0.31, 2.43)
Second dose/positive test (after vaccine)	7	1.14 (0.51, 2.57)	0.85 (0.37, 1.95)	<5	-	-	<5	-	-	0	-	-
Third dose	<5	-	-	0	-	-	<5	-	-			
**Myositis**												
White ethnicity												
First dose/positive test (before vaccine)	<5	-	-	0	-	-	0	-	-	<5	-	-
Second dose/positive test (after vaccine)	<5	-	-	0	-	-	<5	-	-	<5	-	-
Third dose	<5	-	-	0	-	-	0	-	-			
Non-white ethnicity												
First dose/positive test (before vaccine)	<5	-	-	<5	-	-	0	-	-	0	-	-
Second dose/positive test (after vaccine)	0	-	-	0	-	-	0	-	-	0	-	-
Third dose	0	-	-	0	-	-	0	-	-			
**Acute disseminated encephalomyelitis**												
White ethnicity												
First dose/positive test (before vaccine)	9	2.05 (0.95, 4.42)	1 (Ref)	<5	-	-	0	-	-	<5	-	-
Second dose/positive test (after vaccine)	6	1.51 (0.61, 3.75)	1 (Ref)	<5	-	-	<5	-	-	<5	-	-
Third dose	<5	-	-	0	-	-	<5	-	-			
Non-white ethnicity												
First dose/positive test (before vaccine)	0	-	-	0	-	-	<5	-	-	0	-	-
Second dose/positive test (after vaccine)	0	-	-	<5	-	-	0	-	-	0	-	-
Third dose	0	-	-	0	-	-	0	-	-			

**Demyelinating disease**												
White ethnicity												
First dose/positive test (before vaccine)	8	0.59 (0.28, 1.25)	1 (Ref)	6	1.17 (0.46, 2.95)	1 (Ref)	0	-	-	<5	-	-
Second dose/positive test (after vaccine)	7	0.63 (0.28, 1.37)	1 (Ref)	<5	-	-	<5	-	-	<5	-	-
Third dose	7	0.86 (0.38, 1.97)	1 (Ref)	0	-	-	<5	-	-			
Non-white ethnicity												
First dose/positive test (before vaccine)	<5	-	-	0	-	-	0	-	-	<5	-	-
Second dose/positive test (after vaccine)	<5	-	-	0	-	-	0	-	-	0	-	-
Third dose	<5	-	-	0	-	-	0	-	-			
**Appendicitis**												
White ethnicity												
First dose/positive test (before vaccine)	355	0.98 (0.86, 1.11)	1 (Ref)	89	1.07 (0.85, 1.35)	1 (Ref)	47	1.19 (0.87, 1.64)	1 (Ref)	89	1.27 (1.02, 1.58)	1 (Ref)
Second dose/positive test (after vaccine)	300	0.97 (0.85, 1.10)	1 (Ref)	80	1.07 (0.84, 1.36)	1 (Ref)	39	1.22 (0.87, 1.70)	1 (Ref)	42	1.15 (0.84, 1.56)	1 (Ref)
Third dose	157	0.99 (0.83, 1.18)	1 (Ref)	0	-	-	79	1.06 (0.84, 1.35)	1 (Ref)			
Non-white ethnicity												
First dose/positive test (before vaccine)	59	1.08 (0.80, 1.44)	1.06 (0.79, 1.42)	8	1.01 (0.48, 2.11)	0.74 (0.35, 1.57)	5	0.79 (0.32, 1.97)	0.67 (0.26, 1.76)	17	1.22 (0.74, 2.01)	0.88 (0.51, 1.52)
Second dose/positive test (after vaccine)	35	0.70 (0.49, 1.00)	0.79 (0.55, 1.13)	5	0.60 (0.24, 1.50)	0.52 (0.20, 1.31)	5	0.93 (0.37, 2.34)	0.85 (0.32, 2.26)	9	2.40 (1.22, 4.76)	2.22 (1.06, 4.66)
Third dose	18	0.96 (0.58, 1.58)	1.02 (0.62, 1.70)	0	-	-	6	1.05 (0.45, 2.45)	1.08 (0.46, 2.57)			
**Guillain-Barre syndrome**												
White ethnicity												
First dose/positive test (before vaccine)	<5	-	-	0	-	-	<5	-	-	0	-	-
Second dose/positive test (after vaccine)	<5	-	-	0	-	-	0	-	-	0	-	-
Third dose	<5	-	-	0	-	-	<5	-	-			
Non-white ethnicity												
First dose/positive test (before vaccine)	0	-	-	<5	-	-	0	-	-	<5	-	-
Second dose/positive test (after vaccine)	<5	-	-	0	-	-	0	-	-	0	-	-
Third dose	<5	-	-	0	-	-	<5	-	-			
**Angioedema**												
White ethnicity												

First dose/positive test (before vaccine)	17	0.67 (0.39, 1.15)	1 (Ref)	16	1.32 (0.74, 2.37)	1 (Ref)	<5	-	-	<5	-	-
Second dose/positive test (after vaccine)	20	1.11 (0.67, 1.83)	1 (Ref)	10	0.83 (0.42, 1.67)	1 (Ref)	<5	-	-	5	2.11 (0.85, 5.26)	1 (Ref)
Third dose	5	0.52 (0.21, 1.29)	1 (Ref)	0	-	-	<5	-	-			
Non-white ethnicity												
First dose/positive test (before vaccine)	5	1.24 (0.45, 3.41)	1.47 (0.51, 4.23)	0	-	-	0	-	-	<5	-	-
Second dose/positive test (after vaccine)	<5	-	-	0	-	-	0	-	-	0	-	-
Third dose	0	-	-	0	-	-	0	-	-			
**Anaphylaxis**												
White ethnicity												
First dose/positive test (before vaccine)	28	1.11 (0.73, 1.71)	1 (Ref)	11		1 (Ref)	<5	-	-	6	0.97 (0.42, 2.25)	1 (Ref)
Second dose/positive test (after vaccine)	21	0.99 (0.62, 1.59)	1 (Ref)	16		1 (Ref)	<5	-	-	<5	-	-
Third dose	13	1.05 (0.56, 1.96)	1 (Ref)	0	-	-	5	1.06 (0.42, 2.72)	1 (Ref)			
Non-white ethnicity												
First dose/positive test (before vaccine)	7	0.93 (0.41, 2.13)	0.98 (0.41, 2.36)	<5	-	-	0	-	-	<5	-	-
Second dose/positive test (after vaccine)	<5	-	-	<5	-	-	<5	-	-	<5	-	-
Third dose	<5	-	-	0	-	-	0	-	-			

Supplementary Table 7. Incidence rate ratios and estimated number of excess events per million in pre-specified weekly periods in the 1-42 days following vaccination or a SARS-CoV-2 positive test (before or after vaccination) compared to baseline period in children aged 5-11 years. Cells with a hyphen are suppressed; N – number of events; IRR – incidence rate ratio; CI – confidence interval.

	BNT162b2 vaccine		mRNA-1273 vaccine		ChAdOX1 vaccine		Positive SARS-CoV-2 test	
	N	IRR (95% CI)	N	IRR (95% CI)	N	IRR (95% CI)	N	IRR (95% CI)
**Multisystem inflammatory syndrome**								
baseline	138	1.00 (1.00, 1.00)	0	-	0	-	163	1.00 (1.00, 1.00)
-28-1 days: first dose/positive test (before vaccine)	0	-	0	-	0	-	21	1.62 (1.02, 2.59)
0 day: first dose/positive test (before vaccine)	0	-	0	-	0	-	21	43.95 (27.59, 70.01)
1-7 days: first dose/positive test (before vaccine)	0	-	0	-	0	-	15	4.46 (2.60, 7.63)
8-14 days: first dose/positive test (before vaccine)	0	-	0	-	0	-	<5	-
15-21 days: first dose/positive test (before vaccine)	0	-	0	-	0	-	10	2.99 (1.57, 5.70)
22-28 days: first dose/positive test (before vaccine)	0	-	0	-	0	-	35	10.59 (7.26, 15.47)
29-42 days: first dose/positive test (before vaccine)	<5	-	0	-	0	-	162	25.27 (19.89, 32.12)
-28-1 days: second dose/positive test (after vaccine)	<5	-	0	-	0	-	0	-
0 day: second dose/positive test (after vaccine)	0	-	0	-	0	-	0	-
1-7 days: second dose/positive test (after vaccine)	0	-	0	-	0	-	0	-
8-14 days: second dose/positive test (after vaccine)	0	-	0	-	0	-	0	-
15-21 days: second dose/positive test (after vaccine)	0	-	0	-	0	-	0	-
22-28 days: second dose/positive test (after vaccine)	0	-	0	-	0	-	0	-
29-42 days: second dose/positive test (after vaccine)	0	-	0	-	0	-	0	-
**Myocarditis**								
baseline	<5	-	0	-	0	-	5	1.00 (1.00, 1.00)
-28-1 days: first dose/positive test (before vaccine)	0	-	0	-	0	-	0	-
0 day: first dose/positive test (before vaccine)	0	-	0	-	0	-	<5	-
1-7 days: first dose/positive test (before vaccine)	0	-	0	-	0	-	<5	-
8-14 days: first dose/positive test (before vaccine)	0	-	0	-	0	-	0	-
15-21 days: first dose/positive test (before vaccine)	0	-	0	-	0	-	<5	-
22-28 days: first dose/positive test (before vaccine)	0	-	0	-	0	-	<5	-
29-42 days: first dose/positive test (before vaccine)	0	-	0	-	0	-	<5	-
-28-1 days: second dose/positive test (after vaccine)	0	-	0	-	0	-	0	-
0 day: second dose/positive test (after vaccine)	0	-	0	-	0	-	0	-
1-7 days: second dose/positive test (after vaccine)	0	-	0	-	0	-	0	-
8-14 days: second dose/positive test (after vaccine)	0	-	0	-	0	-	0	-
15-21 days: second dose/positive test (after vaccine)	0	-	0	-	0	-	0	-
22-28 days: second dose/positive test (after vaccine)	0	-	0	-	0	-	0	-
29-42 days: second dose/positive test (after vaccine)	0	-	0	-	0	-	0	-
**Immune or idiopathic thrombocytopenia**								
baseline	151	-	0	-	0	-	360	-
-28-1 days: first dose/positive test (before vaccine)	5	-	0	-	0	-	20	-
0 day: first dose/positive test (before vaccine)	0	-	0	-	0	-	15	-

1-7 days: first dose/positive test (before vaccine)	<5	-	0	-	0	-	10	-
8-14 days: first dose/positive test (before vaccine)	0	-	0	-	0	-	7	-
15-21 days: first dose/positive test (before vaccine)	<5	-	0	-	0	-	13	-
22-28 days: first dose/positive test (before vaccine)	<5	-	0	-	0	-	8	-
29-42 days: first dose/positive test (before vaccine)	<5	-	0	-	0	-	23	-
-28-1 days: second dose/positive test (after vaccine)	<5	-	0	-	0	-	<5	-
0 day: second dose/positive test (after vaccine)	0	-	0	-	0	-	0	-
1-7 days: second dose/positive test (after vaccine)	0	-	0	-	0	-	0	-
8-14 days: second dose/positive test (after vaccine)	0	-	0	-	0	-	0	-
15-21 days: second dose/positive test (after vaccine)	0	-	0	-	0	-	0	-
22-28 days: second dose/positive test (after vaccine)	0	-	0	-	0	-	0	-
29-42 days: second dose/positive test (after vaccine)	<5	-	0	-	0	-	0	-
**Epilepsy**								
baseline	780	1.00 (1.00, 1.00)	0	-	0	-	1275	1.00 (1.00, 1.00)
-28-1 days: first dose/positive test (before vaccine)	41	1.02 (0.73, 1.42)	0	-	0	-	66	1.12 (0.87, 1.44)
0 day: first dose/positive test (before vaccine)	0	-	0	-	0	-	49	23.22 (17.33, 31.10)
1-7 days: first dose/positive test (before vaccine)	6	0.57 (0.25, 1.29)	0	-	0	-	17	1.16 (0.72, 1.88)
8-14 days: first dose/positive test (before vaccine)	17	1.63 (0.99, 2.68)	0	-	0	-	18	1.23 (0.77, 1.97)
15-21 days: first dose/positive test (before vaccine)	12	1.17 (0.65, 2.10)	0	-	0	-	15	1.02 (0.61, 1.70)
22-28 days: first dose/positive test (before vaccine)	6	0.61 (0.27, 1.37)	0	-	0	-	10	0.67 (0.36, 1.26)
29-42 days: first dose/positive test (before vaccine)	20	1.02 (0.64, 1.62)	0	-	0	-	40	1.34 (0.97, 1.85)
-28-1 days: second dose/positive test (after vaccine)	6	0.32 (0.14, 0.72)	0	-	0	-	<5	-
0 day: second dose/positive test (after vaccine)	0	-	0	-	0	-	<5	-
1-7 days: second dose/positive test (after vaccine)	9	1.66 (0.85, 3.25)	0	-	0	-	<5	-
8-14 days: second dose/positive test (after vaccine)	6	1.16 (0.51, 2.61)	0	-	0	-	0	-
15-21 days: second dose/positive test (after vaccine)	<5	-	0	-	0	-	0	-
22-28 days: second dose/positive test (after vaccine)	<5	-	0	-	0	-	0	-
29-42 days: second dose/positive test (after vaccine)	7	0.81 (0.38, 1.73)	0	-	0	-	<5	-
**Acute pancreatitis**								
baseline	17	-	0	-	0	-	38	-
-28-1 days: first dose/positive test (before vaccine)	0	-	0	-	0	-	<5	-
0 day: first dose/positive test (before vaccine)	0	-	0	-	0	-	<5	-
1-7 days: first dose/positive test (before vaccine)	0	-	0	-	0	-	5	-
8-14 days: first dose/positive test (before vaccine)	0	-	0	-	0	-	0	-
15-21 days: first dose/positive test (before vaccine)	0	-	0	-	0	-	<5	-
22-28 days: first dose/positive test (before vaccine)	0	-	0	-	0	-	<5	-
29-42 days: first dose/positive test (before vaccine)	<5	-	0	-	0	-	<5	-
-28-1 days: second dose/positive test (after vaccine)	0	-	0	-	0	-	0	-
0 day: second dose/positive test (after vaccine)	0	-	0	-	0	-	0	-
1-7 days: second dose/positive test (after vaccine)	0	-	0	-	0	-	0	-
8-14 days: second dose/positive test (after vaccine)	0	-	0	-	0	-	0	-
15-21 days: second dose/positive test (after vaccine)	0	-	0	-	0	-	0	-
22-28 days: second dose/positive test (after vaccine)	0	-	0	-	0	-	0	-

29-42 days: second dose/positive test (after vaccine)	0	-	0	-	0	-	<5	-
**Myositis**								
baseline	21	1.00 (1.00, 1.00)	0	-	0	-	51	1.00 (1.00, 1.00)
-28-1 days: first dose/positive test (before vaccine)	<5	-	0	-	0	-	<5	-
0 day: first dose/positive test (before vaccine)	0	-	0	-	0	-	6	103.73 (39.97, 269.22)
1-7 days: first dose/positive test (before vaccine)	0	-	0	-	0	-	8	21.06 (8.88, 49.98)
8-14 days: first dose/positive test (before vaccine)	0	-	0	-	0	-	<5	-
15-21 days: first dose/positive test (before vaccine)	<5	-	0	-	0	-	<5	-
22-28 days: first dose/positive test (before vaccine)	0	-	0	-	0	-	0	-
29-42 days: first dose/positive test (before vaccine)	0	-	0	-	0	-	0	-
-28-1 days: second dose/positive test (after vaccine)	0	-	0	-	0	-	0	-
0 day: second dose/positive test (after vaccine)	0	-	0	-	0	-	0	-
1-7 days: second dose/positive test (after vaccine)	0	-	0	-	0	-	0	-
8-14 days: second dose/positive test (after vaccine)	0	-	0	-	0	-	0	-
15-21 days: second dose/positive test (after vaccine)	0	-	0	-	0	-	0	-
22-28 days: second dose/positive test (after vaccine)	0	-	0	-	0	-	0	-
29-42 days: second dose/positive test (after vaccine)	0	-	0	-	0	-	0	-
**Acute disseminated encephalomyelitis**								
baseline	27	1.00 (1.00, 1.00)	0	-	0	-	61	1.00 (1.00, 1.00)
-28-1 days: first dose/positive test (before vaccine)	0	-	0	-	0	-	<5	-
0 day: first dose/positive test (before vaccine)	0	-	0	-	0	-	6	54.23 (22.21, 132.40)
1-7 days: first dose/positive test (before vaccine)	0	-	0	-	0	-	<5	-
8-14 days: first dose/positive test (before vaccine)	<5	-	0	-	0	-	<5	-
15-21 days: first dose/positive test (before vaccine)	0	-	0	-	0	-	<5	-
22-28 days: first dose/positive test (before vaccine)	0	-	0	-	0	-	<5	-
29-42 days: first dose/positive test (before vaccine)	0	-	0	-	0	-	<5	-
-28-1 days: second dose/positive test (after vaccine)	0	-	0	-	0	-	0	-
0 day: second dose/positive test (after vaccine)	0	-	0	-	0	-	0	-
1-7 days: second dose/positive test (after vaccine)	0	-	0	-	0	-	0	-
8-14 days: second dose/positive test (after vaccine)	0	-	0	-	0	-	0	-
15-21 days: second dose/positive test (after vaccine)	0	-	0	-	0	-	0	-
22-28 days: second dose/positive test (after vaccine)	0	-	0	-	0	-	0	-
29-42 days: second dose/positive test (after vaccine)	0	-	0	-	0	-	0	-
**Demyelinating disease**								
baseline	16	1.00 (1.00, 1.00)	0	-	0	-	46	1.00 (1.00, 1.00)
-28-1 days: first dose/positive test (before vaccine)	<5	-	0	-	0	-	<5	-
0 day: first dose/positive test (before vaccine)	0	-	0	-	0	-	0	-
1-7 days: first dose/positive test (before vaccine)	0	-	0	-	0	-	0	-
8-14 days: first dose/positive test (before vaccine)	<5	-	0	-	0	-	0	-
15-21 days: first dose/positive test (before vaccine)	0	-	0	-	0	-	<5	-
22-28 days: first dose/positive test (before vaccine)	0	-	0	-	0	-	<5	-
29-42 days: first dose/positive test (before vaccine)	<5	-	0	-	0	-	<5	-
-28-1 days: second dose/positive test (after vaccine)	<5	-	0	-	0	-	0	-

0 day: second dose/positive test (after vaccine)	0	-	0	-	0	-	0	-
1-7 days: second dose/positive test (after vaccine)	0	-	0	-	0	-	0	-
8-14 days: second dose/positive test (after vaccine)	<5	-	0	-	0	-	0	-
15-21 days: second dose/positive test (after vaccine)	0	-	0	-	0	-	0	-
22-28 days: second dose/positive test (after vaccine)	0	-	0	-	0	-	0	-
29-42 days: second dose/positive test (after vaccine)	0	-	0	-	0	-	0	-
**Appendicitis**								
baseline	823	1.00 (1.00, 1.00)	0	-	0	-	2346	1.00 (1.00, 1.00)
-28-1 days: first dose/positive test (before vaccine)	30	0.70 (0.48, 1.02)	0	-	0	-	91	0.74 (0.60, 0.92)
0 day: first dose/positive test (before vaccine)	0	-	0	-	0	-	119	26.72 (22.07, 32.34)
1-7 days: first dose/positive test (before vaccine)	11	1.03 (0.56, 1.88)	0	-	0	-	40	1.29 (0.94, 1.78)
8-14 days: first dose/positive test (before vaccine)	15	1.37 (0.81, 2.31)	0	-	0	-	36	1.18 (0.84, 1.64)
15-21 days: first dose/positive test (before vaccine)	10	0.93 (0.49, 1.75)	0	-	0	-	30	0.99 (0.69, 1.42)
22-28 days: first dose/positive test (before vaccine)	9	0.88 (0.45, 1.71)	0	-	0	-	30	0.98 (0.68, 1.41)
29-42 days: first dose/positive test (before vaccine)	18	0.95 (0.59, 1.54)	0	-	0	-	77	1.22 (0.96, 1.54)
-28-1 days: second dose/positive test (after vaccine)	11	0.70 (0.38, 1.29)	0	-	0	-	<5	-
0 day: second dose/positive test (after vaccine)	0	-	0	-	0	-	0	-
1-7 days: second dose/positive test (after vaccine)	<5	-	0	-	0	-	0	-
8-14 days: second dose/positive test (after vaccine)	0	-	0	-	0	-	<5	-
15-21 days: second dose/positive test (after vaccine)	<5	-	0	-	0	-	0	-
22-28 days: second dose/positive test (after vaccine)	0	-	0	-	0	-	0	-
29-42 days: second dose/positive test (after vaccine)	<5	-	0	-	0	-	0	-
**Guillain-Barre Syndrome**								
baseline	<5	-	0	-	0	-	15	-
-28-1 days: first dose/positive test (before vaccine)	0	-	0	-	0	-	0	-
0 day: first dose/positive test (before vaccine)	0	-	0	-	0	-	<5	-
1-7 days: first dose/positive test (before vaccine)	0	-	0	-	0	-	0	-
8-14 days: first dose/positive test (before vaccine)	0	-	0	-	0	-	0	-
15-21 days: first dose/positive test (before vaccine)	0	-	0	-	0	-	0	-
22-28 days: first dose/positive test (before vaccine)	0	-	0	-	0	-	0	-
29-42 days: first dose/positive test (before vaccine)	0	-	0	-	0	-	0	-
-28-1 days: second dose/positive test (after vaccine)	0	-	0	-	0	-	0	-
0 day: second dose/positive test (after vaccine)	0	-	0	-	0	-	0	-
1-7 days: second dose/positive test (after vaccine)	0	-	0	-	0	-	0	-
8-14 days: second dose/positive test (after vaccine)	0	-	0	-	0	-	0	-
15-21 days: second dose/positive test (after vaccine)	0	-	0	-	0	-	0	-
22-28 days: second dose/positive test (after vaccine)	0	-	0	-	0	-	0	-
29-42 days: second dose/positive test (after vaccine)	0	-	0	-	0	-	0	-
**Angioedema**								
baseline	54	-	0	-	0	-	173	-
-28-1 days: first dose/positive test (before vaccine)	<5	-	0	-	0	-	13	-
0 day: first dose/positive test (before vaccine)	0	-	0	-	0	-	<5	-
1-7 days: first dose/positive test (before vaccine)	0	-	0	-	0	-	<5	-

8-14 days: first dose/positive test (before vaccine)	<5	-	0	-	0	-	<5	-
15-21 days: first dose/positive test (before vaccine)	0	-	0	-	0	-	<5	-
22-28 days: first dose/positive test (before vaccine)	<5	-	0	-	0	-	0	-
29-42 days: first dose/positive test (before vaccine)	<5	-	0	-	0	-	<5	-
-28-1 days: second dose/positive test (after vaccine)	0	-	0	-	0	-	0	-
0 day: second dose/positive test (after vaccine)	0	-	0	-	0	-	0	-
1-7 days: second dose/positive test (after vaccine)	0	-	0	-	0	-	0	-
8-14 days: second dose/positive test (after vaccine)	0	-	0	-	0	-	0	-
15-21 days: second dose/positive test (after vaccine)	0	-	0	-	0	-	0	-
22-28 days: second dose/positive test (after vaccine)	0	-	0	-	0	-	0	-
29-42 days: second dose/positive test (after vaccine)	0	-	0	-	0	-	0	-
**Anaphylaxis**								
baseline	70	1.00 (1.00, 1.00)	0	-	0	-	208	1.00 (1.00, 1.00)
-28-1 days: first dose/positive test (before vaccine)	<5	-	0	-	0	-	8	0.74 (0.36, 1.55)
0 day: first dose/positive test (before vaccine)	0	-	0	-	0	-	<5	-
1-7 days: first dose/positive test (before vaccine)	<5	-	0	-	0	-	<5	-
8-14 days: first dose/positive test (before vaccine)	0	-	0	-	0	-	<5	-
15-21 days: first dose/positive test (before vaccine)	0	-	0	-	0	-	5	1.80 (0.72, 4.48)
22-28 days: first dose/positive test (before vaccine)	0	-	0	-	0	-	<5	-
29-42 days: first dose/positive test (before vaccine)	<5	-	0	-	0	-	10	1.80 (0.93, 3.51)
-28-1 days: second dose/positive test (after vaccine)	<5	-	0	-	0	-	0	-
0 day: second dose/positive test (after vaccine)	0	-	0	-	0	-	0	-
1-7 days: second dose/positive test (after vaccine)	0	-	0	-	0	-	0	-
8-14 days: second dose/positive test (after vaccine)	0	-	0	-	0	-	0	-
15-21 days: second dose/positive test (after vaccine)	0	-	0	-	0	-	0	-
22-28 days: second dose/positive test (after vaccine)	0	-	0	-	0	-	0	-
29-42 days: second dose/positive test (after vaccine)	0	-	0	-	0	-	0	-

Supplementary Table 8. Incidence rate ratios and estimated number of excess events per million in pre-specified weekly periods in the 1-42 days following vaccination or a SARS-CoV-2 positive test (before or after vaccination) compared to baseline period in adolescents aged 12-17 years. N – number of events; IRR – incidence rate ratio; CI – confidence interval

	BNT162b2 vaccine		mRNA-1273 vaccine		ChAdOX1 vaccine		Positive SARS-CoV-2 test	
	N	IRR (95% CI)	N	IRR (95% CI)	N	IRR (95% CI)	N	IRR (95% CI)
**Multisystem inflammatory syndrome**								
baseline	208	1.00 (1.00, 1.00)	0	-	<5	-	78	1.00 (1.00, 1.00)
-28-1 days: first dose/positive test (before vaccine)	5	0.36 (0.14, 0.91)	0	-	0	-	15	2.92 (1.64, 5.19)
0 day: first dose/positive test (before vaccine)	0	-	0	-	0	-	22	114.10 (69.29, 187.91)
1-7 days: first dose/positive test (before vaccine)	<5	-	0	-	0	-	9	6.75 (3.33, 13.69)
8-14 days: first dose/positive test (before vaccine)	<5	-	0	-	0	-	<5	-
15-21 days: first dose/positive test (before vaccine)	<5	-	0	-	0	-	5	3.79 (1.51, 9.51)
22-28 days: first dose/positive test (before vaccine)	<5	-	0	-	0	-	13	10.03 (5.45, 18.46)
29-42 days: first dose/positive test (before vaccine)	10	1.03 (0.49, 2.18)	0	-	0	-	67	25.49 (17.70, 36.72)
-28-1 days: second dose/positive test (after vaccine)	<5	-	0	-	0	-	<5	-
0 day: second dose/positive test (after vaccine)	0	-	0	-	0	-	0	-
1-7 days: second dose/positive test (after vaccine)	<5	-	0	-	0	-	<5	-
8-14 days: second dose/positive test (after vaccine)	0	-	0	-	0	-	0	-
15-21 days: second dose/positive test (after vaccine)	<5	-	0	-	0	-	0	-
22-28 days: second dose/positive test (after vaccine)	0	-	0	-	0	-	6	38.15 (11.80, 123.29)
29-42 days: second dose/positive test (after vaccine)	0	-	0	-	0	-	7	23.08 (7.60, 70.12)
-28-1 days: third dose	<5	-	0	-	0	-		
0 day: third dose	0	-	0	-	0	-		
1-7 days: third dose	0	-	0	-	0	-		
8-14 days: third dose	0	-	0	-	0	-		
15-21 days: third dose	0	-	0	-	0	-		
22-28 days: third dose	0	-	0	-	0	-		
29-42 days: third dose	<5	-	0	-	0	-		
**Myocarditis**								
baseline	82	1.00 (1.00, 1.00)	5	1.00 (1.00, 1.00)	<5	-	62	1.00 (1.00, 1.00)
-28-1 days: first dose/positive test (before vaccine)	<5	-	0	-	0	-	<5	-
0 day: first dose/positive test (before vaccine)	0	-	0	-	0	-	<5	-
1-7 days: first dose/positive test (before vaccine)	6	4.11 (1.68, 10.08)	0	-	<5	-	0	-
8-14 days: first dose/positive test (before vaccine)	6	4.33 (1.77, 10.58)	0	-	0	-	0	-
15-21 days: first dose/positive test (before vaccine)	<5	-	0	-	0	-	<5	-
22-28 days: first dose/positive test (before vaccine)	<5	-	0	-	0	-	<5	-
29-42 days: first dose/positive test (before vaccine)	<5	-	0	-	0	-	5	6.25 (2.30, 17.00)
-28-1 days: second dose/positive test (after vaccine)	<5	-	0	-	0	-	<5	-
0 day: second dose/positive test (after vaccine)	0	-	0	-	0	-	0	-
1-7 days: second dose/positive test (after vaccine)	8	8.65 (3.97, 18.87)	0	-	0	-	<5	-
8-14 days: second dose/positive test (after vaccine)	<5	-	0	-	0	-	<5	-

15-21 days: second dose/positive test (after vaccine)	<5	-	0	-	0	-	0	-
22-28 days: second dose/positive test (after vaccine)	<5	-	0	-	0	-	<5	-
29-42 days: second dose/positive test (after vaccine)	<5	-	0	-	0	-	<5	-
-28-1 days: third dose	<5	-	0	-	0	-		
0 day: third dose	0	-	0	-	0	-		
1-7 days: third dose	0	-	<5	-	0	-		
8-14 days: third dose	<5	-	<5	-	0	-		
15-21 days: third dose	0	-	0	-	0	-		
22-28 days: third dose	0	-	0	-	0	-		
29-42 days: third dose	0	-	0	-	0	-		
**Immune or idiopathic thrombocytopenia**								
baseline	568	1.00 (1.00, 1.00)	16	1.00 (1.00, 1.00)	17	1.00 (1.00, 1.00)	399	1.00 (1.00, 1.00)
-28-1 days: first dose/positive test (before vaccine)	19	0.50 (0.31, 0.79)	0	-	0	-	24	1.77 (1.15, 2.70)
0 day: first dose/positive test (before vaccine)	<5	-	0	-	0	-	15	29.89 (17.66, 50.58)
1-7 days: first dose/positive test (before vaccine)	<5	-	0	-	0	-	10	2.86 (1.52, 5.41)
8-14 days: first dose/positive test (before vaccine)	7	0.77 (0.36, 1.63)	0	-	0	-	<5	-
15-21 days: first dose/positive test (before vaccine)	13	1.44 (0.83, 2.53)	0	-	0	-	<5	-
22-28 days: first dose/positive test (before vaccine)	5	0.56 (0.23, 1.36)	0	-	<5	-	<5	-
29-42 days: first dose/positive test (before vaccine)	12	0.69 (0.38, 1.23)	0	-	0	-	19	2.80 (1.75, 4.49)
-28-1 days: second dose/positive test (after vaccine)	15	0.67 (0.40, 1.13)	0	-	0	-	8	1.16 (0.57, 2.39)
0 day: second dose/positive test (after vaccine)	<5	-	0	-	0	-	<5	-
1-7 days: second dose/positive test (after vaccine)	7	1.11 (0.52, 2.36)	0	-	0	-	<5	-
8-14 days: second dose/positive test (after vaccine)	<5	-	0	-	<5	-	<5	-
15-21 days: second dose/positive test (after vaccine)	<5	-	0	-	0	-	5	2.85 (1.16, 7.00)
22-28 days: second dose/positive test (after vaccine)	<5	-	0	-	0	-	<5	-
29-42 days: second dose/positive test (after vaccine)	14	1.17 (0.68, 2.02)	0	-	0	-	6	1.71 (0.75, 3.90)
-28-1 days: third dose	6	0.59 (0.26, 1.34)	0	-	0	-		
0 day: third dose	0	-	0	-	0	-		
1-7 days: third dose	<5	-	0	-	0	-		
8-14 days: third dose	<5	-	0	-	0	-		
15-21 days: third dose	<5	-	0	-	0	-		
22-28 days: third dose	<5	-	0	-	0	-		
29-42 days: third dose	<5	-	<5	-	0	-		
**Epilepsy**								
baseline	2338	1.00 (1.00, 1.00)	90	1.00 (1.00, 1.00)	74	1.00 (1.00, 1.00)	1375	1.00 (1.00, 1.00)
-28-1 days: first dose/positive test (before vaccine)	128	0.94 (0.78, 1.13)	0	-	<5	-	32	0.80 (0.56, 1.14)
0 day: first dose/positive test (before vaccine)	<5	-	0	-	0	-	36	25.18 (17.99, 35.23)
1-7 days: first dose/positive test (before vaccine)	36	1.05 (0.75, 1.46)	0	-	<5	-	22	2.21 (1.44, 3.38)
8-14 days: first dose/positive test (before vaccine)	36	1.04 (0.75, 1.45)	0	-	<5	-	8	0.81 (0.40, 1.63)
15-21 days: first dose/positive test (before vaccine)	44	1.23 (0.91, 1.67)	0	-	5	3.70 (1.48, 9.22)	13	1.31 (0.76, 2.27)
22-28 days: first dose/positive test (before vaccine)	34	0.96 (0.68, 1.35)	0	-	<5	-	12	1.20 (0.68, 2.12)
29-42 days: first dose/positive test (before vaccine)	65	0.93 (0.72, 1.20)	0	-	<5	-	22	1.11 (0.72, 1.69)
-28-1 days: second dose/positive test (after vaccine)	69	0.75 (0.59, 0.96)	<5	-	<5	-	27	0.97 (0.66, 1.43)

0 day: second dose/positive test (after vaccine)	<5	-	0	-	<5	-	7	6.96 (3.29, 14.71)
1-7 days: second dose/positive test (after vaccine)	32	1.15 (0.81, 1.64)	0	-	<5	-	13	1.86 (1.07, 3.23)
8-14 days: second dose/positive test (after vaccine)	27	0.97 (0.66, 1.42)	0	-	0	-	9	1.28 (0.66, 2.49)
15-21 days: second dose/positive test (after vaccine)	35	1.27 (0.91, 1.78)	0	-	<5	-	7	1.00 (0.47, 2.12)
22-28 days: second dose/positive test (after vaccine)	32	1.18 (0.83, 1.67)	0	-	0	-	7	1.01 (0.48, 2.14)
29-42 days: second dose/positive test (after vaccine)	64	1.20 (0.93, 1.54)	<5	-	5	1.96 (0.78, 4.88)	18	1.32 (0.82, 2.11)
-28-1 days: third dose	38	0.89 (0.64, 1.24)	<5	-	0	-		
0 day: third dose	0	-	0	-	0	-		
1-7 days: third dose	10	0.98 (0.53, 1.84)	0	-	0	-		
8-14 days: third dose	5	0.51 (0.21, 1.24)	<5	-	0	-		
15-21 days: third dose	8	0.85 (0.42, 1.71)	<5	-	0	-		
22-28 days: third dose	12	1.28 (0.72, 2.28)	<5	-	0	-		
29-42 days: third dose	13	0.70 (0.40, 1.21)	<5	-	0	-		
**Acute pancreatitis**								
baseline	207	1.00 (1.00, 1.00)	5	1.00 (1.00, 1.00)	<5	-	140	1.00 (1.00, 1.00)
-28-1 days: first dose/positive test (before vaccine)	5	0.35 (0.14, 0.87)	0	-	0	-	6	1.43 (0.62, 3.33)
0 day: first dose/positive test (before vaccine)	0	-	0	-	0	-	<5	-
1-7 days: first dose/positive test (before vaccine)	8	2.21 (1.07, 4.55)	0	-	0	-	0	-
8-14 days: first dose/positive test (before vaccine)	<5	-	0	-	0	-	<5	-
15-21 days: first dose/positive test (before vaccine)	<5	-	0	-	0	-	<5	-
22-28 days: first dose/positive test (before vaccine)	7	2.05 (0.95, 4.45)	0	-	<5	-	0	-
29-42 days: first dose/positive test (before vaccine)	6	0.81 (0.35, 1.87)	0	-	0	-	<5	-
-28-1 days: second dose/positive test (after vaccine)	6	0.63 (0.27, 1.44)	0	-	0	-	<5	-
0 day: second dose/positive test (after vaccine)	0	-	0	-	0	-	<5	-
1-7 days: second dose/positive test (after vaccine)	<5	-	0	-	0	-	<5	-
8-14 days: second dose/positive test (after vaccine)	0	-	0	-	0	-	0	-
15-21 days: second dose/positive test (after vaccine)	<5	-	0	-	0	-	0	-
22-28 days: second dose/positive test (after vaccine)	<5	-	0	-	0	-	<5	-
29-42 days: second dose/positive test (after vaccine)	<5	-	0	-	0	-	0	-
-28-1 days: third dose	<5	-	0	-	0	-		
0 day: third dose	0	-	0	-	0	-		
1-7 days: third dose	0	-	0	-	0	-		
8-14 days: third dose	<5	-	0	-	0	-		
15-21 days: third dose	<5	-	0	-	0	-		
22-28 days: third dose	<5	-	0	-	0	-		
29-42 days: third dose	<5	-	0	-	0	-		
**Myositis**								
baseline	71	1.00 (1.00, 1.00)	0	-	0	-	48	1.00 (1.00, 1.00)
-28-1 days: first dose/positive test (before vaccine)	<5	-	0	-	0	-	<5	-
0 day: first dose/positive test (before vaccine)	0	-	0	-	0	-	<5	-
1-7 days: first dose/positive test (before vaccine)	<5	-	0	-	0	-	<5	-
8-14 days: first dose/positive test (before vaccine)	<5	-	0	-	0	-	0	-
15-21 days: first dose/positive test (before vaccine)	<5	-	0	-	0	-	0	-

22-28 days: first dose/positive test (before vaccine)	<5	-	0	-	0	-	0	-
29-42 days: first dose/positive test (before vaccine)	<5	-	0	-	0	-	0	-
-28-1 days: second dose/positive test (after vaccine)	<5	-	0	-	0	-	<5	-
0 day: second dose/positive test (after vaccine)	0	-	0	-	0	-	0	-
1-7 days: second dose/positive test (after vaccine)	<5	-	0	-	0	-	0	-
8-14 days: second dose/positive test (after vaccine)	0	-	0	-	0	-	0	-
15-21 days: second dose/positive test (after vaccine)	0	-	0	-	0	-	0	-
22-28 days: second dose/positive test (after vaccine)	0	-	0	-	0	-	0	-
29-42 days: second dose/positive test (after vaccine)	<5	-	0	-	<5	-	0	-
-28-1 days: third dose	<5	-	0	-	0	-		
0 day: third dose	0	-	0	-	0	-		
1-7 days: third dose	<5	-	0	-	0	-		
8-14 days: third dose	0	-	0	-	0	-		
15-21 days: third dose	0	-	0	-	0	-		
22-28 days: third dose	0	-	0	-	0	-		
29-42 days: third dose	0	-	0	-	0	-		
**Acute disseminated encephalomyelitis**								
baseline	94	1.00 (1.00, 1.00)	<5	-	0	-	74	1.00 (1.00, 1.00)
-28-1 days: first dose/positive test (before vaccine)	5	0.89 (0.35, 2.26)	0	-	0	-	9	3.11 (1.51, 6.40)
0 day: first dose/positive test (before vaccine)	0	-	0	-	0	-	5	46.95 (18.45, 119.50)
1-7 days: first dose/positive test (before vaccine)	0	-	0	-	0	-	<5	-
8-14 days: first dose/positive test (before vaccine)	<5	-	0	-	0	-	<5	-
15-21 days: first dose/positive test (before vaccine)	<5	-	0	-	0	-	0	-
22-28 days: first dose/positive test (before vaccine)	<5	-	0	-	0	-	<5	-
29-42 days: first dose/positive test (before vaccine)	<5	-	0	-	0	-	<5	-
-28-1 days: second dose/positive test (after vaccine)	5	1.44 (0.56, 3.74)	0	-	0	-	0	-
0 day: second dose/positive test (after vaccine)	0	-	0	-	0	-	<5	-
1-7 days: second dose/positive test (after vaccine)	<5	-	0	-	0	-	<5	-
8-14 days: second dose/positive test (after vaccine)	0	-	0	-	0	-	<5	-
15-21 days: second dose/positive test (after vaccine)	<5	-	0	-	0	-	0	-
22-28 days: second dose/positive test (after vaccine)	<5	-	0	-	0	-	0	-
29-42 days: second dose/positive test (after vaccine)	<5	-	0	-	0	-	0	-
-28-1 days: third dose	0	-	0	-	0	-		
0 day: third dose	0	-	0	-	0	-		
1-7 days: third dose	0	-	0	-	0	-		
8-14 days: third dose	0	-	0	-	0	-		
15-21 days: third dose	0	-	0	-	0	-		
22-28 days: third dose	0	-	0	-	0	-		
29-42 days: third dose	0	-	0	-	0	-		
**Demyelinating disease**								
baseline	109	1.00 (1.00, 1.00)	6	1.00 (1.00, 1.00)	<5	-	77	1.00 (1.00, 1.00)
-28-1 days: first dose/positive test (before vaccine)	7	1.17 (0.53, 2.61)	0	-	0	-	<5	-
0 day: first dose/positive test (before vaccine)	0	-	0	-	0	-	<5	-

1-7 days: first dose/positive test (before vaccine)	<5	-	0	-	0	-	<5	-
8-14 days: first dose/positive test (before vaccine)	<5	-	0	-	0	-	0	-
15-21 days: first dose/positive test (before vaccine)	<5	-	0	-	0	-	<5	-
22-28 days: first dose/positive test (before vaccine)	<5	-	0	-	0	-	0	-
29-42 days: first dose/positive test (before vaccine)	<5	-	0	-	0	-	<5	-
-28-1 days: second dose/positive test (after vaccine)	<5	-	0	-	0	-	<5	-
0 day: second dose/positive test (after vaccine)	0	-	0	-	0	-	0	-
1-7 days: second dose/positive test (after vaccine)	<5	-	0	-	0	-	0	-
8-14 days: second dose/positive test (after vaccine)	<5	-	0	-	0	-	<5	-
15-21 days: second dose/positive test (after vaccine)	0	-	0	-	0	-	0	-
22-28 days: second dose/positive test (after vaccine)	<5	-	0	-	0	-	0	-
29-42 days: second dose/positive test (after vaccine)	<5	-	0	-	0	-	0	-
-28-1 days: third dose	0	-	0	-	0	-		
0 day: third dose	0	-	0	-	0	-		
1-7 days: third dose	0	-	0	-	0	-		
8-14 days: third dose	0	-	0	-	0	-		
15-21 days: third dose	<5	-	0	-	0	-		
22-28 days: third dose	0	-	0	-	0	-		
29-42 days: third dose	0	-	0	-	0	-		
**Appendicitis**								
baseline	5527	1.00 (1.00, 1.00)	121	1.00 (1.00, 1.00)	16	1.00 (1.00, 1.00)	3665	1.00 (1.00, 1.00)
-28-1 days: first dose/positive test (before vaccine)	214	0.65 (0.56, 0.74)	0	-	<5	-	88	0.67 (0.54, 0.83)
0 day: first dose/positive test (before vaccine)	<5	-	0	-	0	-	78	16.47 (13.12, 20.67)
1-7 days: first dose/positive test (before vaccine)	87	1.03 (0.83, 1.28)	0	-	0	-	28	0.86 (0.59, 1.24)
8-14 days: first dose/positive test (before vaccine)	75	0.89 (0.71, 1.12)	0	-	0	-	33	1.02 (0.72, 1.44)
15-21 days: first dose/positive test (before vaccine)	70	0.82 (0.65, 1.04)	0	-	<5	-	39	1.20 (0.88, 1.66)
22-28 days: first dose/positive test (before vaccine)	83	0.96 (0.77, 1.20)	0	-	<5	-	35	1.08 (0.77, 1.51)
29-42 days: first dose/positive test (before vaccine)	146	0.85 (0.71, 1.00)	0	-	<5	-	74	1.13 (0.90, 1.43)
-28-1 days: second dose/positive test (after vaccine)	156	0.64 (0.54, 0.76)	0	-	<5	-	49	0.79 (0.59, 1.05)
0 day: second dose/positive test (after vaccine)	<5	-	0	-	0	-	27	11.51 (7.84, 16.90)
1-7 days: second dose/positive test (after vaccine)	76	1.20 (0.95, 1.51)	0	-	0	-	11	0.68 (0.38, 1.23)
8-14 days: second dose/positive test (after vaccine)	51	0.82 (0.62, 1.09)	0	-	<5	-	15	0.94 (0.56, 1.57)
15-21 days: second dose/positive test (after vaccine)	52	0.84 (0.64, 1.11)	0	-	<5	-	19	1.19 (0.75, 1.88)
22-28 days: second dose/positive test (after vaccine)	44	0.71 (0.53, 0.96)	0	-	<5	-	16	1.01 (0.61, 1.65)
29-42 days: second dose/positive test (after vaccine)	107	0.89 (0.73, 1.08)	0	-	<5	-	36	1.13 (0.81, 1.58)
-28-1 days: third dose	36	0.77 (0.55, 1.08)	<5	-	0	-		
0 day: third dose	0	-	0	-	0	-		
1-7 days: third dose	11	0.99 (0.54, 1.79)	0	-	0	-		
8-14 days: third dose	8	0.74 (0.37, 1.48)	<5	-	0	-		
15-21 days: third dose	10	0.96 (0.51, 1.78)	<5	-	0	-		
22-28 days: third dose	10	0.97 (0.52, 1.81)	<5	-	0	-		
29-42 days: third dose	25	1.27 (0.85, 1.89)	<5	-	0	-		
**Guillain-Barre Syndrome**								

baseline	22	-	<5	-	0	-	13	
-28-1 days: first dose/positive test (before vaccine)	0	-	0	-	0	-	0	
0 day: first dose/positive test (before vaccine)	0	-	0	-	0	-	0	
1-7 days: first dose/positive test (before vaccine)	0	-	0	-	0	-	<5	
8-14 days: first dose/positive test (before vaccine)	0	-	0	-	0	-	<5	
15-21 days: first dose/positive test (before vaccine)	<5	-	0	-	0	-	<5	
22-28 days: first dose/positive test (before vaccine)	0	-	0	-	0	-	0	
29-42 days: first dose/positive test (before vaccine)	<5	-	0	-	0	-	<5	
-28-1 days: second dose/positive test (after vaccine)	0	-	0	-	0	-	0	
0 day: second dose/positive test (after vaccine)	0	-	0	-	0	-	<5	
1-7 days: second dose/positive test (after vaccine)	0	-	0	-	0	-	0	
8-14 days: second dose/positive test (after vaccine)	0	-	0	-	0	-	<5	
15-21 days: second dose/positive test (after vaccine)	0	-	0	-	0	-	0	
22-28 days: second dose/positive test (after vaccine)	<5	-	0	-	0	-	0	
29-42 days: second dose/positive test (after vaccine)	0	-	0	-	0	-	0	
-28-1 days: third dose	0	-	0	-	0	-		
0 day: third dose	0	-	0	-	0	-		
1-7 days: third dose	0	-	0	-	0	-		
8-14 days: third dose	0	-	0	-	0	-		
15-21 days: third dose	0	-	0	-	0	-		
22-28 days: third dose	0	-	0	-	0	-		
29-42 days: third dose	0	-	0	-	0	-		
**Angioedema**								
baseline	226	1.00 (1.00, 1.00)	5	1.00 (1.00, 1.00)	<5	-	164	1.00 (1.00, 1.00)
-28-1 days: first dose/positive test (before vaccine)	9	0.77 (0.38, 1.53)	0	-	<5	-	<5	-
0 day: first dose/positive test (before vaccine)	<5	-	0	-	0	-	0	-
1-7 days: first dose/positive test (before vaccine)	<5	-	0	-	0	-	<5	-
8-14 days: first dose/positive test (before vaccine)	<5	-	0	-	0	-	<5	-
15-21 days: first dose/positive test (before vaccine)	<5	-	0	-	0	-	0	-
22-28 days: first dose/positive test (before vaccine)	5	1.66 (0.67, 4.13)	0	-	0	-	0	-
29-42 days: first dose/positive test (before vaccine)	11	1.96 (1.04, 3.70)	0	-	<5	-	5	1.92 (0.77, 4.77)
-28-1 days: second dose/positive test (after vaccine)	8	1.06 (0.51, 2.21)	0	-	0	-	<5	-
0 day: second dose/positive test (after vaccine)	0	-	0	-	0	-	<5	-
1-7 days: second dose/positive test (after vaccine)	<5	-	0	-	<5	-	<5	-
8-14 days: second dose/positive test (after vaccine)	<5	-	0	-	0	-	0	-
15-21 days: second dose/positive test (after vaccine)	<5	-	0	-	0	-	0	-
22-28 days: second dose/positive test (after vaccine)	<5	-	0	-	0	-	0	-
29-42 days: second dose/positive test (after vaccine)	5	1.14 (0.46, 2.82)	0	-	0	-	0	-
-28-1 days: third dose	<5	-	0	-	0	-		
0 day: third dose	<5	-	<5	-	0	-		
1-7 days: third dose	<5	-	<5	-	0	-		
8-14 days: third dose	0	-	0	-	0	-		
15-21 days: third dose	0	-	0	-	0	-		

22-28 days: third dose	0	-	0	-	0	-		
29-42 days: third dose	0	-	0	-	0	-		
**Anaphylaxis**								
baseline	398	1.00 (1.00, 1.00)	10	1.00 (1.00, 1.00)	<5	-	265	1.00 (1.00, 1.00)
-28-1 days: first dose/positive test (before vaccine)	18	0.85 (0.52, 1.39)	0	-	0	-	10	1.14 (0.60, 2.16)
0 day: first dose/positive test (before vaccine)	<5	-	0	-	0	-	<5	-
1-7 days: first dose/positive test (before vaccine)	5	0.82 (0.33, 2.01)	0	-	0	-	0	-
8-14 days: first dose/positive test (before vaccine)	7	1.23 (0.57, 2.63)	0	-	0	-	5	2.35 (0.96, 5.75)
15-21 days: first dose/positive test (before vaccine)	12	2.21 (1.22, 4.01)	0	-	0	-	<5	-
22-28 days: first dose/positive test (before vaccine)	7	1.28 (0.60, 2.74)	0	-	0	-	<5	-
29-42 days: first dose/positive test (before vaccine)	13	1.21 (0.68, 2.14)	0	-	0	-	8	1.87 (0.92, 3.83)
-28-1 days: second dose/positive test (after vaccine)	10	0.63 (0.33, 1.21)	0	-	0	-	<5	-
0 day: second dose/positive test (after vaccine)	<5	-	0	-	0	-	7	48.30 (21.85, 106.77)
1-7 days: second dose/positive test (after vaccine)	<5	-	0	-	0	-	0	-
8-14 days: second dose/positive test (after vaccine)	<5	-	0	-	0	-	<5	-
15-21 days: second dose/positive test (after vaccine)	5	1.22 (0.50, 2.99)	0	-	0	-	<5	-
22-28 days: second dose/positive test (after vaccine)	<5	-	0	-	0	-	0	-
29-42 days: second dose/positive test (after vaccine)	6	0.71 (0.31, 1.60)	0	-	0	-	<5	-
-28-1 days: third dose	5	1.33 (0.53, 3.33)	<5	-	0	-		
0 day: third dose	0	-	0	-	0	-		
1-7 days: third dose	<5	-	0	-	0	-		
8-14 days: third dose	<5	-	0	-	0	-		
15-21 days: third dose	<5	-	0	-	0	-		
22-28 days: third dose	0	-	<5	-	0	-		
29-42 days: third dose	<5	-	0	-	0	-		

Supplementary Table 9. Incidence rate ratios and estimated number of excess events per million for individual outcomes in the 1-42 days following vaccination or a SARS-CoV-2 positive test (before or after vaccination) compared to baseline period in young adults aged 18-24 years, and stratified by sex. Cells with a hyphen are suppressed; N – number of events; IRR – incidence rate ratio; CI – confidence interval.

	**BNT162b2 vaccine**			**mRNA-1273 vaccine**			**ChAdOX1 vaccine**			**Positive SARS-CoV-2 test**		
	N	IRR (95% CI)	Excess events (95% CI)	N	IRR (95% CI)	Excess events (95% CI)	N	IRR (95% CI)	Excess events (95% CI)	N	IRR (95% CI)	Excess events (95% CI)
**Total population**												
**Multisystem inflammatory syndrome**												
First dose/positive test (before vaccine)	<5	-	-	0	-	-	<5	-	-	5	5.72 (1.86, 17.59)	6 (4, 7)
Second dose/positive test (after vaccine)	<5	-	-	0	-	-	<5	-	-	<5	-	-
Third dose	0	-	-	0	-	-	0	-	-			
**Myocarditis**												
First dose/positive test (before vaccine)	26	1.04 (0.66, 1.63)	0	9	2.53 (1.12, 5.71)	15 (3, 21)	5	1.54 (0.59, 4.03)	0	19	3.25 (1.95, 5.41)	20 (14, 24)
Second dose/positive test (after vaccine)	49	2.25 (1.59, 3.19)	10 (6, 12)	29	12.32 (6.81, 22.28)	86 (80, 89)	9	1.80 (0.85, 3.81)	0	<5	-	-
Third dose	27	2.84 (1.78, 4.51)	12 (8, 14)	13	4.15 (2.11, 8.14)	13 (9,15)	0	-	-			
**Immune or idiopathic thrombocytopenia**												
First dose/positive test (before vaccine)	58	0.88 (0.67, 1.17)	0	<5	-	-	40	1.47 (1.02, 2.12)	24 (2, 39)	58	3.88 (2.88, 5.21)	66 (58, 72)
Second dose/positive test (after vaccine)	55	0.99 (0.74, 1.32)	0	6	0.96 (0.42, 2.24)	0	29	1.09 (0.72, 1.63)	0	15	2.15 (1.27, 3.64)	9 (4, 12)
Third dose	32	0.76 (0.51, 1.12)	0	12	1.02 (0.56, 1.87)	0	0	-	-			
**Epilepsy**												
First dose/positive test (before vaccine)	168	0.88 (0.75, 1.04)	0	7	0.62 (0.29, 1.33)	0	215	1.02 (0.86, 1.20)	0	83	1.68 (1.33, 2.11)	52 (32, 67)
Second dose/positive test (after vaccine)	156	0.91 (0.77, 1.07)	0	6	0.66 (0.29, 1.50)	0	210	0.95 (0.81, 1.12)	0	42	1.50 (1.10, 2.04)	15 (4, 24)
Third dose	172	1.00 (0.85, 1.19)	0	40	0.79 (0.57, 1.11)	0	<5	-	-			
**Acute pancreatitis**												
First dose/positive test (before vaccine)	70	1.08 (0.83, 1.41)	0	8	1.38 (0.65, 2.92)	0	10	0.45 (0.24, 0.87)	0	30	1.82 (1.24, 2.69)	21 (9, 29)
Second dose/positive test (after vaccine)	63	1.13 (0.85, 1.49)	0	5	1.25 (0.49, 3.15)	0	26	1.15 (0.75, 1.76)	0	6	0.79 (0.35, 1.79)	0
Third dose	39	1.22 (0.86, 1.73)	0	13	0.88 (0.48, 1.61)	0	<5	-	-			
**Myositis**												

First dose/positive test (before vaccine)	<5	-	-	0	-	-	<5	-	-	<5	-	-
Second dose/positive test (after vaccine)	<5	-	-	<5	-	-	<5	-	-	<5	-	-
Third dose	<5	-	-	0	-	-	0	-	-			
**Acute disseminated encephalomyelitis**												
First dose/positive test (before vaccine)	12	1.77 (0.93, 3.39)	0	<5	-	-	<5	-	-	<5	-	-
Second dose/positive test (after vaccine)	8	1.20 (0.56, 2.57)	0	<5	-	-	6	2.13 (0.82, 5.53)	0	<5	-	-
Third dose	5	1.00 (0.37, 2.66)	0	<5	-	-	0	-	-			
**Demyelinating disease**												
First dose/positive test (before vaccine)	11	0.57 (0.30, 1.08)	0	0	-	-	7	1.03 (0.45, 2.37)	0	<5	-	-
Second dose/positive test (after vaccine)	11	0.69 (0.37, 1.30)	0	<5	-	-	5	0.68 (0.26, 1.76)	0	<5	-	-
Third dose	11	0.97 (0.51, 1.88)	0	<5	-	-	0	-	-			
**Anaphylaxis**												
First dose/positive test (before vaccine)	40	1.02 (0.72, 1.45)	0	<5	-	-	19	1.29 (0.76, 2.16)	0	10	1.09 (0.57, 2.09)	0
Second dose/positive test (after vaccine)	30	0.98 (0.66, 1.44)	0	<5	-	-	20	1.26 (0.76, 2.09)	0	<5	-	-
Third dose	16	1.00 (0.57, 1.74)	0	6	1.06 (0.45, 2.48)	0	0	-	-			
**Appendicitis**												
First dose/positive test (before vaccine)	470	0.99 (0.89, 1.10)	0	60	1.16 (0.88, 1.53)	0	111	1.10 (0.89, 1.35)	0	117	1.22 (1.01, 1.48)	33 (2, 59)
Second dose/positive test (after vaccine)	382	0.92 (0.82, 1.03)	0	48	1.12 (0.83, 1.51)	0	94	1.03 (0.83, 1.28)	0	57	1.23 (0.94, 1.61)	0
Third dose	207	0.99 (0.85, 1.16)	0	93	0.98 (0.79, 1.22)	0	0	-	-			
**Guillain-Barre Syndrome**												
First dose/positive test (before vaccine)	<5	-	-	<5	-	-	<5	-	-	<5	-	-
Second dose/positive test (after vaccine)	5	1.39 (0.50, 3.86)	0	0	-	-	0	-	-	0	-	-
Third dose	<5	-	-	<5	-	-	0	-	-			
**Angioedema**												
First dose/positive test (before vaccine)	28	0.87 (0.57, 1.32)	0	<5	-	-	17	1.21 (0.70, 2.10)	0	7	0.87 (0.40, 1.88)	0
Second dose/positive test (after vaccine)	25	1.01 (0.65, 1.56)	0	<5	-	-	13	0.98 (0.53, 1.80)	0	6	2.15 (0.94, 4.94)	

Third dose	6	0.51 (0.22, 1.17)	0	<5	-	-	0	-	-			
**Females**												
**Multisystem inflammatory syndrome**												
First dose/positive test (before vaccine)	<5	-	-	0	-	-	<5	-	-	<5	-	-
Second dose/positive test (after vaccine)	<5	-	-	0	-	-	<5	-	-	<5	-	-
Third dose	0	-	-	0	-	-	0	-	-			
**Myocarditis**												
First dose/positive test (before vaccine)	7	1.17 (0.47, 2.95)	0	<5	-	-	<5	-	-	6	6.50 (2.36, 17.86)	18 (12, 20)
Second dose/positive test (after vaccine)	5	1.28 (0.48, 3.36)	0	<5	-	-	0	-	-	<5	-	-
Third dose	<5	-	-	<5	-	-	0	-	-			
**Immune or idiopathic thrombocytopenia**												
First dose/positive test (before vaccine)	36	0.90 (0.63, 1.29)	0	<5	-	-	23	1.27 (0.79, 2.04)	0	33	3.24 (2.20, 4.77)	80 (63, 91)
Second dose/positive test (after vaccine)	39	1.17 (0.83, 1.65)	0	<5	-	-	17	1.04 (0.62, 1.76)	0	6	1.25 (0.55, 2.84)	0
Third dose	21	0.78 (0.48, 1.27)	0	5	0.60 (0.24, 1.50)	0	0	-	-			
**Epilepsy**												
First dose/positive test (before vaccine)	91	0.82 (0.66, 1.02)	0	<5	-	-	122	0.98 (0.79, 1.21)	0	56	1.79 (1.35, 2.38)	87 (51, 113)
Second dose/positive test (after vaccine)	81	0.82 (0.65, 1.04)	0	<5	-	-	113	0.95 (0.76, 1.18)	0	22	1.18 (0.77, 1.81)	0
Third dose	88	0.85 (0.67, 1.07)	0	22	0.73 (0.46, 1.14)	0	<5	-	-			
**Acute pancreatitis**												
First dose/positive test (before vaccine)	45	1.06 (0.76, 1.47)	0	7	1.91 (0.84, 4.33)	0	6	0.38 (0.17, 0.89)	0	21	1.91 (1.20, 3.03)	35 (12, 49)
Second dose/positive test (after vaccine)	38	1.07 (0.75, 1.52)	0	<5	-	-	17	1.07 (0.63, 1.82)	0	5	0.88 (0.36, 2.15)	
Third dose	27	1.23 (0.81, 1.88)		8	0.85 (0.40, 1.77)	0	<5					
**Myositis**												
First dose/positive test (before vaccine)	<5	-	-	0	-	-	0	-	-	0	-	-
Second dose/positive test (after vaccine)	0	-	-	0	-	-	<5	-	-	<5	-	-
Third dose	<5	-	-	0	-	-	0	-	-			
**Acute disseminated encephalomyelitis**												

First dose/positive test (before vaccine)	<5	-	-	0	-	-	<5	-	-	<5	-	-
Second dose/positive test (after vaccine)	<5	-	-	0	-	-	<5	-	-	0	-	-
Third dose	<5	-	-	<5	-	-	0	-	-			
**Demyelinating disease**												
First dose/positive test (before vaccine)	8	0.56 (0.27, 1.17)	0	0	-	-	6	1.05 (0.42, 2.61)	0	<5	-	-
Second dose/positive test (after vaccine)	8	0.62 (0.30, 1.30)	0	<5	-	-	5	0.83 (0.31, 2.21)	0	<5	-	-
Third dose	8	0.94 (0.44, 2.03)	0	<5	-	-	0	-	-			
**Anaphylaxis**												
First dose/positive test (before vaccine)	24	1.00 (0.63, 1.57)	0	<5	-	-	11	1.06 (0.54, 2.07)	0	<5	-	-
Second dose/positive test (after vaccine)	19	0.96 (0.59, 1.57)	0	<5	-	-	14	1.36 (0.74, 2.51)	0	<5	-	-
Third dose	9	0.87 (0.41, 1.84)	0	5	1.63 (0.63, 4.27)	0	0	-	-			
**Appendicitis**												
First dose/positive test (before vaccine)	216	0.97 (0.83, 1.13)	0	23	0.99 (0.64, 1.54)	0	69	1.09 (0.84, 1.41)	0	54	1.16 (0.87, 1.53)	0
Second dose/positive test (after vaccine)	187	0.94 (0.80, 1.11)	0	27	1.29 (0.86, 1.94)	0	66	1.21 (0.93, 1.58)	0	27	0.99 (0.67, 1.45)	0
Third dose	98	0.82 (0.66, 1.03)	0	48	1.01 (0.74, 1.38)	0	0	-	-			
**Guillain-Barre Syndrome**												
First dose/positive test (before vaccine)	<5	-	-	<5	-	-	0	-	-	<5	-	-
Second dose/positive test (after vaccine)	<5	-	-	0	-	-	0	-	-	0	-	-
Third dose	<5	-	-	<5	-	-	0	-	-			
**Angioedema**												
First dose/positive test (before vaccine)	19	0.80 (0.48, 1.33)	0	<5	-	-	13	1.25 (0.66, 2.35)	0	5	0.82 (0.33, 2.03)	0
Second dose/positive test (after vaccine)	22	1.17 (0.73, 1.88)	0	<5	-	-	10	1.01 (0.51, 2.02)	0	5	2.18 (0.88, 5.42)	0
Third dose	<5	-	-	<5	-	-	0	-	-			
**Males**												
**Multisystem inflammatory syndrome**												
First dose/positive test (before vaccine)	0	-	-	0	-	-	0	-	-	<5	-	-

Second dose/positive test (after vaccine)	0	-	-	0	-	-	0	-	-	0	-	-
Third dose	0	-	-	0	-	-	0	-	-			
**Myocarditis**												
First dose/positive test (before vaccine)	19	1.01 (0.60, 1.70)	0	8	3.57 (1.52, 8.39)	39 (19, 48)	<5	-	-	13	2.71 (1.48, 4.97)	32 (16, 40)
Second dose/positive test (after vaccine)	44	2.50 (1.71, 3.66)	23 (16, 28)	25	13.97 (7.23, 27.02)	173 (160, 179)	9	2.70 (1.23, 5.90)	29 (9, 38)	<5	-	-
Third dose	24	3.24 (1.97, 5.35)	28 (20, 33)	10	4.74 (2.23, 10.08)	26 (18, 29)	0	-	-			
**Immune or idiopathic thrombocytopenia**												
First dose/positive test (before vaccine)	22	0.85 (0.53, 1.35)	0	<5	-	-	17	1.87 (1.05, 3.33)	38 (4, 58)	25	5.18 (3.23, 8.28)	78 (67, 85)
Second dose/positive test (after vaccine)	16	0.70 (0.41, 1.20)	0	<5	-	-	12	1.18 (0.62, 2.24)	0	9	4.40 (2.18, 8.91)	22 (15, 25)
Third dose	11	0.73 (0.38, 1.42)	0	7	2.10 (0.92, 4.83)	0	0	-	-			
**Epilepsy**												
First dose/positive test (before vaccine)	77	0.96 (0.75, 1.22)	0	<5	-	-	93	1.08 (0.84, 1.40)	0	27	1.48 (0.99, 2.21)	0
Second dose/positive test (after vaccine)	75	1.01 (0.79, 1.29)	0	<5	-	-	97	0.96 (0.75, 1.21)	0	20	2.09 (1.33, 3.29)	32 (15, 43)
Third dose	84	1.24 (0.97, 1.58)	0	18	0.90 (0.55, 1.49)	0	<5	-	-			
**Acute pancreatitis**												
First dose/positive test (before vaccine)	25	1.15 (0.73, 1.83)	0	<5	-	-	<5	-	-	9	1.66 (0.82, 3.37)	0
Second dose/positive test (after vaccine)	25	1.22 (0.77, 1.92)	0	<5	-	-	9	1.34 (0.64, 2.79)	0	<5	-	-
Third dose	12	1.16 (0.61, 2.19)	0	5	0.92 (0.32, 2.63)	0	0	-	-			
**Myositis**												
First dose/positive test (before vaccine)	0	-	-	0	-	-	<5	-	-	<5	-	-
Second dose/positive test (after vaccine)	<5	-	-	<5	-	-	0	-	-	0	-	-
Third dose	0	-	-	0	-	-	0	-	-			
**Acute disseminated encephalomyelitis**												
First dose/positive test (before vaccine)	8	3.79 (1.59, 9.02)	5 (2, 6)	<5	-	-	<5	-	-	<5	-	-
Second dose/positive test (after vaccine)	<5	-	-	<5	-	-	<5	-	-	<5	-	-
Third dose	<5	-	-	0	-	-	0	-	-			
**Demyelinating disease**												

First dose/positive test (before vaccine)	<5	-	-	0	-	-	<5	-	-	<5	-	-
Second dose/positive test (after vaccine)	<5	-	-	<5	-	-	0	-	-	0	-	-
Third dose	<5	-	-	<5	-	-	0	-	-			
**Anaphylaxis**												
First dose/positive test (before vaccine)	16	1.10 (0.62, 1.96)	0	<5	-	-	8	1.81 (0.78, 4.20)	0	6	2.13 (0.88, 5.14)	0
Second dose/positive test (after vaccine)	11	1.03 (0.54, 1.98)	0	<5	-	-	6	1.10 (0.43, 2.79)	0	0	-	-
Third dose	7	1.16 (0.51, 2.65)	0	<5	-	-	0	-	-			
**Appendicitis**												
First dose/positive test (before vaccine)	254	1.01 (0.87, 1.18)	0	37	1.30 (0.90, 1.86)	0	42	1.11 (0.80, 1.54)	0	63	1.29 (0.99, 1.67)	0
Second dose/positive test (after vaccine)	195	0.91 (0.78, 1.07)	0	21	0.96 (0.61, 1.50)	0	28	0.78 (0.53, 1.16)	0	30	1.57 (1.08, 2.26)	34 (7, 52)
Third dose	109	1.20 (0.97, 1.49)	0	45	0.96 (0.70, 1.31)	0	0	-	-			
**Guillain-Barre Syndrome**												
First dose/positive test (before vaccine)	<5	-	-	0	-	-	<5	-	-	0	-	-
Second dose/positive test (after vaccine)	<5	-	-	0	-	-	0	-	-	0	-	-
Third dose	0	-	-	<5	-	-	0	-	-			
**Angioedema**												
First dose/positive test (before vaccine)	9	1.04 (0.47, 2.29)	0	0	-	-	<5	-	-	<5	-	-
Second dose/positive test (after vaccine)	<5	-	-	0	-	-	<5	-	-	<5	-	-
Third dose	<5	-	-	0	-	-	0	-	-			

Supplementary Table 10. Characteristics of unvaccinated and vaccinated young people and those testing positive for SARS-CoV-2 aged 5-11, 12-17 and 18-24 years included in the QResearch primary care database in England between 8th December 2020 until 7th August 2022. Cells with <5 are suppressed. Data are presented as counts (column %).

	Unvaccinated	Dose 1			Dose 2			Dose 3			Positive SARS-CoV-2 test	Total
		BNT162b2	mRNA-1273	ChAdOX1	BNT162b2	mRNA-1273	ChAdOX1	BNT162b2	mRNA-1273	ChAdOX1		
**5-11 years**												
**Total N**	1,420,607	160,208	20	34	83,797	9	<5	244	0	0	418,680	1,580,869
**Mean age (SD)**	8.3 (2.1)	8.7 (2.0)	8.0 (1.9)	8.6 (1.6)	8.8 (1.9)	9.3 (1.3)	-	10.1 (1.7)	-	-	9.5 (1.9)	8.4 (2.0)
**Sex, N (%)**												
Female	693,571 (48.8)	77,910 (48.6)	13 (65.0)	19 (55.9)	40,637 (48.5)	<5	<5	101 (41.4)	0	0	207,170 (49.5)	771,513 (48.8)
Male	727,036 (51.2)	82,298 (51.4)	7 (35.0)	15 (44.1)	43,160 (51.5)	<5	<5	143 (58.6)	0	0	211,510 (50.5)	809,356 (51.2)
**Ethnicity, N (%)**												
White	707,439 (49.8)	90,627 (56.6)	10 (50.0)	18 (52.9)	48,235 (57.6)	7 (77.8)	<5	176 (72.1)	0	0	253,035 (60.4)	798,094 (50.5)
Indian	34,714 (2.4)	8,730 (5.4)	<5	<5	4,679 (5.6)	<5	<5	<5	0	0	10,603 (2.5)	43,444 (2.7)
Pakistani	40,461 (2.8)	2,469 (1.5)	<5	<5	1,031 (1.2)	<5	<5	<5	0	0	6,989 (1.7)	42,930 (2.7)
Bangladeshi	23,309 (1.6)	2,346 (1.5)	<5	<5	1,139 (1.4)	<5	<5	<5	0	0	3,582 (0.9)	25,655 (1.6)
Other Asian	26,374 (1.9)	3,444 (2.1)	<5	<5	1,709 (2.0)	<5	<5	<5	0	0	5,810 (1.4)	29,820 (1.9)
Black Caribbean	8,914 (0.6)	176 (0.1)	<5	<5	91 (0.1)	<5	<5	<5	0	0	1,396 (0.3)	9,090 (0.6)
Black African	46,541 (3.3)	1,977 (1.2)	<5	<5	909 (1.1)	<5	<5	<5	0	0	5,413 (1.3)	48,518 (3.1)
Chinese	7,250 (0.5)	1,627 (1.0)	<5	<5	956 (1.1)	<5	<5	<5	0	0	1,878 (0.4)	8,878 (0.6)
Other	94,749 (6.7)	7,371 (4.6)	<5	<5	3,817 (4.6)	<5	<5	10 (4.1)	0	0	20,213 (4.8)	102,123 (6.5)
Missing	430,856 (30.3)	41,441 (25.9)	6 (30.0)	14 (41.2)	21,231 (25.3)	<5	<5	48 (19.7)	0	0	109,761 (26.2)	472,317 (29.9)
**Positive SARS-CoV-2 test during study period, N (%)**												
None	1,071,114 (75.4)	91,044 (56.8)	14 (70.0)	17 (50.0)	46,595 (55.6)	<5	<5	130 (53.3)	0	0	0	1,162,189 (73.5)
Prior to first vaccine dose	349,493 (24.6)	67,610 (42.2)	6 (30.0)	16 (47.1)	36,380 (43.4)	5 (55.6)	0	94 (38.5)	0	0	417,125 (99.6)	417,125 (26.4)
After first vaccine dose	0	1,554 (1.0)	0	<5	822 (1.0)	0	0	20 (8.2)	0	0	1,555 (0.4)	1,555 (0.1)
**Vaccine doses received, N (%)**												
No vaccine	1,420,607 (100)	-	-	-	-	-	-	-	-	-	349,493 (83.5)	1,420,607 (89.9)
Dose 1	-	160,208 (100)	20 (100)	34 (100)	83,797 (100)	9 (100)	<5	244 (100)	0	0	69,187 (16.5)	160,262 (10.1)
Dose 2	-	83,804 (52.3)	<5	<5	83,797 (100)	9 (100)	<5	244 (100)	0	0	37,207 (8.9)	83,808 (5.3)
Dose 3	-	243 (0.2)	0	<5	243 (0.3)	0	<5	244 (100)	0	0	114 (<0.1)	244 (<0.1)
**Any comorbidity, N (%)**									0	0		
No	1,291,321 (90.9)	137,795 (86.0)	20 (100)	27 (79.4)	70,996 (84.7)	5 (55.6)	<5	92 (37.7)	0	0	367,519 (87.8)	1,429,163 (90.4)
Yes	129,286 (9.1)	22,413 (14.0)	0	7 (20.6)	12,801 (15.3)	<5	<5	152 (62.3)	0	0	51,161 (12.2)	151,706 (9.6)
**12-17 years**												

**Total N**	687,155	844,448	350	3,388	658,536	1,129	3,030	120,426	16,535	9	475,049	1,535,341
**Mean age (SD)**	14.4 (1.8)	14.6 (1.8)	15.9 (1.6)	16.6 (0.7)	14.7 (1.8)	16.5 (1.1)	16.6 (0.6)	16.0 (1.1)	17.0 (0.2)	16.4 (0.7)	15.5 (1.9)	15.2 (2.0)
**Sex, N (%)**												
Female	332,851 (48.4)	423,823 (50.2)	178 (50.9)	1,826 (53.9)	333,222 (50.6)	582 (51.6)	1,655 (54.6)	62,875 (52.2)	9,166 (55.4)	5 (55.6)	248,201 (52.2)	758,678 (49.4)
Male	354,304 (51.6)	420,625 (49.8)	172 (49.1)	1,562 (46.1)	325,314 (49.4)	547 (48.4)	1,375 (45.4)	57,551 (47.8)	7,369 (44.6)	<5	226,848 (47.8)	776,663 (50.6)
**Ethnicity, N (%)**												
White	343,722 (50.0)	475,573 (56.3)	171 (48.9)	1,764 (52.1)	372,890 (56.6)	517 (45.8)	1,590 (52.5)	60,310 (50.1)	7,596 (45.9)	<5	284,803 (60.0)	821,230 (53.5)
Indian	16,139 (2.3)	25,223 (3.0)	6 (1.7)	60 (1.8)	20,982 (3.2)	25 (2.2)	54 (1.8)	3,188 (2.6)	346 (2.1)	0	11,109 (2.3)	41,428 (2.7)
Pakistani	26,261 (3.8)	20,630 (2.4)	9 (2.6)	74 (2.2)	13,597 (2.1)	42 (3.7)	64 (2.1)	1,138 (0.9)	143 (0.9)	0	9,633 (2.0)	46,974 (3.1)
Bangladeshi	14,262 (2.1)	13,598 (1.6)	5 (1.4)	34 (1.0)	8,760 (1.3)	26 (2.3)	30 (1.0)	760 (0.6)	102 (0.6)	0	5,492 (1.2)	27,899 (1.8)
Other Asian	16,108 (2.3)	18,126 (2.1)	7 (2.0)	35 (1.0)	13,271 (2.0)	23 (2.0)	32 (1.1)	1,658 (1.4)	227 (1.4)	0	7,956 (1.7)	34,276 (2.2)
Black Caribbean	10,530 (1.5)	2,124 (0.3)	<5	16 (0.5)	1,373 (0.2)	1 (0.1)	14 (0.5)	160 (0.1)	24 (0.1)	0	2,444 (0.5)	12,670 (0.8)
Black African	44,438 (6.5)	17,636 (2.1)	11 (3.1)	64 (1.9)	10,742 (1.6)	40 (3.5)	53 (1.7)	873 (0.7)	122 (0.7)	<5	9,374 (2.0)	62,149 (4.0)
Chinese	3,527 (0.5)	5,004 (0.6)	<5	5 (0.1)	4,047 (0.6)	6 (0.5)	5 (0.2)	602 (0.5)	72 (0.4)	0	1,703 (0.4)	8,538 (0.6)
Other	63,421 (9.2)	36,451 (4.3)	24 (6.9)	129 (3.8)	26,200 (4.0)	54 (4.8)	118 (3.9)	3,374 (2.8)	412 (2.5)	<5	22,676 (4.8)	100,025 (6.5)
Missing	148,747 (21.6)	230,083 (27.2)	115 (32.9)	1,207 (35.6)	186,674 (28.3)	395 (35.0)	1,070 (35.3)	48,363 (40.2)	7,491 (45.3)	<5	119,859 (25.2)	380,152 (24.8)
**Positive SARS-CoV-2 test during study period, N (%)**												
None	542,510 (79.0)	515,477 (61.0)	211 (60.3)	2,094 (61.8)	405,137 (61.5)	661 (58.5)	1,872 (61.8)	78,763 (65.4)	10,274 (62.1)	7 (77.8)	0	1,060,292 (69.1)
Prior to first vaccine dose	144,645 (21.0)	172,464 (20.4)	92 (26.3)	167 (4.9)	129,976 (19.7)	233 (20.6)	142 (4.7)	14,372 (11.9)	1,817 (11.0)	<5	317,368 (66.8)	317,368 (20.7)
After first vaccine dose	0	156,507 (18.5)	47 (13.4)	1,127 (33.3)	123,423 (18.7)	235 (20.8)	1,016 (33.5)	27,291 (22.7)	4,444 (26.9)	<5	157,681 (33.2)	157,681 (10.3)
**Vaccine doses received, N (%)**												
No vaccine	687,155 (100)	-	-	-	-	-	-	-	-	-	144,645 (30.4)	687,155 (44.8)
Dose 1	-	844,448 (100)	350 (100)	3,388 (100)	658,536 (10)	1,129 (100)	3,030 (100)	120,426 (100)	16,535 (100)	9 (100)	330,404 (69.6)	848,186 (55.2)
Dose 2	-	659,227 (78.1)	228 (65.1)	3,240 (95.6)	658,536 (100)	1,129 (100)	3,030 (100)	120,426 (100)	16,535 (100)	9 (100)	255,025 (53.7)	662,695 (43.2)
Dose 3	-	134,571 (15.9)	73 (20.9)	2,326 (68.7)	134,580 (20.4)	151 (13.4)	2,239 (73.9)	120,426 (100)	16,535 (100)	9 (100)	47,926 (10.1)	136,970 (8.9)
**Any comorbidity, N (%)**												
No	581,105 (84.6)	663,093 (78.5)	257 (73.4)	1,286 (38.0)	515,829 (78.3)	862 (76.4)	1,097 (36.2)	82,717 (68.7)	11,964 (72.4)	<5	375,710 (79.1)	1,245,741 (81.1)
Yes	106,050 (15.4)	181,355 (21.5)	93 (26.6)	2,102 (62.0)	142,707 (21.7)	267 (23.6)	1,933 (63.8)	37,709 (31.3)	4,571 (27.6)	5 (55.6)	99,339 (20.9)	289,600 (18.9)
**18-24 years**												
**Total N**	570,619	750,317	91,396	130,054	690,349	82,680	123,985	371,944	187,852	446	405,584	1,542,386
**Mean age (SD)**	21.3 (2.0)	20.3 (1.6)	20.3 (1.7)	20.4 (1.5)	20.2 (1.6)	20.3 (1.6)	20.4 (1.5)	20.3 (1.6)	20.3 (1.6)	20.4 (1.5)	21.8 (1.7)	21.6 (1.8)
**Sex, N (%)**												
Female	261,785 (45.9)	392,093 (52.3)	44,350 (48.5)	78,453 (60.3)	364,386 (52.8)	40,735 (49.3)	74,855 (60.4)	210,441 (56.6)	102,264 (54.4)	283 (63.5)	236,192 (58.2)	776,681 (50.4)
Male	308,834 (54.1)	358,224 (47.7)	47,046 (51.5)	51,601 (39.7)	325,963 (47.2)	41,945 (50.7)	49,130 (39.6)	161,503 (43.4)	85,588 (45.6)	163 (36.5)	169,392 (41.8)	765,705 (49.6)

**Ethnicity, N (%)**												
White	191,698 (33.6)	347,144 (46.3)	42,554 (46.6)	65,080 (50.0)	323,501 (46.9)	38,918 (47.1)	62,134 (50.1)	186,469 (50.1)	96,887 (51.6)	221 (49.6)	193,960 (47.8)	646,476 (41.9)
Indian	9,042 (1.6)	15,363 (2.0)	1,185 (1.3)	3,145 (2.4)	14,476 (2.1)	1,073 (1.3)	3,061 (2.5)	8,428 (2.3)	3,550 (1.9)	14 (3.1)	6,422 (1.6)	28,735 (1.9)
Pakistani	12,240 (2.1)	16,366 (2.2)	1,399 (1.5)	3,089 (2.4)	13,860 (2.0)	1,260 (1.5)	2,881 (2.3)	4,534 (1.2)	1,656 (0.9)	10 (2.2)	6,821 (1.7)	33,094 (2.1)
Bangladeshi	7,399 (1.3)	10,823 (1.4)	1,207 (1.3)	2,066 (1.6)	9,474 (1.4)	997 (1.2)	1,936 (1.6)	3,069 (0.8)	1,460 (0.8)	5 (1.1)	4,902 (1.2)	21,495 (1.4)
Other Asian	10,679 (1.9)	12,632 (1.7)	1,236 (1.4)	1,811 (1.4)	11,526 (1.7)	1,080 (1.3)	1,761 (1.4)	5,399 (1.5)	2,425 (1.3)	6 (1.3)	5,404 (1.3)	26,358 (1.7)
Black Caribbean	8,993 (1.6)	3,211 (0.4)	348 (0.4)	425 (0.3)	2,601 (0.4)	284 (0.3)	389 (0.3)	691 (0.2)	373 (0.2)	0	2,915 (0.7)	12,977 (0.8)
Black African	27,460 (4.8)	16,312 (2.2)	2,130 (2.3)	2,019 (1.6)	13,422 (1.9)	1,675 (2.0)	1,858 (1.5)	3,357 (0.9)	1,617 (0.9)	10 (2.2)	8,450 (2.1)	47,921 (3.1)
Chinese	10,799 (1.9)	3,491 (0.5)	428 (0.5)	293 (0.2)	3,211 (0.5)	361 (0.4)	288 (0.2)	1,956 (0.5)	903 (0.5)	0	1,212 (0.3)	15,011 (1.0)
Other	43,469 (7.6)	28,111 (3.7)	3,458 (3.8)	4,160 (3.2)	24,841 (3.6)	2,910 (3.5)	3,909 (3.2)	10,624 (2.9)	5,269 (2.8)	18 (4.0)	16,386 (4.0)	79,198 (5.1)
Missing	248,840 (43.6)	296,864 (39.6)	37,451 (41.0)	47,966 (36.9)	273,437 (39.6)	34,122 (41.3)	45,768 (36.9)	147,417 (39.6)	73,712 (39.2)	162 (36.3)	159,112 (39.2)	631,121 (40.9)
**Positive SARS-CoV-2 test during study period, N (%)**												
None	498,525 (87.4)	497,699 (66.3)	61,602 (67.4)	78,976 (60.7)	456,348 (66.1)	55,466 (67.1)	75,075 (60.6)	243,244 (65.4)	124,435 (66.2)	264 (59.2)	0	1,136,802 (73.7)
Prior to first vaccine dose	72,094 (12.6)	69,928 (9.3)	10,512 (11.5)	7,985 (6.1)	59,824 (8.7)	8,901 (10.8)	7,608 (6.1)	23,595 (6.3)	13,006 (6.9)	32 (7.2)	160,519 (39.6)	160,519 (10.4)
After first vaccine dose	0	182,690 (24.3)	19,282 (21.1)	43,093 (33.1)	174,177 (25.2)	18,313 (22.1)	41,302 (33.3)	105,105 (28.3)	50,411 (26.8)	150 (33.6)	245,065 (60.4)	245,065 (15.9)
**Vaccine doses received, N (%)**												
No vaccine	570,619 (100)	-	-	-	-	-	-	-	-	-	72,094 (17.8)	570,619 (37.0)
Dose 1	-	750,317 (100)	91,396 (100)	130,054 (100)	690,349 (100)	82,680 (100)	123,985 (100)	371,944 (100)	187,852 (100)	446 (100)	333,490 (82.2)	971,767 (63.0)
Dose 2	-	689,601 (91.9)	80,872 (88.5)	126,541 (97.3)	690,349 (100)	82,680 (100)	123,985 (100)	371,944 (100)	187,852 (100)	446 (100)	310,125 (76.5)	897,014 (58.2)
Dose 3	-	421,862 (56.2)	45,307 (49.6)	93,073 (71.6)	422,351 (61.2)	45,300 (54.8)	92,586 (74.7)	371,944 (100)	187,852 (100)	446 (100)	192,299 (47.4)	560,242 (36.3)
**Any comorbidity, N (%)**												
No	451,013 (79.0)	509,397 (67.9)	63,175 (69.1)	60,032 (46.2)	468,518 (67.9)	57,211 (69.2)	57,223 (46.2)	235,370 (63.3)	122,195 (65.0)	208 (46.6)	261,611 (64.5)	1,083,617 (70.3)
Yes	119,606 (21.0)	240,920 (32.1)	28,221 (30.9)	70,022 (53.8)	221,831 (32.1)	25,469 (30.8)	66,762 (53.8)	136,574 (36.7)	65,657 (35.0)	238 (53.4)	143,973 (35.5)	458,769 (29.7)

Supplementary Table 11. Incidence rates of individual outcomes (events per 100,000 person-years) in the 1-42 days following vaccination or a SARS-CoV-2 positive test (before or after vaccination) in young people aged 5-11, 12-17 and 18-24 years included in QResearch primary care database in England between 8th December 2020 until 7th August 2022. Cells with a hyphen are suppressed; N – number of events; IR – incidence rate; CI – confidence interval.

	BNT162b2 vaccine		mRNA-1273 vaccine		ChAdOX1 vaccine		Positive SARS-CoV-2 test	
	N	Incidence rate (95% CI)	N	Incidence rate (95% CI)	N	Incidence rate (95% CI)	N	Incidence rate (95% CI)
**5-11 years**								
**Multisystem inflammatory syndrome**								
First dose/positive test (before vaccine)	0	-	0	-	0	-	68	75,218 (59,306, 95,399)
Second dose/positive test (after vaccine)	0	-	0	-	0	-	0	-
Third dose	0	-	0	-	0	-		
**Myocarditis**								
First dose/positive test (before vaccine)	0	-	0	-	0	-	<5	-
Second dose/positive test (after vaccine)	0	-	0	-	0	-	0	-
Third dose	0	-	0	-	0	-		
**Immune or idiopathic thrombocytopenia**								
First dose/positive test (before vaccine)	<5	-	0	-	0	-	14	27,418 (16,239, 46,295)
Second dose/positive test (after vaccine)	0	-	0	-	0	-	<5	-
Third dose	0	-	0	-	0	-		
**Epilepsy**								
First dose/positive test (before vaccine)	18	10 (7, 17)	0	-	0	-	27	15,666 (10,743, 22,844)
Second dose/positive test (after vaccine)	7	21 (10, 43)	0	-	0	-	<5	-
Third dose	0	-	0	-	0	-		
**Acute pancreatitis**								
First dose/positive test (before vaccine)	0	-	0	-	0	-	<5	-
Second dose/positive test (after vaccine)	0	-	0	-	0	-	0	-
Third dose	0	-	0	-	0	-		
**Myositis**								
First dose/positive test (before vaccine)	0	-	0	-	0	-	5	41,983 (17,474, 100,865)
Second dose/positive test (after vaccine)	0	-	0	-	0	-	0	-
Third dose	0	-	0	-	0	-		
**Acute disseminated encephalomyelitis**								
First dose/positive test (before vaccine)	0	-	0	-	0	-	<5	-
Second dose/positive test (after vaccine)	0	-	0	-	0	-	0	-
Third dose	0	-	0	-	0	-		
**Demyelinating disease**								
First dose/positive test (before vaccine)	<5	-	0	-	0	-	<5	-
Second dose/positive test (after vaccine)	0	-	0	-	0	-	0	-
Third dose	0	-	0	-	0	-		

**Appendicitis**								
First dose/positive test (before vaccine)	18	10 (7, 16)	0	-	0	-	67	19,230 (15,135, 24,432)
Second dose/positive test (after vaccine)	<5	-	0	-	0	-	0	-
Third dose	0	-	0	-	0	-		
**Guillain-Barre syndrome**								
First dose/positive test (before vaccine)	0	-	0	-	0	-	0	-
Second dose/positive test (after vaccine)	0	-	0	-	0	-	0	-
Third dose	0	-	0	-	0	-		
**Angioedema**								
First dose/positive test (before vaccine)	0	-	0	-	0	-	<5	-
Second dose/positive test (after vaccine)	0	-	0	-	0	-	0	-
Third dose	0	-	0	-	0	-		
**Anaphylaxis**								
First dose/positive test (before vaccine)	<5	-	0	-	0	-	9	35,848 (18,652, 68,897)
Second dose/positive test (after vaccine)	0	-	0	-	0	-	0	-
Third dose	0	-	0	-	0	-		
**12-17 years**								
**Multisystem inflammatory syndrome**								
First dose/positive test (before vaccine)	6	1 (0, 1)	0	-	0	-	37	86,519 (62,687, 119,412)
Second dose/positive test (after vaccine)	0	-	0	-	0	-	<5	-
Third dose	0	-	0	-	0	-		
**Myocarditis**								
First dose/positive test (before vaccine)	8	1 (0, 2)	0	-	0	-	5	37,423 (15,577, 89,910)
Second dose/positive test (after vaccine)	9	1 (1, 2)	0	-	0	-	<5	-
Third dose	<5	-	<5	-	0	-		
**Immune or idiopathic thrombocytopenia**								
First dose/positive test (before vaccine)	12	1 (1, 2)	0	-	0	-	15	26,040 (15,698, 43,193)
Second dose/positive test (after vaccine)	11	2 (1, 3)	0	-	0	-	5	39,788 (16,561, 95,591)
Third dose	<5	-	0	-	0	-		
**Epilepsy**								
First dose/positive test (before vaccine)	60	6 (5, 8)	0	-	5	129 (54, 311)	18	10,305 (6492, 16,356)
Second dose/positive test (after vaccine)	54	7 (6, 10)	<5	-	<5	-	23	33,035 (21,952, 49,712)
Third dose	11	9 (5, 16)	<5	-	0	-		
**Acute pancreatitis**								
First dose/positive test (before vaccine)	8	1 (0, 2)	0	-	0	-	<5	-
Second dose/positive test (after vaccine)	<5	-	0	-	0	-	<5	-
Third dose	<5	-	0	-	0	-		
**Myositis**								
First dose/positive test (before vaccine)	<5	-	0	-	0	-	<5	-
Second dose/positive test (after vaccine)	0	-	0	-	0	-	0	-

Third dose	0	-	0	-	0	-		
**Acute disseminated encephalomyelitis**								
First dose/positive test (before vaccine)	<5	-	0	-	0	-	<5	-
Second dose/positive test (after vaccine)	<5	-	0	-	0	-	<5	-
Third dose	0	-	0	-	0	-		
**Demyelinating disease**								
First dose/positive test (before vaccine)	<5	-	0	-	0	-	<5	-
Second dose/positive test (after vaccine)	<5	-	0	-	0	-	0	-
Third dose	<5	-	0	-	0	-		
**Appendicitis**								
First dose/positive test (before vaccine)	141	15 (12, 17)	0	-	0	-	67	13,305 (10,472, 16,905)
Second dose/positive test (after vaccine)	99	14 (11, 17)	0	-	0	-	28	19,264 (13,301, 27,900)
Third dose	25	20 (14, 30)	<5	-	0	-		
**Guillain-Barre syndrome**								
First dose/positive test (before vaccine)	0	-	0	-	0	-	<5	-
Second dose/positive test (after vaccine)	0	-	0	-	0	-	0	-
Third dose	0	-	0	-	0	-		
**Angioedema**								
First dose/positive test (before vaccine)	8	1 (0, 2)	0	-	0	-	<5	-
Second dose/positive test (after vaccine)	<5	-	0	-	<5	-	0	-
Third dose	<5	-	0	-	0	-		
**Anaphylaxis**								
First dose/positive test (before vaccine)	16	2 (1, 3)	0	-	0	-	6	11,744 (5276, 26,142)
Second dose/positive test (after vaccine)	5	1 (0, 2)	0	-	0	-	<5	-
Third dose	<5	-	0	-	0	-		
**18-24 years**								
**Multisystem inflammatory syndrome**								
First dose/positive test (before vaccine)	<5	-	0	-	0	-	<5	-
Second dose/positive test (after vaccine)	<5	-	0	-	<5	-	<5	-
Third dose	0	-	0	-	0	-		
**Myocarditis**								
First dose/positive test (before vaccine)	9	1 (0, 2)	<5	-	0	-	8	24,637 (12,321, 49,265)
Second dose/positive test (after vaccine)	14	2 (1, 3)	12	13 (7, 23)	<5	-	7	39,887 (19,015, 83,667)
Third dose	6	1 (1, 3)	<5	-	0	-		
**Immune or idiopathic thrombocytopenia**								
First dose/positive test (before vaccine)	16	2 (1, 3)	<5	-	14	9 (6, 16)	16	28,287 (17,329, 46,172)
Second dose/positive test (after vaccine)	18	2 (1, 4)	0	-	<5	-	7	20,069 (9567, 42,096)
Third dose	5	1 (0, 3)	0	-	0	-		
**Epilepsy**								
First dose/positive test (before vaccine)	45	5 (4, 7)	<5	-	59	40 (31, 51)	20	11,132 (7182, 17,255)

Second dose/positive test (after vaccine)	48	6 (5, 8)	<5	-	60	42 (33, 54)	38	26,794 (19,497, 36,824)
Third dose	44	10 (8, 14)	10	5 (3, 9)	<5	-		
**Acute pancreatitis**								
First dose/positive test (before vaccine)	22	3 (2, 4)	5	5 (2, 11)	<5	-	8	23,283 (11,644, 46,557)
Second dose/positive test (after vaccine)	17	2 (1, 3)	<5	-	9	6 (3, 12)	5	9840 (4096, 23,640)
Third dose	9	2 (1, 4)	<5	-	0	-		
**Myositis**								
First dose/positive test (before vaccine)	<5	-	0	-	<5	-	<5	-
Second dose/positive test (after vaccine)	0	-	0	-	<5	-	<5	-
Third dose	0	-	0	-	0	-		
**Acute disseminated encephalomyelitis**								
First dose/positive test (before vaccine)	7	1 (0, 2)	0	-	0	-	<5	-
Second dose/positive test (after vaccine)	<5	-	0	-	<5	-	<5	-
Third dose	0	-	<5	-	0	-		
**Demyelinating disease**								
First dose/positive test (before vaccine)	<5	-	0	-	0	-	<5	-
Second dose/positive test (after vaccine)	5	1 (0, 2)	0	-	0	-	<5	-
Third dose	<5	-	0	-	0	-		
**Appendicitis**								
First dose/positive test (before vaccine)	141	16 (14, 19)	20	19 (12, 30)	34	23 (16, 32)	33	11,445 (8137, 16,099)
Second dose/positive test (after vaccine)	121	15 (13, 18)	14	15 (9, 25)	29	20 (14, 29)	58	25,527 (19,734, 33,019)
Third dose	64	15 (12, 19)	26	13 (9, 18)	0	-		
**Guillain-Barre syndrome**								
First dose/positive test (before vaccine)	0	-	0	-	0	-	0	-
Second dose/positive test (after vaccine)	<5	-	0	-	0	-	0	-
Third dose	0	-	0	-	0	-		
**Angioedema**								
First dose/positive test (before vaccine)	8	1 (0, 2)	<5	-	<5	-	<5	-
Second dose/positive test (after vaccine)	7	1 (0, 2)	0	-	<5	-	<5	-
Third dose	<5	-	<5	-	0	-		
**Anaphylaxis**								
First dose/positive test (before vaccine)	8	1 (0, 2)	0	-	7	5 (2, 10)	<5	-
Second dose/positive test (after vaccine)	9	1 (0, 2)	<5	-	6	4 (2, 9)	<5	-
Third dose	7	2 (1, 3)	<5	-	0	-		

Supplementary Table 12. Characteristics of matched vaccinated and unvaccinated young people aged 5-11, 12-17 and 18-24 years included in matched cohort study included in QResearch primary care database in England between 8th December 2020 until 7th August 2022. Cells with <5 are suppressed. Data are presented as counts (column %).

	Dose 1		Dose 2		Dose 3	
	Vaccinated with first dose	Matched unvaccinated	Vaccinated with second dose	Matched unvaccinated	Vaccinated with third dose	Matched unvaccinated
**5-11 years**						
**Total N**	160,262	160,262	78,374	78,374	200	200
**Mean age (SD)**	8.7 (2.0)	8.7 (2.0)	8.8 (1.9)	8.8 (1.9)	10.0 (1.7)	10.0 (1.7)
**Sex, N (%)**						
Female	77,942 (48.6)	77,942 (48.6)	38,028 (48.5)	38,028 (48.5)	82 (41.0)	82 (41.0)
Male	82,320 (51.4)	82,320 (51.4)	40,346 (51.5)	40,346 (51.5)	118 (59.0)	118 (59.0)
**Ethnicity, N (%)**						
White	90,655 (56.6)	83,113 (51.9)	44,946 (57.3)	40,553 (51.7)	141 (70.5)	108 (54.0)
Indian	8,730 (5.4)	4,196 (2.6)	4,413 (5.6)	1,959 (2.5)	<5	<5
Pakistani	2,469 (1.5)	4,909 (3.1)	959 (1.2)	2,426 (3.1)	<5	<5
Bangladeshi	2,346 (1.5)	2,790 (1.7)	1,064 (1.4)	1,448 (1.8)	<5	<5
Other Asian	3,446 (2.2)	3,270 (2.0)	1,595 (2.0)	1,585 (2.0)	<5	<5
Black Caribbean	176 (0.1)	1,086 (0.7)	83 (0.1)	574 (0.7)	<5	<5
Black African	1,977 (1.2)	5,810 (3.6)	859 (1.1)	3,018 (3.9)	<5	12 (6.0)
Chinese	1,628 (1.0)	861 (0.5)	896 (1.1)	430 (0.5)	<5	<5
Other	7,374 (4.6)	11,098 (6.9)	3,567 (4.6)	5,502 (7.0)	8 (4.0)	15 (7.5)
Missing	41,461 (25.9)	43,129 (26.9)	19,992 (25.5)	20,879 (26.6)	45 (22.5)	46 (23.0)
**Vaccine type, N (%)**						
BNT162b2	160,208 (>99.9)	-	78,363 (>99.9)	-	200 (100.0)	-
mRNA-1273	20 (<0.1)	-	9 (<0.1)	-	0	-
ChAdOX1	34 (<0.1)	-	<5	-	0	-
**12-17 years**						
**Total N**	848,186	848,186	480,558	480,558	75,479	75,479
**Mean age (SD)**	14.6 (1.8)	14.6 (1.8)	14.7 (1.8)	14.7 (1.8)	16.1 (1.1)	16.1 (1.1)
**Sex, N (%)**						
Female	425,827 (50.2)	425,827 (50.2)	240,656 (50.1)	240,656 (50.1)	38,166 (50.6)	38,166 (50.6)
Male	422,359 (49.8)	422,359 (49.8)	239,902 (49.9)	239,902 (49.9)	37,313 (49.4)	37,313 (49.4)
**Ethnicity, N (%)**						
White	477,508 (56.3)	414,906 (48.9)	271,443 (56.5)	222,149 (46.2)	37,423 (49.6)	28,771 (38.1)
Indian	25,289 (3.0)	19,447 (2.3)	15,095 (3.1)	10,216 (2.1)	1,964 (2.6)	1,327 (1.8)
Pakistani	20,713 (2.4)	28,652 (3.4)	10,309 (2.1)	17,209 (3.6)	717 (0.9)	2,488 (3.3)
Bangladeshi	13,637 (1.6)	16,552 (2.0)	6,553 (1.4)	9,527 (2.0)	457 (0.6)	1,398 (1.9)

Other Asian	18,168 (2.1)	18,844 (2.2)	9,629 (2.0)	10,625 (2.2)	1,071 (1.4)	1,499 (2.0)
Black Caribbean	2,140 (0.3)	10,085 (1.2)	1,042 (0.2)	7,129 (1.5)	105 (0.1)	1,242 (1.6)
Black African	17,711 (2.1)	43,323 (5.1)	8,160 (1.7)	28,668 (6.0)	556 (0.7)	4,614 (6.1)
Chinese	5,011 (0.6)	4,401 (0.5)	2,894 (0.6)	2,452 (0.5)	350 (0.5)	399 (0.5)
Other	36,604 (4.3)	64,112 (7.6)	19,336 (4.0)	41,044 (8.5)	2,084 (2.8)	6,335 (8.4)
Missing	231,405 (27.3)	227,864 (26.9)	136,097 (28.3)	131,539 (27.4)	30,752 (40.7)	27,406 (36.3)
**Vaccine type, N (%)**						
BNT162b2	844,448 (99.6)	-	476,862 (99.2)	-	66,303 (87.8)	-
mRNA-1273	350 (<0.1)	-	850 (0.2)	-	9,169 (12.1)	-
ChAdOX1	3,388 (0.4)	-	2,846 (0.6)	-	7 (<0.1)	-
**18-24 years**						
**Total N**	971,767	971,767	676,841	676,841	297,405	297,405
**Mean age (SD)**	20.3 (1.6)	20.3 (1.6)	20.3 (1.6)	20.3 (1.6)	20.4 (1.6)	20.4 (1.6)
**Sex, N (%)**						
Female	514,896 (53.0)	514,896 (53.0)	360,235 (53.2)	360,235 (53.2)	159,134 (53.5)	159,134 (53.5)
Male	456,871 (47.0)	456,871 (47.0)	316,606 (46.8)	316,606 (46.8)	138,271 (46.5)	138,271 (46.5)
**Ethnicity, N (%)**						
White	454,778 (46.8)	373,409 (38.4)	318,410 (47.0)	245,119 (36.2)	149,813 (50.4)	101,020 (34.0)
Indian	19,693 (2.0)	15,949 (1.6)	13,812 (2.0)	10,764 (1.6)	6,045 (2.0)	4,531 (1.5)
Pakistani	20,854 (2.1)	22,386 (2.3)	14,149 (2.1)	16,170 (2.4)	3,184 (1.1)	6,773 (2.3)
Bangladeshi	14,096 (1.5)	13,905 (1.4)	9,643 (1.4)	9,579 (1.4)	2,382 (0.8)	3,757 (1.3)
Other Asian	15,679 (1.6)	17,490 (1.8)	10,952 (1.6)	12,514 (1.8)	4,145 (1.4)	5,571 (1.9)
Black Caribbean	3,984 (0.4)	11,099 (1.1)	2,640 (0.4)	9,137 (1.3)	579 (0.2)	4,598 (1.5)
Black African	20,461 (2.1)	37,441 (3.9)	13,647 (2.0)	29,109 (4.3)	2,625 (0.9)	13,458 (4.5)
Chinese	4,212 (0.4)	13,547 (1.4)	2,849 (0.4)	11,375 (1.7)	1,579 (0.5)	6,227 (2.1)
Other	35,729 (3.7)	59,556 (6.1)	24,415 (3.6)	46,001 (6.8)	8,484 (2.9)	22,283 (7.5)
Missing	382,281 (39.3)	406,985 (41.9)	266,324 (39.3)	287,073 (42.4)	118,569 (39.9)	129,187 (43.4)
**Vaccine type, N (%)**						
BNT162b2	750,317 (77.2)	-	505,781 (74.7)	-	194,911 (65.5)	-
mRNA-1273	91,396 (9.4)	-	62,699 (9.3)	-	102,247 (34.4)	-
ChAdOX1	130,054 (13.4)	-	108,361 (16.0)	-	247 (0.1)	-

Supplementary Table 13. Incidence rate ratios (unadjusted and adjusted for ethnicity, quintile of deprivation and comorbidities) for individual outcomes in the 1-42 days following vaccination compared to matched unvaccinated group in young people aged 5-11, 12-17 and 18-24 years. Cells with a hyphen are suppressed and cells with * are models that did not converge. N – number of events; IRR – incidence rate ratio; CI – confidence interval.

	Unvaccinated	BNT162b2 vaccine			mRNA-1273 vaccine			ChAdOX1 vaccine		
	N events	N events	Unadjusted IRR (95%CI)	Adjusted IRR (95%CI)	N events	Unadjusted IRR (95%CI)	Adjusted IRR (95%CI)	N events	Unadjusted IRR (95%CI)	Adjusted IRR (95%CI)
**5-11 years**										
**Multisystem inflammatory syndrome**										
First dose/positive test (before vaccine)	0	0	-	-	0	-	-	0	-	-
Second dose/positive test (after vaccine)	0	0	-	-	0	-	-	0	-	-
Third dose	0	0	-	-	0	-	-	0	-	-
**Myocarditis**										
First dose/positive test (before vaccine)	0	0	-	-	0	-	-	0	-	-
Second dose/positive test (after vaccine)	0	0	-	-	0	-	-	0	-	-
Third dose	0	0	-	-	0	-	-	0	-	-
**Immune or idiopathic thrombocytopenia**										
First dose/positive test (before vaccine)	<5	<5	-	-	0	-	-	0	-	-
Second dose/positive test (after vaccine)	0	0	-	-	0	-	-	0	-	-
Third dose	0	0	-	-	0	-	-	0	-	-
**Epilepsy**										
First dose/positive test (before vaccine)	<5	16	16.00 (2.12, 120.65)	*	0	-	-	0	-	-
Second dose/positive test (after vaccine)	<5	7	7.00 (0.86, 56.89)	*	0	-	-	0	-	-
Third dose	0	0	-	-	0	-	-	0	-	-
**Acute pancreatitis**										
First dose/positive test (before vaccine)	0	0	-	-	0	-	-	0	-	-
Second dose/positive test (after vaccine)	0	0	-	-	0	-	-	0	-	-
Third dose	0	0	-	-	0	-	-	0	-	-
**Myositis**										
First dose/positive test (before vaccine)	0	0	-	-	0	-	-	0	-	-

Second dose/positive test (after vaccine)	0	0	-	-	0	-	-	0	-	-
Third dose	0	0	-	-	0	-	-	0	-	-
**Acute disseminated encephalomyelitis**										
First dose/positive test (before vaccine)	<5	0	-	-	0	-	-	0	-	-
Second dose/positive test (after vaccine)	0	0	-	-	0	-	-	0	-	-
Third dose	0	0	-	-	0	-	-	0	-	-
**Demyelinating disease**										
First dose/positive test (before vaccine)	0	<5	-	-	0	-	-	0	-	-
Second dose/positive test (after vaccine)	0	0	-	-	0	-	-	0	-	-
Third dose	0	0	-	-	0	-	-	0	-	-
**Appendicitis**										
First dose/positive test (before vaccine)	20	18	0.89 (0.47, 1.68)	0.96 (0.45, 2.03)	0	-	-	0	-	-
Second dose/positive test (after vaccine)	<5	<5	-	-	0	-	-	0	-	-
Third dose	0	0	-	-	0	-	-	0	-	-
**Guillain-Barre syndrome**										
First dose/positive test (before vaccine)	0	0	-	-	0	-	-	0	-	-
Second dose/positive test (after vaccine)	0	0	-	-	0	-	-	0	-	-
Third dose	0	0	-	-	0	-	-	0	-	-
**Angioedema**										
First dose/positive test (before vaccine)	0	0	-	-	0	-	-	0	-	-
Second dose/positive test (after vaccine)	0	0	-	-	0	-	-	0	-	-
Third dose	0	0	-	-	0	-	-	0	-	-
**Anaphylaxis**										
First dose/positive test (before vaccine)	0	<5	-	-	0	-	-	0	-	-
Second dose/positive test (after vaccine)	0	0	-	-	0	-	-	0	-	-
Third dose	0	0	-	-	0	-	-	0	-	-
**12-17 years**										
**Multisystem inflammatory syndrome**										
First dose/positive test (before vaccine)	6	6	0.99 (0.32, 3.07)	*	0	-	-	0	-	-

Second dose/positive test (after vaccine)	<5	0	-	-	0	-	-	0	-	-
Third dose	0	0	-	-	0	-	-	0	-	-
**Myocarditis**										
First dose/positive test (before vaccine)	<5	8	2.64 (0.70, 9.94)	*	0	-	-	0	-	-
Second dose/positive test (after vaccine)	<5	9	2.00 (0.50, 8.00)	*	0	-	-	0	-	-
Third dose	0	<5	-	-	<5	-	-	0	-	-
**Immune or idiopathic thrombocytopenia**										
First dose/positive test (before vaccine)	17	12	0.64 (0.30, 1.35)	1.13 (0.33, 3.91)	0	-	-	0	-	-
Second dose/positive test (after vaccine)	8	10	0.75 (0.26, 2.16)	*	0	-	-	0	-	-
Third dose	<5	<5	-	-	0	-	-	0	-	-
**Epilepsy**										
First dose/positive test (before vaccine)	42	59	1.34 (0.90, 1.99)	1.32 (0.78, 2.24)	0	-	-	5	*	*
Second dose/positive test (after vaccine)	22	51	1.77 (1.05, 2.99)	3.88 (1.27, 11.86)	<5	-	-	<5	-	-
Third dose	<5	11	2.67 (0.71, 10.05)	1.00 (0.06, 15.99)	<5	-	-	0	-	-
**Acute pancreatitis**										
First dose/positive test (before vaccine)	9	8	0.86 (0.33, 2.24)	6.78 (0.38, 122.00)	0	-	-	0	-	-
Second dose/positive test (after vaccine)	0	<5	-	-	0	-	-	0	-	-
Third dose	0	<5	-	-	0	-	-	0	-	-
**Myositis**										
First dose/positive test (before vaccine)	<5	<5	-	-	0	-	-	0	-	-
Second dose/positive test (after vaccine)	<5	0	-	-	0	-	-	0	-	-
Third dose	0	0	-	-	0	-	-	0	-	-
**Acute disseminated encephalomyelitis**										
First dose/positive test (before vaccine)	<5	<5	-	-	0	-	-	0	-	-
Second dose/positive test (after vaccine)	0	<5	-	-	0	-	-	0	-	-
Third dose	<5	0	-	-	0	-	-	0	-	-
**Demyelinating disease**										
First dose/positive test (before vaccine)	0	<5	-	-	0	-	-	0	-	-

Second dose/positive test (after vaccine)	0	<5	-	-	0	-	-	0	-	-
Third dose	0	<5	-	-	0	-	-	0	-	-
**Appendicitis**										
First dose/positive test (before vaccine)	88	136	1.37 (1.05, 1.80)	1.26 (0.94, 1.69)	0	-	-	0	-	-
Second dose/positive test (after vaccine)	58	97	1.21 (0.85, 1.71)	1.07 (0.72, 1.59)	0	-	-	0	-	-
Third dose	5	25	2.07 (0.72, 6.01)	*	<5	-	-	0	-	-
**Guillain-Barre syndrome**										
First dose/positive test (before vaccine)	0	0	-	-	0	-	-	0	-	-
Second dose/positive test (after vaccine)	0	0	-	-	0	-	-	0	-	-
Third dose	0	0	-	-	0	-	-	0	-	-
**Angioedema**										
First dose/positive test (before vaccine)	<5	8	1.79 (0.53, 6.07)	*	0	-	-	0	-	-
Second dose/positive test (after vaccine)	<5	<5	-	-	0	-	-	<5	-	-
Third dose	<5	<5	-	-	0	-	-	0	-	-
**Anaphylaxis**										
First dose/positive test (before vaccine)	<5	16	3.71 (1.23, 11.14)	14.17 (0.63, 316.28)	0	-	-	0	-	-
Second dose/positive test (after vaccine)	<5	5	0.75 (0.17, 3.35)	*	0	-	-	0	-	-
Third dose	<5	<5	-	-	0	-	-	0	-	-
**18-24 years**										
**Multisystem inflammatory syndrome**										
First dose/positive test (before vaccine)	<5	<5	-	-	0	-	-	0	-	-
Second dose/positive test (after vaccine)	<5	<5	-	-	0	-	-	<5	-	-
Third dose	0	0	-	-	0	-	-	0	-	-
**Myocarditis**										
First dose/positive test (before vaccine)	8	9	2.36 (0.72, 7.68)	*	<5	-	-	0	-	-
Second dose/positive test (after vaccine)	6	14	1.67 (0.61, 4.60)	*	11	*	*	<5	-	-
Third dose	0	6	*	*	<5	-	-	0	-	-
**Immune or idiopathic thrombocytopenia**										
First dose/positive test (before vaccine)	17	16	1.13 (0.54, 2.36)	1.22 (0.45, 3.30)	<5	-	-	13	13.18 (1.72, 100.79)	16.62 (1.27, 217.31)

Second dose/positive test (after vaccine)	26	18	0.65 (0.34, 1.25)	0.74 (0.31, 1.75)	0	-	-	<5	-	-
Third dose	8	5	0.50 (0.13, 2.00)	*	0	-	-	0	-	-
**Epilepsy**										
First dose/positive test (before vaccine)	56	43	0.98 (0.63, 1.50)	0.99 (0.58, 1.71)	<5	-	-	59	5.25 (2.75, 10.00)	4.20 (1.87, 9.42)
Second dose/positive test (after vaccine)	43	48	1.08 (0.67, 1.74)	1.06 (0.57, 1.97)	<5	-	-	60	5.30 (2.61, 10.77)	5.72 (2.32, 14.11)
Third dose	15	41	1.83 (0.93, 3.60)	0.97 (0.30, 3.16)	10	1.50 (0.25, 8.98)	7.50 (0.53, 105.95)	0	-	-
**Acute pancreatitis**										
First dose/positive test (before vaccine)	16	22	1.96 (0.94, 4.09)	5.56 (1.30, 23.72)	5	1.67 (0.40, 6.97)	13.23 (1.26, 139.48)	<5	-	-
Second dose/positive test (after vaccine)	9	17	2.19 (0.76, 6.30)	6.26 (0.92, 42.50)	<5	-	-	9	2.79 (0.75, 10.35)	2.18 (0.11, 44.14)
Third dose	5	9	0.50 (0.09, 2.73)	*	<5	-	-	0	-	-
**Myositis**										
First dose/positive test (before vaccine)	<5	<5	-	-	0	-	-	<5	-	-
Second dose/positive test (after vaccine)	<5	0	-	-	0	-	-	<5	-	-
Third dose	0	0	-	-	0	-	-	0	-	-
**Acute disseminated encephalomyelitis**										
First dose/positive test (before vaccine)	<5	7	2.01 (0.51, 7.87)	3.38 (0.24, 47.13)	0	-	-	0	-	-
Second dose/positive test (after vaccine)	<5	<5	-	-	0	-	-	<5	-	-
Third dose	0	0	-	-	<5	-	-	0	-	-
**Demyelinating disease**										
First dose/positive test (before vaccine)	5	<5	-	-	0	-	-	0	-	-
Second dose/positive test (after vaccine)	<5	5	1.65 (0.39, 6.91)	*	0	-	-	0	-	-
Third dose	0	<5	-	-	0	-	-	0	-	-
**Appendicitis**										
First dose/positive test (before vaccine)	109	137	1.45 (1.11, 1.91)	1.35 (1.02, 1.79)	20	2.27 (1.00, 5.19)	2.56 (1.04, 6.32)	34	1.90 (1.04, 3.47)	1.93 (1.03, 3.60)
Second dose/positive test (after vaccine)	61	119	2.06 (1.41, 3.00)	1.89 (1.26, 2.83)	14	1.88 (0.70, 5.04)	1.56 (0.52, 4.66)	27	1.56 (0.82, 2.96)	1.47 (0.74, 2.92)
Third dose	17	63	2.58 (1.36, 4.88)	3.81 (1.56, 9.29)	24	2.99 (0.97, 9.28)	5.25 (1.22, 22.53)	0	-	-
**Guillain-Barre syndrome**										
First dose/positive test (before vaccine)	<5	0	-	-	0	-	-	0	-	-

Second dose/positive test (after vaccine)	0	<5	-	-	0	-	-	0	-	-
Third dose	<5	0	-	-	0	-	-	0	-	-
**Angioedema**										
First dose/positive test (before vaccine)	9	7	0.68 (0.22, 2.07)	25.89 (0.24, 2768.04)	<5	-	-	<5	-	-
Second dose/positive test (after vaccine)	<5	7	2.00 (0.50, 8.00)	*	0	-	-	<5	-	-
Third dose	<5	<5	-	-	<5	-	-	0	-	-
**Anaphylaxis**										
First dose/positive test (before vaccine)	8	8	1.24 (0.43, 3.61)	1.45 (0.28, 7.61)	0	-	-	7	6.14 (0.75, 50.40)	5.86 (0.37, 93.30)
Second dose/positive test (after vaccine)	6	9	1.67 (0.40, 6.97)	*	<5	-	-	6	*	*
Third dose	<5	7	1.33 (0.30, 5.96)	*	<5	-	-	0	-	-
