# Supplementary material for: Safety outcomes following COVID-19 vaccination and infection in 5.1 million children in England
Source: Nat Commun. 2024 May 27;15:3822. doi: 10.1038/s41467-024-47745-z (PMC11130197; doi:10.1038/s41467-024-47745-z)
Supplement: Supplementary file 3 — Reporting Summary [file 41467_2024_47745_MOESM3_ESM.pdf]

nature portfolio

Corresponding author(s): Julia Hippisley-Cox

Last updated by author(s): Apr 3, 2024

Reporting Summary

Nature Portfolio wishes to improve the reproducibility of the work that we publish. This form provides structure for consistency and transparency in reporting. For further information on Nature Portfolio policies, see our Editorial Policies and the Editorial Policy Checklist.

Statistics

For all statistical analyses, confirm that the following items are present in the figure legend, table legend, main text, or Methods section.

n/a	Confirmed	
☐	☒	The exact sample size (n) for each experimental group/condition, given as a discrete number and unit of measurement
☒	☐	A statement on whether measurements were taken from distinct samples or whether the same sample was measured repeatedly
☒	☐	The statistical test(s) used AND whether they are one- or two-sided<br>*Only common tests should be described solely by name; describe more complex techniques in the Methods section.*
☐	☒	A description of all covariates tested
☐	☒	A description of any assumptions or corrections, such as tests of normality and adjustment for multiple comparisons
☐	☒	A full description of the statistical parameters including central tendency (e.g. means) or other basic estimates (e.g. regression coefficient) AND variation (e.g. standard deviation) or associated estimates of uncertainty (e.g. confidence intervals)
☒	☐	For null hypothesis testing, the test statistic (e.g. F, t, r) with confidence intervals, effect sizes, degrees of freedom and P value noted<br>*Give P values as exact values whenever suitable.*
☒	☐	For Bayesian analysis, information on the choice of priors and Markov chain Monte Carlo settings
☒	☐	For hierarchical and complex designs, identification of the appropriate level for tests and full reporting of outcomes
☒	☐	Estimates of effect sizes (e.g. Cohen's d, Pearson's r), indicating how they were calculated

Our web collection on statistics for biologists contains articles on many of the points above.

Software and code

Policy information about availability of computer code

Data collection	No software was used.
Data analysis	Stata version 17 was used for data analysis.

For manuscripts utilizing custom algorithms or software that are central to the research but not yet described in published literature, software must be made available to editors and reviewers. We strongly encourage code deposition in a community repository (e.g. GitHub). See the Nature Portfolio guidelines for submitting code & software for further information.

Data

Policy information about availability of data

All manuscripts must include a data availability statement. This statement should provide the following information, where applicable:

- Accession codes, unique identifiers, or web links for publicly available datasets
- A description of any restrictions on data availability
- For clinical datasets or third party data, please ensure that the statement adheres to our policy

The data that support the findings of this study—NIMS Database of COVID-19, mortality (Office of National Statistics), hospital admissions (Hospital Episode Statistics) and SARS-CoV-2 infection data (PHE)—are not publicly available because they are based on deidentified national clinical records. Due to national and organizational data privacy regulations, individual-level data such as those used for this study cannot be shared openly.

Research involving human participants, their data, or biological material

Policy information about studies with human participants or human data. See also policy information about sex, gender (identity/presentation), and sexual orientation and race, ethnicity and racism.

Reporting on sex and gender	All analyses have been stratified by sex and results have been reported. We do not have information on gender in our dataset. We used sex recorded on an individual's GP or hospital record.
Reporting on race, ethnicity, or other socially relevant groupings	We have described the cohort by ethnicity. We used ethnicity recorded on an individual's GP or hospital record. Ethnicity was grouped into nine categories (White, Indian, Pakistani, Bangladeshi, Other Asian, Black Caribbean, Black African, Chinese, Other) and we reported the number of people with no ethnicity recorded. Ethnicity was inherently controlled for in the self-controlled case series method.
Population characteristics	We included all children aged 5-17 years who had received at least one dose of BNT162b2, mRNA-1273 or ChAdOx1 vaccine or had a positive SARS-CoV-2 test and were admitted to hospital or died from at least one of the outcomes between 8th December 2020 and 7th August 2022. We also undertook an analysis in young adults aged 18-24 years as a comparison. We also conducted a matched cohort study using the QResearch database of primary care records, linked to hospital episode statistics, COVID-19 vaccination and SARS-CoV-2 infection data.
Recruitment	No participants were recruited as we used routinely collected data.
Ethics oversight	The QResearch® ethics approval was provided by the East Midlands-Derby Research Ethics Committee [reference 18/EM/0400] and reviewed by the QResearch science committee [Project OX167]. Consent from participants was not required. The study was performed in accordance with the Declaration of Helsinki.

Note that full information on the approval of the study protocol must also be provided in the manuscript.

Field-specific reporting

Please select the one below that is the best fit for your research. If you are not sure, read the appropriate sections before making your selection.

☒ Life sciences ☐ Behavioural & social sciences ☐ Ecological, evolutionary & environmental sciences

For a reference copy of the document with all sections, see nature.com/documents/nr-reporting-summary-flat.pdf

Life sciences study design

All studies must disclose on these points even when the disclosure is negative.

Sample size	The sample size was 5,197,925 children. We included all children aged 5-17 vaccinated or with a positive SARS-CoV-2 test in England in the study period.
Data exclusions	For the analysis of each outcome, we excluded children with a hospitalization for the same outcome in the two years prior to 8th December 2020 and those who received other COVID-19 vaccine types. We excluded people who received a COVID-19 vaccine other than ChAdOX1, BNT162b2 or mRNA-1273.
Replication	We did not replicate this analysis using a different dataset. Replication was not applicable as we conducted an observational study.
Randomization	This was an observational study, therefore the exposure groups were not randomized. Our main vaccine exposures were a first, second and third dose of BNT162b2, mRNA-1273 or ChAdOx1. To account for heterologous vaccination, each vaccine type and dose was considered separately. SARS-CoV-2 infection exposure was defined as the first positive SARS-CoV-2 test (assessed by RT-PCR) within the study period. By using the self-controlled case series method, we inherently controlled for fixed characteristics during the study period, such as sex and ethnicity. Age was considered as a fixed variable because the study period was short.
Blinding	Blinding was not relevant to this study as we used routinely collected data and this was an observational study.

Reporting for specific materials, systems and methods

We require information from authors about some types of materials, experimental systems and methods used in many studies. Here, indicate whether each material, system or method listed is relevant to your study. If you are not sure if a list item applies to your research, read the appropriate section before selecting a response.

Materials & experimental systems

n/a	Involved in the study
☒	☐ Antibodies
☒	☐ Eukaryotic cell lines
☒	☐ Palaeontology and archaeology
☒	☐ Animals and other organisms
☐	☒ Clinical data
☒	☐ Dual use research of concern
☒	☐ Plants

Methods

n/a	Involved in the study
☒	☐ ChIP-seq
☒	☐ Flow cytometry
☒	☐ MRI-based neuroimaging

Clinical data

Policy information about clinical studies

All manuscripts should comply with the ICMJE guidelines for publication of clinical research and a completed CONSORT checklist must be included with all submissions.

Clinical trial registration

This study was not a clinical trial.

Study protocol

Details of the study can be found on the QResearch website (https://www.qresearch.org/research/research-programs-and-projects/covid-19-vaccination-in-children-in-england-uptake-safety-and-effectiveness/#h-H2_11)

Data collection

QResearch is a large consolidated database derived from the anonymized health records from general practices using the EMIS clinical computer system. The study period was 8th December 2020 to 7th August 2022, and we records for co-morbidities from the two years prior to the study start date.

Outcomes

We selected severe outcomes resulting in hospital admission or death which are monitored by national medical regulatory authorities, clinical trials, post-marketing surveillance and emerging scientific literature. Our predefined outcomes were: myocarditis, multi-system inflammatory syndrome, immune thrombocytopenia, hospitalization with epilepsy, acute pancreatitis, acute disseminated encephalomyelitis, Guillain-Barre syndrome, appendicitis, demyelinating disease, myositis, angioedema and anaphylaxis. Outcomes were identified using relevant International Classification of Diseases codes (https://www.qresearch.org/data/qcode-group-library/). We used the earliest date of hospitalization or date of death in the study period as the event date.
